# Cellular delivery and photochemical release of a caged inositol-pyrophosphate induces PH-domain translocation *in cellulo*

**DOI:** 10.1038/ncomms10622

**Published:** 2016-02-04

**Authors:** Igor Pavlovic, Divyeshsinh T. Thakor, Jessica R. Vargas, Colin J. McKinlay, Sebastian Hauke, Philipp Anstaett, Rafael C. Camuña, Laurent Bigler, Gilles Gasser, Carsten Schultz, Paul A. Wender, Henning J. Jessen

**Affiliations:** 1 Department of Chemistry, University of Zurich, Winterthurerstrasse 190, Zurich 8057, Switzerland; 2 Departments of Chemistry and Chemical and Systems Biology, Stanford University, Stanford, California 94305, USA; 3 European Molecular Biology Laboratory (EMBL), Cell Biology & Biophysics Unit, Meyerhofstrasse 1, 69117 Heidelberg, Germany; 4 Departamento de Química Orgánica, Facultad de Ciencias, Universidad de Málaga, Malaga 29071, Spain; 5 Department of Chemistry and Pharmacy, Albert-Ludwigs University Freiburg, Albertstrasse 21, 79104 Freiburg, Germany

## Abstract

Inositol pyrophosphates, such as diphospho-myo-inositol pentakisphosphates (InsP_7_), are an important family of signalling molecules, implicated in many cellular processes and therapeutic indications including insulin secretion, glucose homeostasis and weight gain. To understand their cellular functions, chemical tools such as photocaged analogues for their real-time modulation in cells are required. Here we describe a concise, modular synthesis of InsP_7_ and caged InsP_7_. The caged molecule is stable and releases InsP_7_ only on irradiation. While photocaged InsP_7_ does not enter cells, its cellular uptake is achieved using nanoparticles formed by association with a guanidinium-rich molecular transporter. This novel synthesis and unprecedented polyphosphate delivery strategy enable the first studies required to understand InsP_7_ signalling in cells with controlled spatiotemporal resolution. It is shown herein that cytoplasmic photouncaging of InsP_7_ leads to translocation of the PH-domain of Akt, an important signalling-node kinase involved in glucose homeostasis, from the membrane into the cytoplasm.

## Abstract (web summary)

Photocaged inositol-pyrophosphates offer a tool to study cellular signalling, but their challenging synthesis has precluded any biological studies so far. Here, the authors report the synthesis and cellular delivery of a photocaged analogue, and show that it mediates protein translocation *in cellulo*.

## Graphical abstract

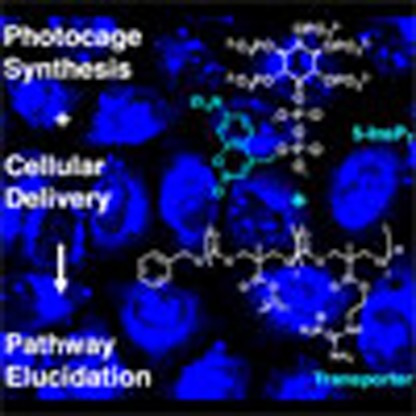

Diphospho-inositol polyphosphates (InsP_7_) are second messengers involved in essential cell signalling pathways^1^,^2^,^3^,^4^. A distinct difference of InsP_7_ compared with other inositol polyphosphates is the presence of a phosphoanhydride bond in, for example, the 5-position (5-InsP_7_, Fig. 1), rendering them a structurally unique class of second messengers. This special feature is also the reason for their nickname ‘inositol pyrophosphates'. InsP_7_ are implicated in the regulation of diverse cellular and metabolic functions in different kingdoms of life^1^,^2^,^3^,^4^,^5^,^6^,^7^,^8^. It has been proposed that InsP_7_ bind to the pleckstrin homology (PH) domain of protein kinase B (Akt), and competitively suppress its specific phosphatidylinositol 3,4,5-trisphosphate (PIP_3_) association at the plasma membrane, thereby inhibiting phosphoinositide-dependent kinase 1 (PDK1)-mediated phosphorylation of Akt^9^,^10^. However, there remains uncertainty as to whether the reduced phosphorylation of Akt is a result of the inhibition of its membrane association via its PH-domain, since the *in vitro* assays that have been performed do not contain any membrane or membrane mimics. In addition, InsP_7_ might act either as allosteric inhibitors or as non-enzymatic phosphorylating agents or both^3^,^11^. Notwithstanding, inhibition of the Akt pathway by InsP_7_ has an impact on glucose uptake and insulin sensitivity, as exemplified by a mouse model that lacks inositol hexakisphosphate-kinase 1 (IP6K1). These knockout mice have reduced levels of InsP_7_ and show a lean phenotype on high-fat diet concomitant with increased insulin sensitivity^9^. As a consequence, IP6K1 has recently been proposed as a novel target in the treatment of diabetes and obesity^12^. To address fundamental questions about the mechanism of action of these potent signalling molecules and their subcellular localization, the development of new chemical tools is required.

To understand cellular signalling mediated by second messengers, photocaged analogues that can be activated on demand inside living cells with spatiotemporal resolution have attracted great interest^13^. Unfortunately, preparation of such analogues often requires lengthy synthetic sequences. Phosphorylated second messengers derived from *myo-*inositol such as *myo-*inositol 1,4,5-trisphosphate (Fig. 1) present additional challenges as their polyanionic nature precludes efficient cellular uptake^14^. For example, while cell-permeable and photocaged analogues of different *myo*-inositol polyphosphates (InsP_*x*_) and phosphatidyl *myo*-inositol polyphosphates (PtdIns-P_*x*_) have been reported^15^,^16^,^17^,^18^, the phosphate groups typically need to be reversibly masked. This complicates their use, as multiple intracellular hydrolysis events must occur before the free polyphosphate is formed^19^. Even so, no such photocaged derivatives are currently available for the more complex diphospho-*myo*-inositol pentakisphosphates as, for example, 5-InsP_7_ (Fig. 1)^20^.

Here we report the design, step-economical synthesis, photophysical and metabolic evaluation of photocaged 5-InsP_7,_ and significantly, a general solution to the delivery of unmodified polyphosphate probes into cells using guanidinium-rich molecular transporters^21^. On cytoplasmic uncaging, 5-InsP_7_-mediated PH-domain translocation from the membrane into the cytosol in living cells is demonstrated for the first time on a 15-min timescale.

## Results

### Synthesis

**Figure 2:**
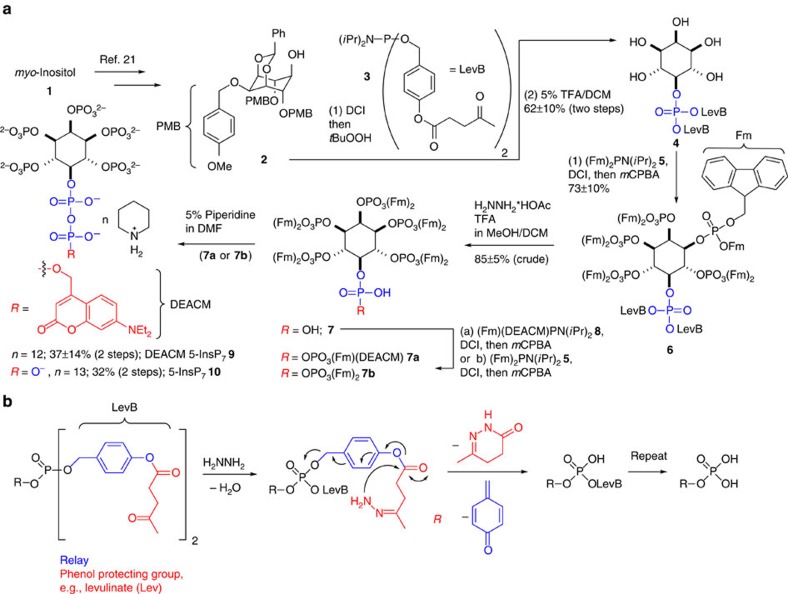
Synthesis of photocaged 5-InsP_7_ and mechanism of LevB cleavage. (**a**) Synthesis of DEACM 5–InsP_7_ **9** and 5-InsP_7_ **10** based on fluorenylmethyl (Fm) protection and a novel phosphate-protecting group (LevB). DCI, 4,5-dicyanoimidazole; DEACM, [7-(diethylamino)-coumarin-4-yl]methyl; Fm, fluorenylmethyl; *m*CPBA, metachloro perbenzoic acid; TFA, trifluoroacetic acid. (**b**) An adaptor strategy for phosphate release: hydrazine triggers levulinate (red) cleavage and 1,6-elimination (blue) to release free phosphate. Generally, levulinate could also be replaced with other phenol protecting groups.

All current synthetic approaches to access any InsP_7_ isomer rely on a global hydrogenation in the last step, during which up to 13 protecting groups need to be removed^22^,^23^,^24^,^25^,^26^,^27^,^28^,^29^,^30^. Significantly, however, hydrogenation is incompatible with photocaging groups and many other functional moieties like, for example, fluorophores. To address this problem, a novel strategy based on the development of a levulinate benzyl ester adaptor (LevB) is described. The introduction of this new protecting group and its combination with fluorenylmethyl (Fm) protection^31^,^32^,^33^ and photocage introduction enables the previously inaccessible synthesis of the first photoactivatable diphospho-inositol InsP_7_ probe **9** equipped with a [7-(diethylamino)-coumarin-4-yl]methyl (DEACM) photocage (Fig. 2a). It is noteworthy that this strategy potentially facilitates the introduction of other tags, such as, for example, photoaffinity labels and fluorophores.

The synthesis commenced with benzylidene protected **2** prepared as previously described (Fig. 2a)^23^. The 5-OH position of **2** is available for phosphitylation, allowing virtually any protected phosphate to be introduced. However, none of the existing protecting groups are compatible with the subsequent introduction of the coumarin cage. Such protecting groups would need to be stable under acidic and basic conditions and must enable double deprotection under very mild conditions. To meet these stringent requirements, a new phosphate-protecting group is required. The approach described herein is based on an Umpolung strategy that had been exploited in prodrug design for nucleotides^34^,^35^. Conceptually, this strategy is useful to generally couple phenol or alcohol protecting groups to phosphates via a benzyl adaptor, greatly enhancing the available protecting group strategies for phosphates (Fig. 2b). A novel P-amidite **3** was developed (Fig. 2, Supplementary Fig. 1), which is connected via a benzyl ester to a levulinate group (LevB). After oxidation to the phosphate triester **6**, the LevB group can be cleaved by hydrazone formation, initiating cyclization and finally a Grob-type fragmentation to give the unprotected phosphates (Fig. 2b).

After coupling P-amidite **3** to alcohol **2** and oxidation, all inositol-protecting groups were cleaved with TFA. The resultant protected monophosphate **4** was phosphitylated with (Fm)_2_-P-amidite **5** and oxidized. Notwithstanding the significant molecular crowding of hexakisphosphate **6**, both LevB groups were efficiently cleaved under mild conditions (hydrazine*AcOH/TFA), releasing phosphate **7**. Next, the P-anhydride was formed using Fm-protected photocaged P-amidite **8** (ref. 33). All 11 Fm-protecting groups were then cleaved with piperidine resulting in highly pure photocaged 5-InsP_7_ **9** in 45% yield over 2 steps (11% overall yield from **1**) on precipitation of the compound as the dodeca-piperidinium salt. The piperidinium ions can also be exchanged with sodium ions by precipitation. In addition, natural 5-InsP_7_ **10** can be prepared following the same strategy by using (Fm)_2_-P-amidite **5** in the anhydride forming reaction (8% overall yield from **1**)^36^,^37^. This eight-step synthesis represents a general strategy to access 5-InsP_7_ **10** and caged analogues in scalable amounts (30 mg of **9** have been prepared) and very high quality without the need for a final hydrogenation under aqueous conditions.

### *In vitro* stability and photophysical properties

**Figure 3:**
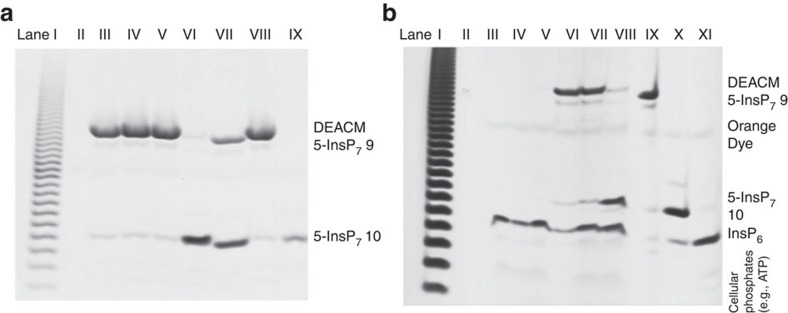
*In vitro* and *in cellulo* release of 5-InsP_7_. (**a**) Analysis of DEACM 5–InsP_7_ **9** by gel electrophoresis (PAGE) and toluidine blue staining. **9** is stable for hours in rat brain extract (lanes III–V) and can be uncaged by ultraviolet irradiation (lane VI). Lane I: poly-P marker. Lane II: empty. Lane III: **9** in brain extract (3 h). Lane IV: **9** in brain extract (2 h). Lane V: **9** in brain extract (1 h). Lane VI: **9** in brain extract (1 h), then ultraviolet irradiation (15 min). Lane VII: **9** in distilled water, then ultraviolet irradiation (15 min). Lane VIII: **9**. Lane IX: 5-InsP_7_ **10**. (**b**) Analysis of cellular uptake and *in cellulo* photouncaging with and without MoTr **11** after TiO_2_ microsphere extraction followed by gel electrophoresis (PAGE). Bands containing **9** and **10** were additionally extracted and analysed by MALDI mass spectrometry. **9** only enters cells in the presence of MoTr **11** (lanes VI, VII) and can be uncaged in living cells (lane VIII). Lane I: Poly-P marker to assess quality of separation. Lane II: empty. Lane III: HeLa cells (control). Lane IV: HeLa cells+**11** (control). Lane V: HeLa cells+**9** (5 h). Lane VI: HeLa cells+**9**+**11** (5 h), Lane VII: HeLa cells+**9**+**11** (16 h). Lane VIII: HeLa cells+**9**+**11** (16 h, then 10 min irradiation 366 nm, 4W). Lane IX: DEACM 5–InsP_7_ **9** (control). Lane X: 5-InsP_7_ **10** (control). Lane XI: InsP_6_ (control).

To serve as a useful tool, DEACM 5–InsP_7_ **9** must be stable towards enzymatic digestion to enable cellular uptake and release only on photolysis. To test its stability, **9** was incubated in tissue homogenate (brain, Fig. 3a; liver, Supplementary Fig. 3 and Supplementary Methods) and cell extract (Supplementary Figs 4–5 and Supplementary Methods). Readout was achieved by resolution on polyacrylamide gels (35%, Fig. 3 and Supplementary Methods)^38^. DEACM 5–InsP_7_ **9** did not decompose under these conditions over incubation times up to 5 h (Fig. 3a, Lanes III–V and Supplementary Figs 3–5). Thus, **9** is a probe that has the potential to be broadly applied in different cell and tissue types. Importantly, on exposure to ultraviolet light (366 nm, 4 W, distance 10 cm) in extracts, it was cleanly converted into 5-InsP_7_ **10**, as verified by PAGE (Fig. 3a, Lanes VI and VII and Supplementary Figs 3–4) and HPLC analysis (Supplementary Fig. 6) with **10** as a standard.

Next, the photophysical properties of DEACM 5–InsP_7_ **9** were characterized. The quantum yield for the disappearance of **9** Δ*ϕ*_*chem*_ is 0.71% at 355 nm as determined by actinometry following a novel protocol (Supplementary Methods)^39^,^40^,^41^. The fluorescence quantum yield *ϕ*_f_ is 6.2% and the lifetime *τ*_f_ is 1.2 ns. Notably, **9** also exhibits typical coumarin fluorescence at 500 nm (excitation at 386 nm; Supplementary Figs 7–10 and Supplementary Methods)^33^,^42^.

### Cellular delivery and uncaging

**Figure 4:**
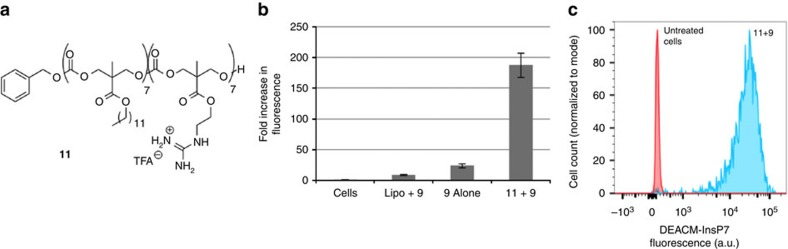
Intracellular delivery of photocaged 5-InsP_7_ to HeLa cells with a guanidinium-rich transporter. (**a**) Structure of amphipathic oligocarbonate transporter, **11**. (**b**) Cellular uptake of **9** as determined by flow cytometry. Complexes were formulated at a 1:1 mole ratio of **9** (5 μM) to **11**. Values reported are normalized to the autofluorescence of untreated cells. (**c**) Histogram plot of intracellular fluorescence demonstrates >99% delivery efficiency to cells.

Notwithstanding the efficiency of this synthesis, it was found as expected that DEACM 5–InsP_7_ **9**, like other polyanions^11^,^14^, does not readily cross the non-polar membrane of a cell (Fig. 4b,c). To address this problem, its non-covalent complexation, cell uptake and release using guanidinium-rich molecular transporters were studied^43^. **9** was mixed with amphipathic, guanidinium-rich transporter **11** (Fig. 4a), in an equimolar ratio to form nanoparticles. HeLa cells were treated with these complexes and analysed for coumarin fluorescence by flow cytometry. Significantly, while DEACM 5–InsP_7_ **9** itself does not appreciably enter cells, the transporter-complexed **9** does, as demonstrated by a 10-fold increase in intracellular fluorescence (Fig. 4b). According to flow cytometry analysis, over 99% of cells display increased levels of **9** following treatment with the transporter/DEACM–InsP_7_ complex (Fig. 4c). It is important to note that other transfection reagents like Lipofectamine 2000 do not efficiently deliver **9** into cells (Fig. 4b).

**Figure 5:**
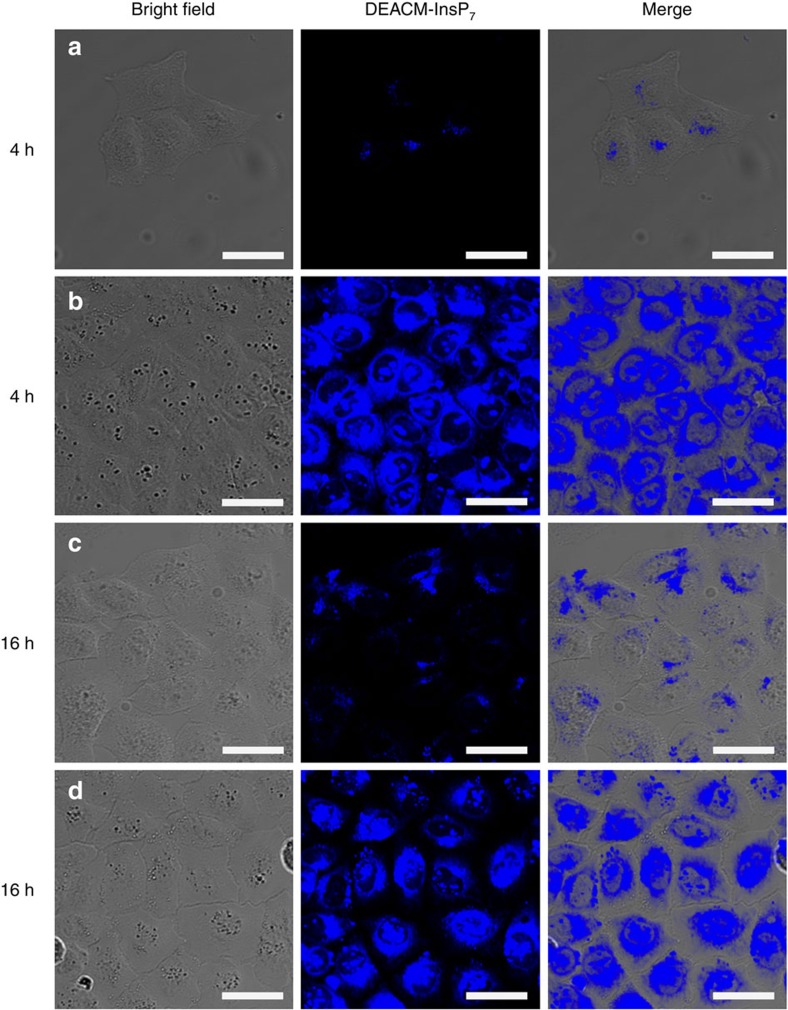
The molecular transporter 11 delivers photocaged 5-InsP_7_9 into the cytoplasm. Confocal microscopy analysis of HeLa cells incubated with 5 μM DEACM–InsP_7_ **9** in the presence or absence of transporter **11** after 4 and 16 h of incubation. Efficient uptake of **9** is demonstrated by blue coumarin fluorescence emitted from the compound. Cells treated with (**a**) **9** alone for 4 h, (**b**) **9**+**11** for 4 h, (**c**) **9** alone for 16 h and (**d**) **9**+**11** for 16 h. Scale bars, 35 μm.

Delivery and intracellular distribution of **9** were further analysed in HeLa cells by confocal microscopy after 4 and 16 h (Fig. 5). The z-stack analysis shows DEACM 5–InsP_7_ **9** distributed throughout the cytoplasm at both time points (Fig. 5, single z-slice shown). Both diffuse fluorescence and fluorescent puncta are observed, consistent with mixed diffusion or endosomal uptake and release^44^,^45^,^46^. This is additionally supported by a 65% reduction in cellular uptake when cells were treated at 4 °C, a condition known to inhibit most endocytotic processes (Supplementary Fig. 11).

Cellular uptake, stability and efficient uncaging in living cells was additionally verified by extraction of diphospho-inositol polyphosphates and other cellular phosphates based on a recently published TiO_2_ microsphere enrichment method^47^. Here it is shown that this method can also be used to extract analogues such as **9** from complex cell and tissue lysates (Fig. 3b and Supplementary Methods) enabling studies concerning its intracellular fate after delivery. After incubation of DEACM 5–InsP_7_ **9** with HeLa cells in the presence or absence of MoTr **11** and repeated washings to remove external **9**, the extracts prepared from those cells (1 million cells) clearly showed a distinct novel band corresponding to **9** in the PAGE analysis after enrichment with TiO_2_ and elution (Fig. 3b, lanes VI–VII), whereas no such uptake could be detected in the control experiment without transporter (Fig. 3b, lane V). To verify its identity, the band corresponding to DEACM 5–InsP_7_ **9** was extracted from the gel and analysed by MALDI mass spectrometry, demonstrating its intracellular stability (Supplementary Fig. 12 and Supplementary Methods) for multiple hours. Moreover, efficient intracellular uncaging by irradiation at 366 nm was proven using the same extraction and resolution method (TiO_2_ enrichment, then PAGE) in combination with mass spectrometry after extraction of Lane VIII (Fig. 3b). These conditions were found to be of no immediate toxicity (Supplementary Fig. 13 and Supplementary Methods). In summary, the photocaged molecule **9** is efficiently taken up by cells in the presence of MoTr **11**, evenly distributed throughout the cytoplasm, stable for multiple hours in its caged form and can be selectively uncaged to 5-InsP_7_ **10**, thus fulfilling the stringent requirements imposed on an intracellular signalling probe.

### PH-domain translocation

**Figure 6:**
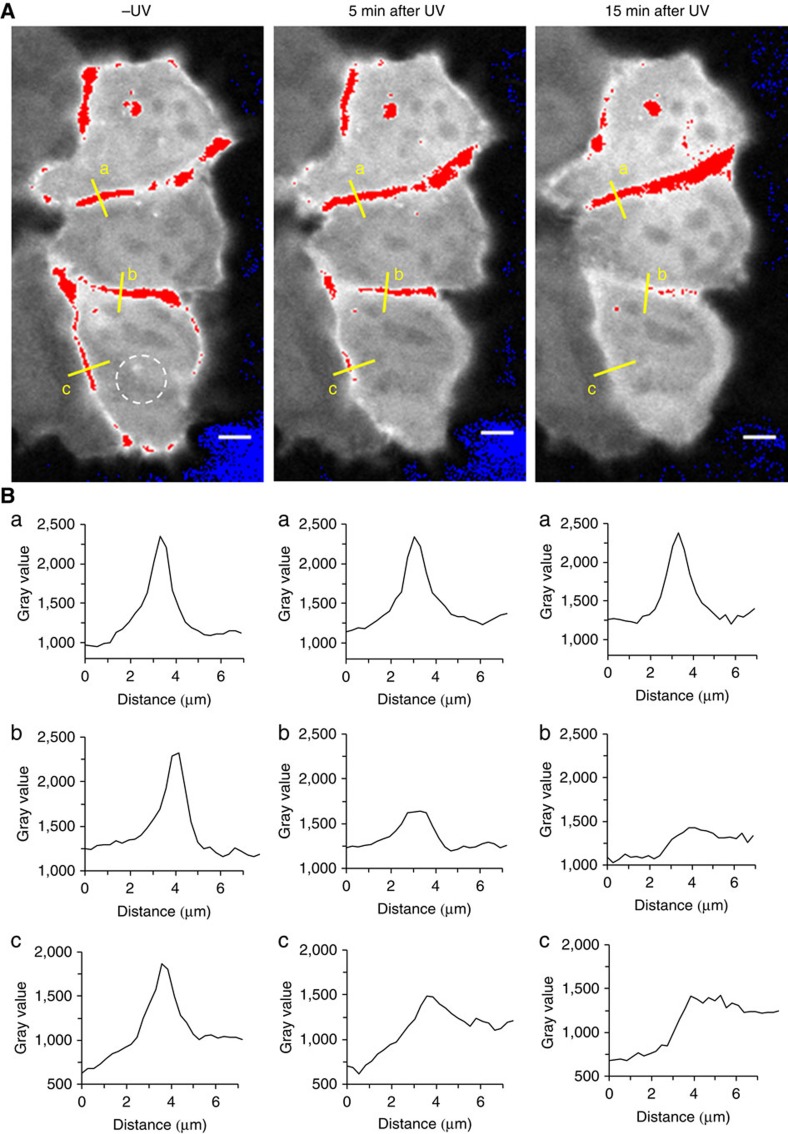
PH-domain translocation in irradiated and control cells. Confocal fluorescence microscopy analysis of PH–eGFP translocation in HeLa Kyoto cells after photouncaging in defined areas (dotted circle). (**A**) Serum-starved cells were loaded with 5 μM **9**+**11** for 4 h and then stimulated with IGF and EGF (100 ng ml^−1^). Robust recruitment of the PH-domain to the membrane is observed. Photouncaging in the dotted area (white circle) is achieved by short ultraviolet laser pulses and the change of fluorescence intensity followed over time (0, 5 and 15 min). (**B**) Development of the fluorescence intensity (indicated as gray value) over time (0, 5, 15 min) in three different membrane sections (a, b, c; distance in μm). Photouncaging leads to translocation of the PH–eGFP construct into the cytoplasm after 5 min from the membrane of the irradiated cell. After 15 min, complete translocation of the PH-domain into the cytoplasm is observed (**B**, b and c), whereas in the non-irradiated control cell the PH–eGFP construct remains localized on the membrane (**B**, a). Images are presented in pseudo-colour, normalized over time. Intensities were acquired pre-saturated, with the entire dynamic range of intensity available. Scale bars, 5 μM.

To determine the suitability of the combined delivery and uncaging strategy for a deeper understanding of the effect of InsP_7_ fluctuations, PH-domain translocation on cytoplasmic InsP_7_ release was studied. The rationale for this experiment is provided by the lean phenotype displayed by IP6K1 knockout mice on high-fat diet and the observation that InsP_7_ inhibit Akt phosphorylation *in vitro* and *in vivo* by binding to the PH-domain^9^. Collectively, these findings suggest an effect of 5-InsP_7_ on membrane localization of Akt. However, no tool to augment any InsP_7_ within seconds in living cells was previously available. With the new tools in hand, HeLa cells were transiently transfected with a plasmid expressing the PH-domain of Akt fused to an enhanced green fluorescent reporter protein (eGFP)^48^,^49^. Cells were serum-starved to induce cytoplasmic localization of the PH-domain due to absence of growth factors and therefore inactivation of the PI3K/Akt/mTOR pathway^50^. PH–eGFP plasma membrane association was then efficiently induced within 10 min on external addition of a combination of growth factors (insulin-like growth factor (IGF); endothelial growth factor (EGF)) into the medium. During the starvation period, cells were loaded with caged InsP_7_ **9/**MoTr **11** nanoparticles for 4 h. This treatment alone had no effect on PH-domain localization. Next, cells were irradiated under a confocal laser-scanning microscope with short laser pulses (375 nm, 10 MHz, 30 s) in different areas (Fig. 6 dotted circle, and Supplementary Figs 14–23), and PH-domain localization was traced using the green channel. After photouncaging, a delayed but complete PH-domain translocation from the plasma membrane into the cytoplasm was observed, and these results were repeated several times (*n*=4). Significantly, translocation did not occur when cells were incubated with photocaged InsP_7_ **9** or MoTr **11** only (Supplementary Figs 18–23). In these cases, the PH-domains remained localized on the membrane for several hours, demonstrating the need for the presence of all components and ruling out photobleaching of eGFP in the irradiated areas. A detailed analysis of additional micrographs in pseudo-colour with ratiometric changes is shown in the Supplementary Information (Supplementary Figs 14–23). This is the first example of controlled 5-InsP_7_ **10** augmentation inside of a living cell within a few seconds timeframe coupled to a microscopic readout on the single cell level. We posit that this strategy will be useful to understand InsP_7_ signalling in more detail as previously possible, as evidenced by the delayed PH-domain translocation observed for the first time in our experiments.

## Discussion

This study provides a new strategy to synthesize InsP_7_ that enables introduction of caging subunits. The potential utility of photocaged 5-InsP_7_ **9** was demonstrated by photon-triggered uncaging in rat brain homogenate and other cell extracts. A complex of **9** with molecular transporter **11** was then shown to efficiently enter cells after non-covalent nanoparticle assembly. Collectively, these results provide the first example of the synthesis of a photocaged analogue of InsP_7_ and of its subsequent delivery into cells using non-covalent complexation with a guanidinium-rich molecular transporter. A recently developed TiO_2_ microsphere enrichment method was applied to study the *in cellulo* stability of 5-InsP_7_ analogues and their efficient photochemical release in combination with MALDI mass spectrometry. We expect that this combined synthesis, delivery and analytical strategy will find widespread and general application in cell signalling studies as a convenient way to rapidly augment 5-InsP_7_ **10** with spatiotemporal resolution. Along these lines, it was shown that cytoplasmic release of 5-InsP_7_ triggers delayed but complete membrane desorption of the PH-domain of Akt within 15 min. In the human proteome, PH-domains are the 11th most common domain^51^, and the new approach described in this publication will enable a systematic understanding of the effect of inositol-pyrophosphate augmentation on protein localization.

## Methods

### Experimental data of synthetic compounds

For ^1^H, ^13^C and ^31^P NMR spectra of compounds and MALDI and HR-ESI MS spectra see Supplementary Figs 24–70. ^1^H NMR spectra were recorded on Bruker 400 MHz spectrometers or Bruker 500 MHz spectrometers (equipped with a cryo platform) at 298 K in the indicated deuterated solvent. ^31^P[^1^H]-NMR spectra and ^31^P NMR spectra were recorded with ^1^H-decoupling or ^1^H coupling on Bruker 162 MHz or Bruker 202 MHz spectrometers (equipped with a cryo platform) at 298 K in the indicated deuterated solvent. All signals were referenced to an internal standard (PPP). ^13^C[^1^H]-NMR spectra were recorded with ^1^H-decoupling on Bruker 101 MHz or Bruker 125 MHz spectrometers (equipped with a cryo platform) at 298 K in the indicated deuterated solvent. All signals were referenced to the internal solvent signal as standard (CDCl_3_, δ 77.0; CD_3_OD, δ 49.0; DMSO-d_6_, δ 39.5).

Detailed synthetic procedures for all new compounds are provided in the Supplementary Information (see Supplementary Methods, chemical synthesis).

### Cellular uptake by flow cytometry

Caged-IP7 **9** and oligomer **11** were brought up in pH 7.4 PBS buffer at 1 mM concentrations. HeLa cells were seeded at 40,000 cells per well in a 24-well plate and allowed to adhere overnight. The IP7:co-oligomer complexes were formed at a 1:1 molar ratio by mixing 8 μl of 1 mM oligomer stock with 8 μl of 1 mM caged-IP7 **9** stock in 184 μl PBS pH 7.4. For conditions with caged-IP7 **9** alone, 8 μl of 1 mM caged-IP7 **9** stock was added to 192 μl PBS pH 7.4. The complexes were allowed to incubate for 30 min at room temperature. The Lipofectamine 2000 control was prepared in OptiMEM according to the manufacturer's instructions (0.75 μl Lipofectamine into 62.5 μl. OptiMEM, 8 μl caged-IP7 **9** stock into 54.5 μl OptiMEM). The cells were washed with ∼0.5 ml serum-free DMEM medium, then 400 μl serum-free DMEM was added to wells with untreated cells; 368.75 μl to wells treated with Lipofectamine 2000; 350 μl to treated wells. Then 31.25 μl Lipofectamine:caged-IP7 **9** and 50 μl of the caged-IP7:co-oligomer complexes were added to each respective well for a final caged-IP7 **9** concentration of 5 μM; all conditions were performed in triplicate. The cells were incubated at 37 °C for 3 h. The medium was then removed and the cells were washed with 1.0 ml PBS. About 0.4 ml EDTA trypsin was added, and the cells incubated for 8 min at 37 °C. Next, 0.6 ml of serum-containing DMEM medium was added, and the contents of each well were transferred to a 15 ml centrifuge tube and centrifuged (1,200 r.p.m. for 5 min). The cells were collected and re-dispersed in 200 μl PBS, transferred to FACS tubes, and read on a flow cytometry analyser. Results were analysed using FlowJo software. The data presented are the mean fluorescent signals from 10,000 cells analysed.

### Fluorescence microscopy

Confocal microscopy was conducted on a CLSM SP5 Mid ultraviolet–visible Leica inverted confocal laser-scanning microscope equipped with a 15 W MaiTai DeepSee twophoton laser (Stanford Cell Sciences Imaging Facility, Award #S10RR02557401 from the National Center for Research Resources). HeLa cells were incubated in serum-free DMEM with 5 μM **9** or **9**+**11** (equimolar), for 4 or 16 h. After incubation, media was removed and cells washed 3 × with 1.0 mg ml^−1^ heparin (180 U mg^−1^, Aldrich) solution in PBS, then imaged in clear serum-free DMEM. Pictures were recorded with a HCX APO L × 20/1.00 water immersion objective and photomultiplier tube detector.

### *eGFP–Akt-PH-*domain translocation

Mammalian cells (HeLa Kyoto) were grown up to 50–60% confluence (eight wells Lab-Tek Chamber Slide) in 250 μl DMEM (10% FBS, 10% Pen/Strep, high glucose (4.5 g l^−1^)) at 37 °C, 5% CO_2_ for 16 h. Cells were then washed with 250 μl PBS followed by a wash with serum-free DMEM (-FBS, -Pen/Step). About 230 μl serum-free DMEM medium were then added to each well.

Transfection of HELA cells with the eGFP Akt-PH-domain for imaging: The transfection mixture was prepared for eight wells. About 200 ng DNA (eGFP–Akt-PH) in 150 μl DMEM (-FBS) were incubated for 10 min at room temperature. Then, 10 μl FuGENE transfecting reagent were added to DNA containing DMEM (-FBS) medium and incubated for 20 min at room temperature. About 20 μl of the transfection mixture were then added to each well. Cells were incubated for 6–8 h at 37 °C (5% CO_2_.). After this period, the transfection mixture was removed by washing the cells with 250 μl PBS and DMEM (-FBS). Cells were starved by adding 250 μl DMEM (-FBS) and incubated for 14–16 h at 37 °C (5% CO_2_).

After overnight starvation, the cells were washed with PBS (250 μl) and 230 μl DMEM (-FBS) were added. Afterwards, 20 μl sample mixture (consisting of caged InsP_7_:Co-oligomer (D:G7:7) complexes) were added to each well and distributed equally. The final concentration of the complex was 5 μM for each well. Cells were then incubated at 37 °C (5% CO_2_) for 4 h.

### Imaging of Akt-PH translocation

After 4-h incubation with 9 and 11, HeLa cells were washed with 250 μl (2 ×) imaging buffer (115 mM NaCl, 1.2 mM CaCl_2_, 1.2 mM MgCl_2_, 1.2 mM K_2_HPO_4_, 20 mM HEPES). About 200 μl imaging buffer were added to the wells. Translocation of eGFP–Akt-PH to the membrane was stimulated by adding a mixture of growth factors EGF and IGF (100 ng ml^−1^ each). Translocation was monitored over 10 min at 37 °C (5% CO_2_).

Cells that had responded to treatment with the growth factors and that had the PH-domain localized at the membrane were used in the uncaging experiments by confocal laser-scanning microscopy. Non-illuminated neighboring cells were used as controls.

### Confocal laser-scanning microscopy

Imaging was performed on an Olympus IX83 confocal laser-scanning microscope at 37 °C in a 5% CO_2_ high humidity atmosphere (EMBL incubation box). Imaging was performed using an Olympus Plan-APON × 60 (numerical aperture 1.4, oil) objective. The images were acquired utilizing a Hamamatsu C9100-50 EM CCD camera. Image acquisition was performed via FluoView imaging software, version 4.2. The green channel was imaged using the 488 nm laser line (120 mW cm^−2^) at 3% laser power and a 525/50 emission mirror. The red channel was imaged using the 559 nm laser (120 mW cm^−2^) at 2.0% laser power and a 643/50 emission filter. A pulsed 375 nm laser line (10 MHz) was applied for uncaging experiments. For uncaging experiments, circular regions of interest of 4–10 μm diameter were pre-defined. Pre-activation images were captured for five frames (5 s per frame), followed by 30 s of activation within the regions of interest. Recovery images were captured for 35 min at a frame rate of 5 s per frame.

### Image analysis

Image analysis was conducted utilizing Fiji open source image analysis software tool^52^. Lookup tables were applied to match the colour within the recorded image with the wavelengths of detected light. For comparability, the lookup tables of pre- and postactivated images were set the same weighting.

## Additional information

**How to cite this article**: Pavlovic, I. *et al*. Cellular delivery and photochemical release of a caged inositol-pyrophosphate induces PH-domain translocation *in cellulo*. *Nat. Commun.* 7:10622 doi: 10.1038/ncomms10622 (2016).

## Supplementary Material

Supplementary Information Supplementary Figures 1-70, Supplementary Methods and Supplementary References

## Figures and Tables

**Figure 1:**
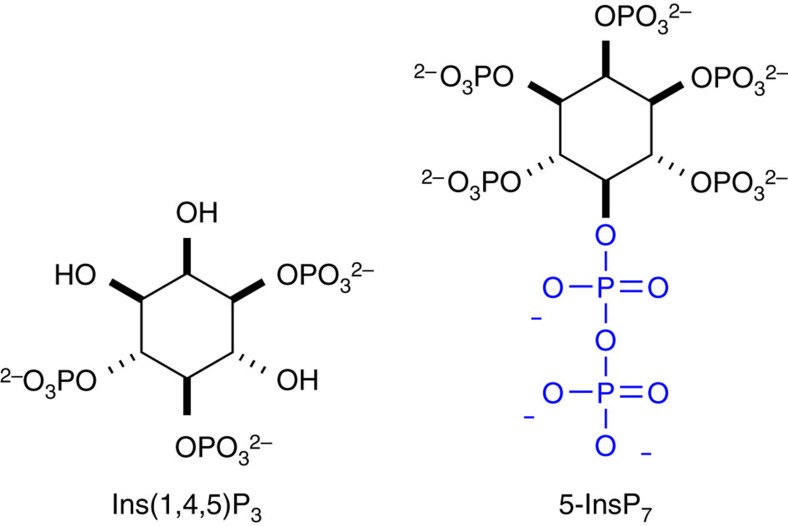
Phosphorylated second messengers derived from *myo*-inositol. Chemical structures of *myo*-Inositol 1,4,5-trisphosphate and 5-diphospho-*myo*-inositol pentakisphosphate (5-InsP_7_).
