# Supplementary material for: Cellular delivery and photochemical release of a caged inositol-pyrophosphate induces PH-domain translocation in cellulo
Source: Nat Commun. 2016 Feb 4;7:10622. doi: 10.1038/ncomms10622 (PMC4743007; doi:10.1038/ncomms10622)
Supplement: Supplementary Information — Supplementary Figures 1-70, Supplementary Methods and Supplementary References [file ncomms10622-s1.pdf]

Supplementary Figures

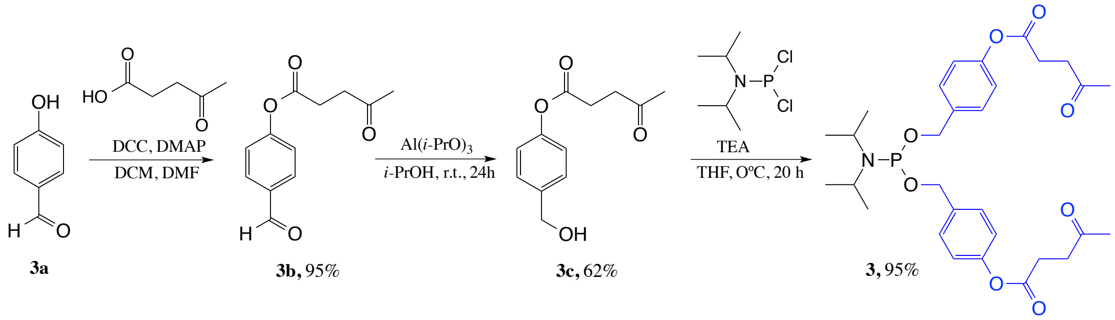

Supplementary Figure 1. Synthesis of LevB P-amidite 3. Analytical details are reported in the synthesis section.

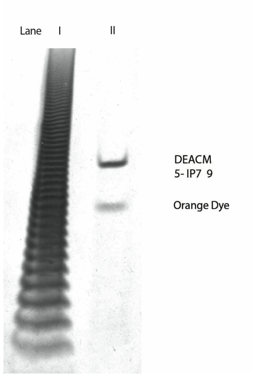

Supplementary Figure 2. DEACM 5-InsP$_7$ 9 purity assignment by PAGE. The purity of synthetic DEACM-InsP$_7$ **9** was analyzed by PAGE (35% Acrylamide- bis acrylamide) and staining with fluorescent dye (toludine blue) according to a published procedure.[1] Orange G dye has been added to track progression of PAGE. Lane I: Poly-P marker. Lane II: DEACM 5-InsP$_7$ **9** and orange dye (marker)

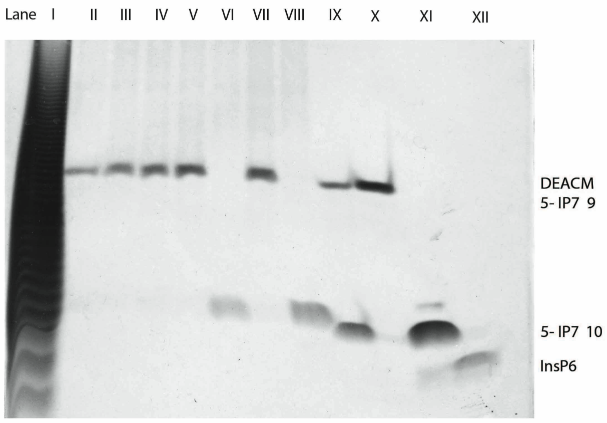

Supplementary Figure 3. DEACM 5-InsP$_7$ 9 is stable over multiple hours in rat liver tissue extract and can be uncaged by UV irradiation. Lane I: Poly-P marker. Lane II: DEACM 5-InsP$_7$ **9** in liver extract (5h). Lane III: DEACM 5-InsP$_7$ **9** in liver extract (4h). Lane IV: DEACM 5-InsP$_7$ **9** in liver extract (3h). Lane V: DEACM 5-InsP$_7$ **9** in liver extract (2h). Lane VI: DEACM 5-InsP$_7$ **9** in liver extract (2h), then UV irradiation (15 min). Lane VII: DEACM 5-InsP$_7$ **9** in liver extract (1h). Lane VIII: DEACM 5-InsP$_7$ **9** in liver extract (1h), then UV irradiation (15 min). Lane IX: synthetic standard DEACM 5-InsP$_7$ **9** UV irradiation (15 min) in distilled water. Lane X: synthetic standard DEACM 5-InsP$_7$ **9**. Lane XI: synthetic standard 5-InsP$_7$ **10**. Lane XII: commercial standard InsP$_6$.

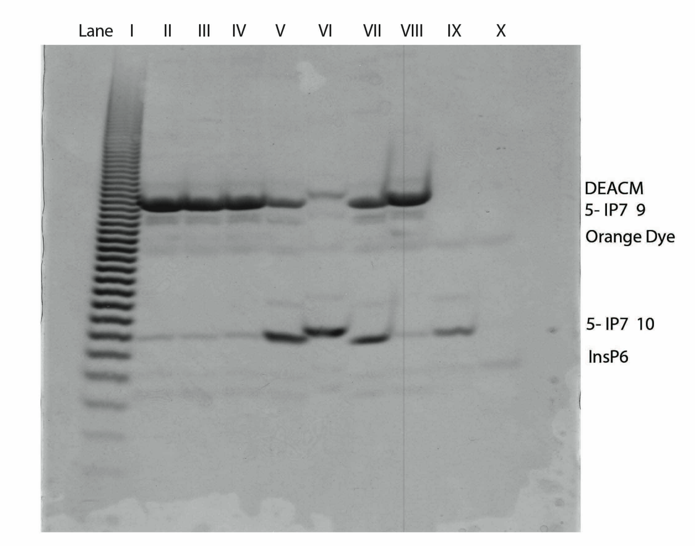

Supplementary Figure 4. DEACM 5-InsP$_7$ 9 is stable over multiple hours in MCF7 cell extract and can be uncaged. Lane I: Poly-P marker. Lane II: DEACM 5-InsP$_7$ **9** in MCF7 cell extract (3h). Lane III: DEACM 5-InsP$_7$ **9** in MCF7 cell extract (2h). Lane IV: DEACM 5-InsP$_7$ **9** in MCF7 cell extract (1h). Lane V: DEACM 5-InsP$_7$ **9** in MCF7 cell extract (1h), then UV irradiation (10 min). Lane VI: Synthetic standard DEACM 5-InsP$_7$ **9** in reaction buffer (without extracts), then UV irradiation (15 min). Lane VII: Synthetic standard DEACM 5-InsP$_7$ **9** in deionized water (without extracts), then UV irradiation (15 min). Lane VIII: Synthetic standard DEACM 5-InsP$_7$ **9**. Lane XI: synthetic standard 5-InsP$_7$ **10**. Lane X: commercial standard InsP$_6$.

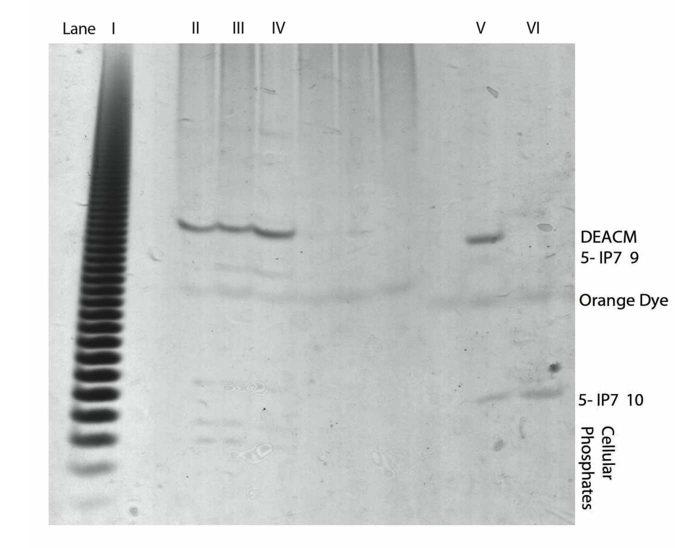

Supplementary Figure 5. DEACM 5-InsP$_7$ 9 is stable over multiple hours in HCT116 cell extract. Lane I: Poly-P marker. Lane II: DEACM 5-InsP$_7$ **9** in HCT116 cell extract (5h). Lane III: DEACM 5-InsP$_7$ **9** in HCT116 cell extract (3h). Lane IV: DEACM 5-InsP$_7$ **9** in HCT116 cell extract (1h). Lane V: synthetic standard DEACM 5-InsP$_7$ **9**. Lane VI: synthetic standard 5-InsP$_7$ **10**.

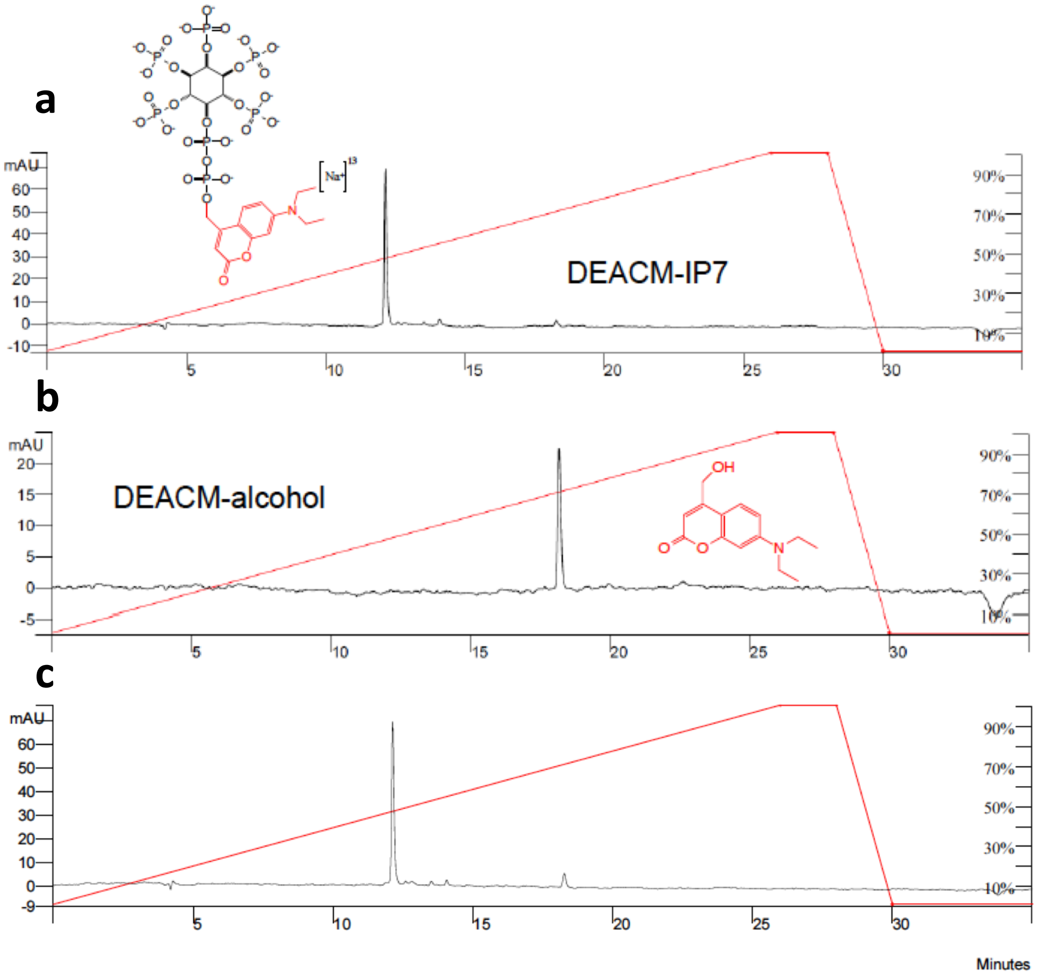

Supplementary Figure 6. HPLC analysis of DEACM-5-InsP$_7$ 9 photocleavage. HPLC analysis of **9** before (a) and after short irradiation (290 laser shots) with a Nd:YAG laser (c). Chromatogram (b) shows DEACM-OH as reference, which is released upon irradiation (c). With 290 laser shots, no complete uncaging was achieved.

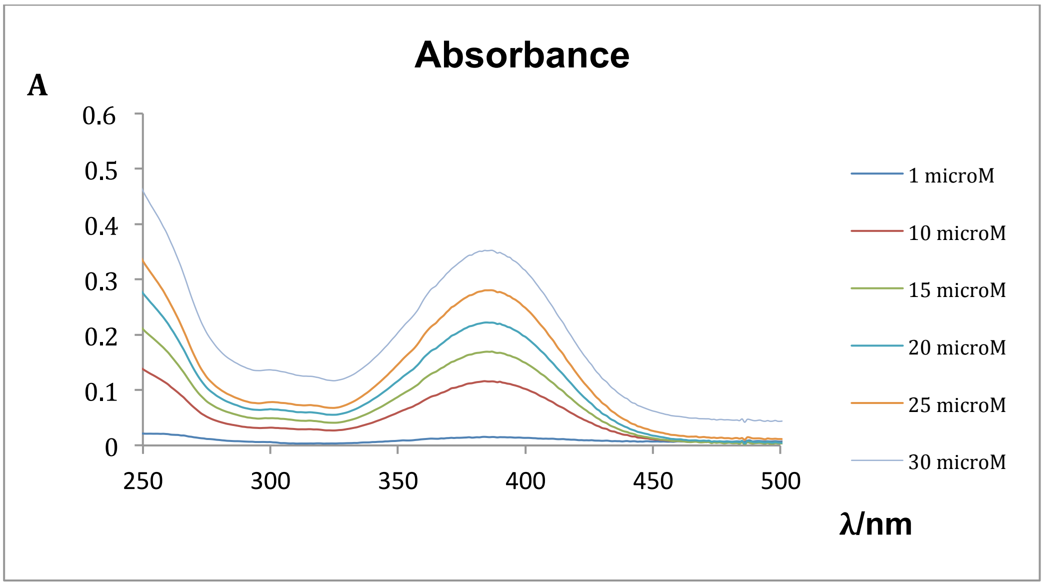

Supplementary Figure 7. Absorbance Spectra of DEACM 5-InsP$_7$. Absorbance spectra of **9** recorded at different concentrations,.

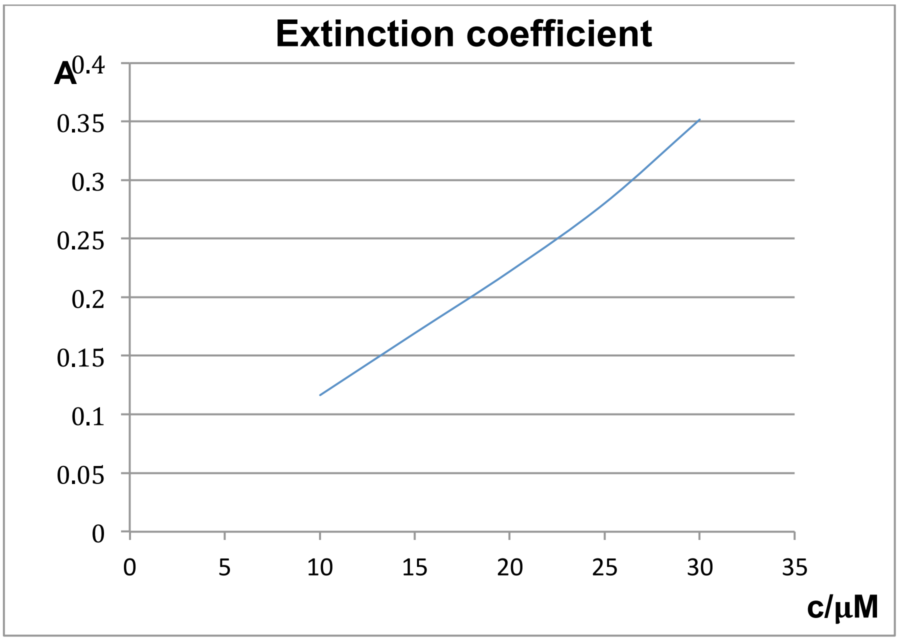

Supplementary Figure 8. Extinction coefficient. Determination of the extinction coefficient of **9**

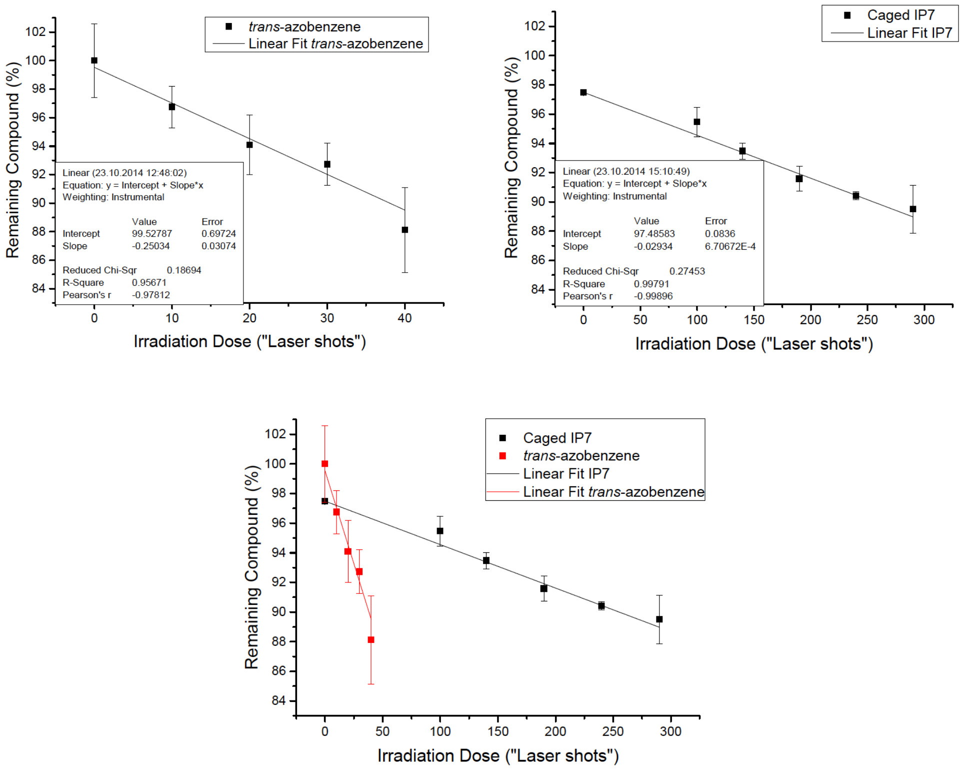

Supplementary Figure 9. Determination of the uncaging quantum yield of DEACM 5-InsP$_7$ using *trans*-azobenzene as a reference. The irradiation dose (laser shots at 355 nm) was plotted against the remaining compound **9** (determined by HPLC, see supplementary figure **6** for an example) and compared to isomerization of trans-azobenzene. The progress of the photoisomerization of azobenzene was monitored by UV/Vis absorbance spectroscopy. Quantum yields were obtained according to supplementary equation 1.

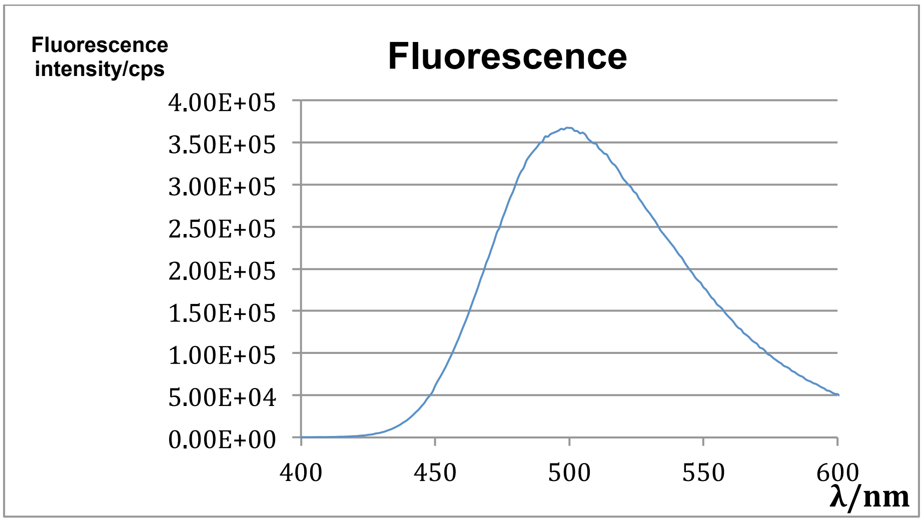

Supplementary Figure 10. Fluorescence Spectrum. Fluorescence emission of 9 after irradiation at 385 nm.

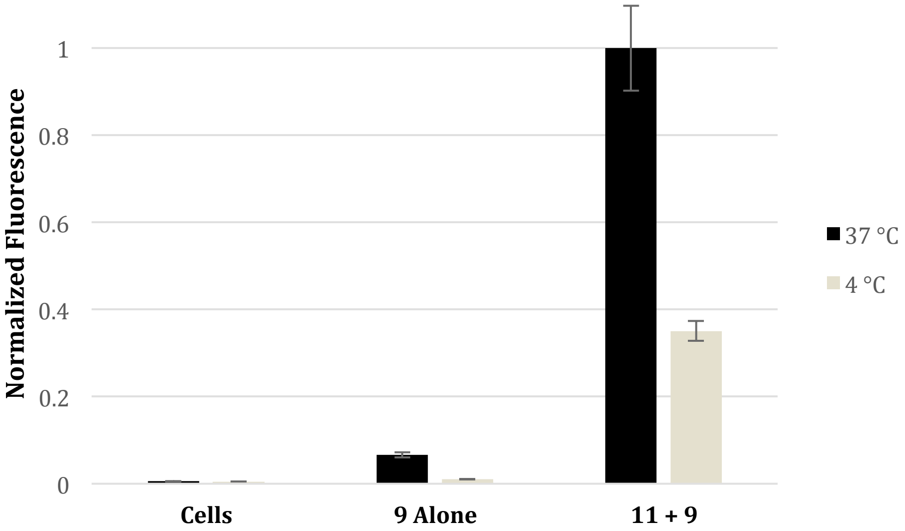

Supplementary Figure 11. Cellular Uptake at 37°C and 4 °C. Comparison of cellular uptake of **9** at 37 or 4 °C determined by flow cytometry analysis of coumarin fluorescence. All values are normalized to the fluorescence of **9** + **11** complexes treated at 37 °C. The 65% reduction in uptake for cells treated at 4 °C is indicative that endocytotic mechanisms play a dominant role.

MALDI-MS Report (Bruker Autoflex I)

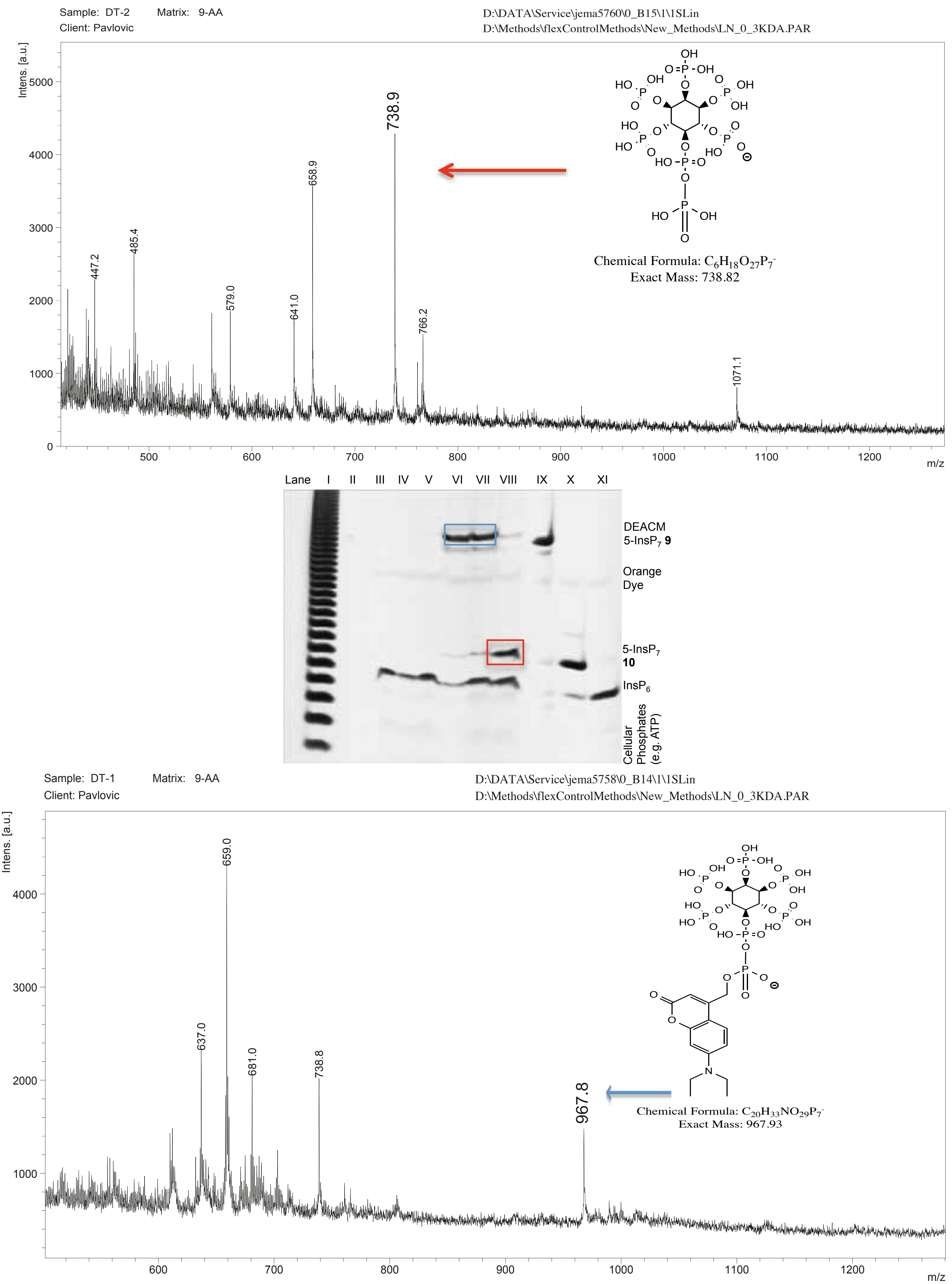

Supplementary Figure 12. Cellular Uptake and Stability. Extraction of bands from the Gel and MS analysis confirms the identity of **9** in living cells after 16h incubation (blue box, bottom MS) and its efficient uncaging after irradiation at 366 nm (4W, 10 min) in living cells (red box, top MS)

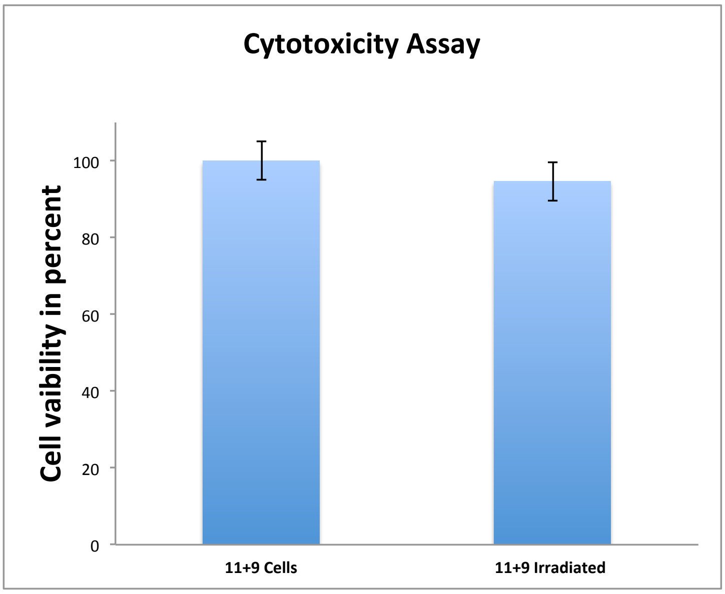

Supplementary Figure 13. Cytotoxicity Assay. Irradiation of cells (5min) at 350 nm in presence of **9+11** is not toxic to cells as determined by the Alamar Blue assay.

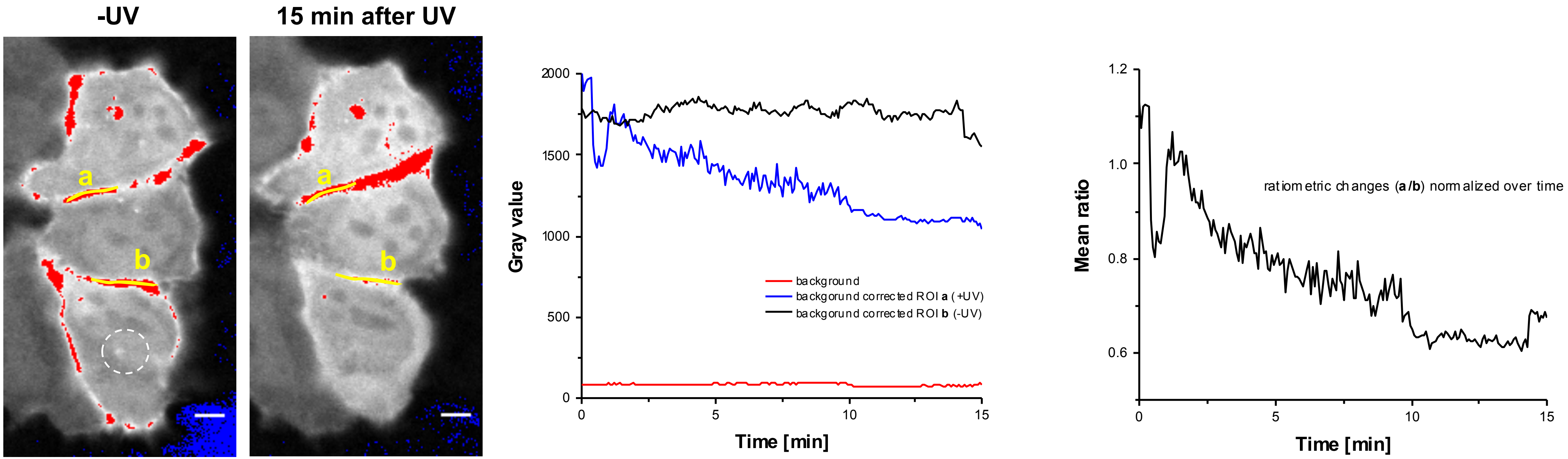

Supplementary Figure 14. Ratiometric changes normalized over observation time, after incubation with DEACM-InsP7 and MoTr and UV-mediated uncaging. Intensities were determined along the indicated membrane sections of the control cell (-UV, section **a**) and the cell that sensed the uncaging UV beam (+UV, dashed circle, section **b**). All recorded intensity signals of indicated sections are represented background-corrected. Ratiometric changes over the observation time were determined by normalizing the membrane signal (section **b**) to the signal of the internal control section **a** (-UV). Only the cell that sensed the uncaging UV beam exhibited decreasing membrane signal over time (section **b**). The membrane signal of the control cell (-UV, section **a**) remained constant over the observation time. Images are presented in pseudo-color, normalized over time. Intensities were acquired pre-saturated, with the entire dynamic range of intensity available. Scale bars: 5 µm.

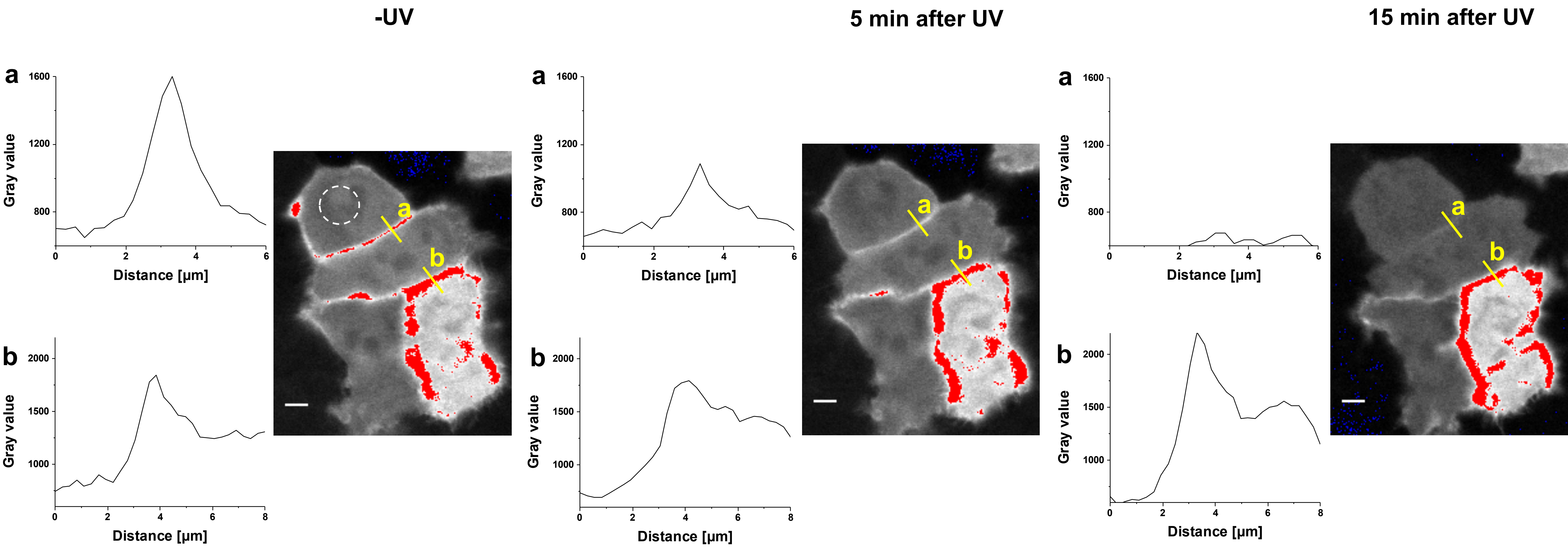

Supplementary Figure 15. Green channel recordings of GFP-Akt-PH after incubation with DEACM-InsP7 and MoTr and UV-mediated uncaging. Only the cell that sensed the uncaging UV beam exhibited decreasing membrane signal over observation time (section **a**, dashed circle). While the membrane signal of the illuminated cell (section **a**) decreased, the membrane signal of the control cell (-UV, section **b**) even further increased over observation time – presumably due to the ongoing (highly dynamic) dynamic accumulation of GFP-Akt-PH at the membrane. The sharp intensity peak in section **a** decreased. Intensities were acquired pre-saturated, with the entire dynamic range of intensity available. Scale bars: 5 µm.

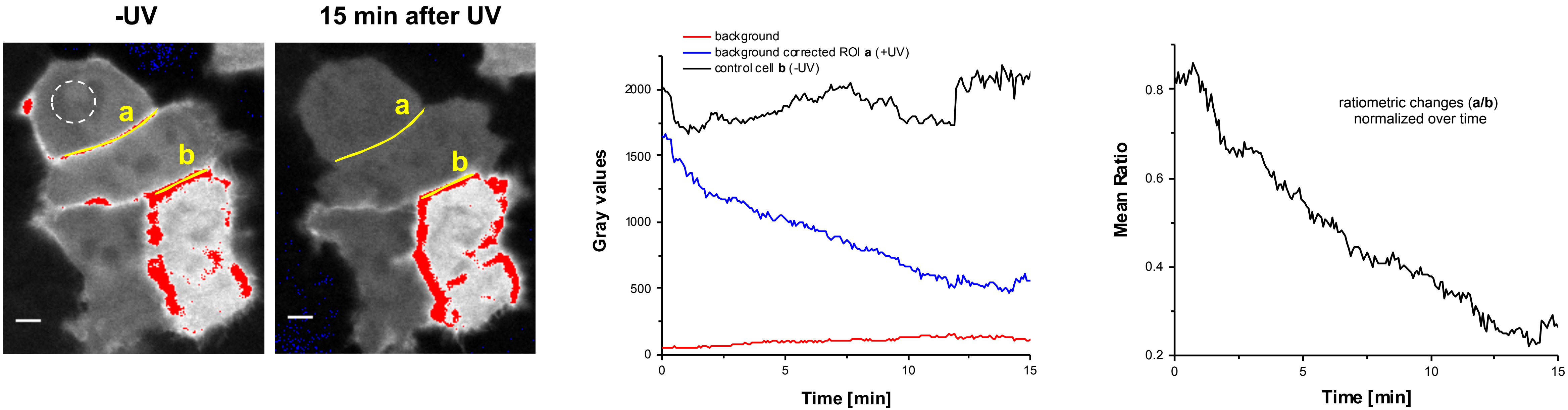

Supplementary Figure 16. Ratiometric changes that were normalized over observation time, following incubation with DEACM-InsP7 and MoTr and UV-mediated uncaging. Intensities were determined along the indicated membrane sections of the control cell (-UV, section **b**) and the cell that sensed the uncaging UV beam (dashed circle, section **a**). All intensity signals are represented background-corrected. Ratiometric changes over the observation time were determined by normalizing the membrane signal **a** to the signal of the control section **b.** Only the cell that sensed the uncaging UV beam (dashed circle) exhibited decreasing membrane signal over time (section **a**). The membrane signal of the control cell (-UV, section **b**) remained almost constant over the observation time. Images are presented in pseudo-color, normalized over time. Scale bars: 5 µm.

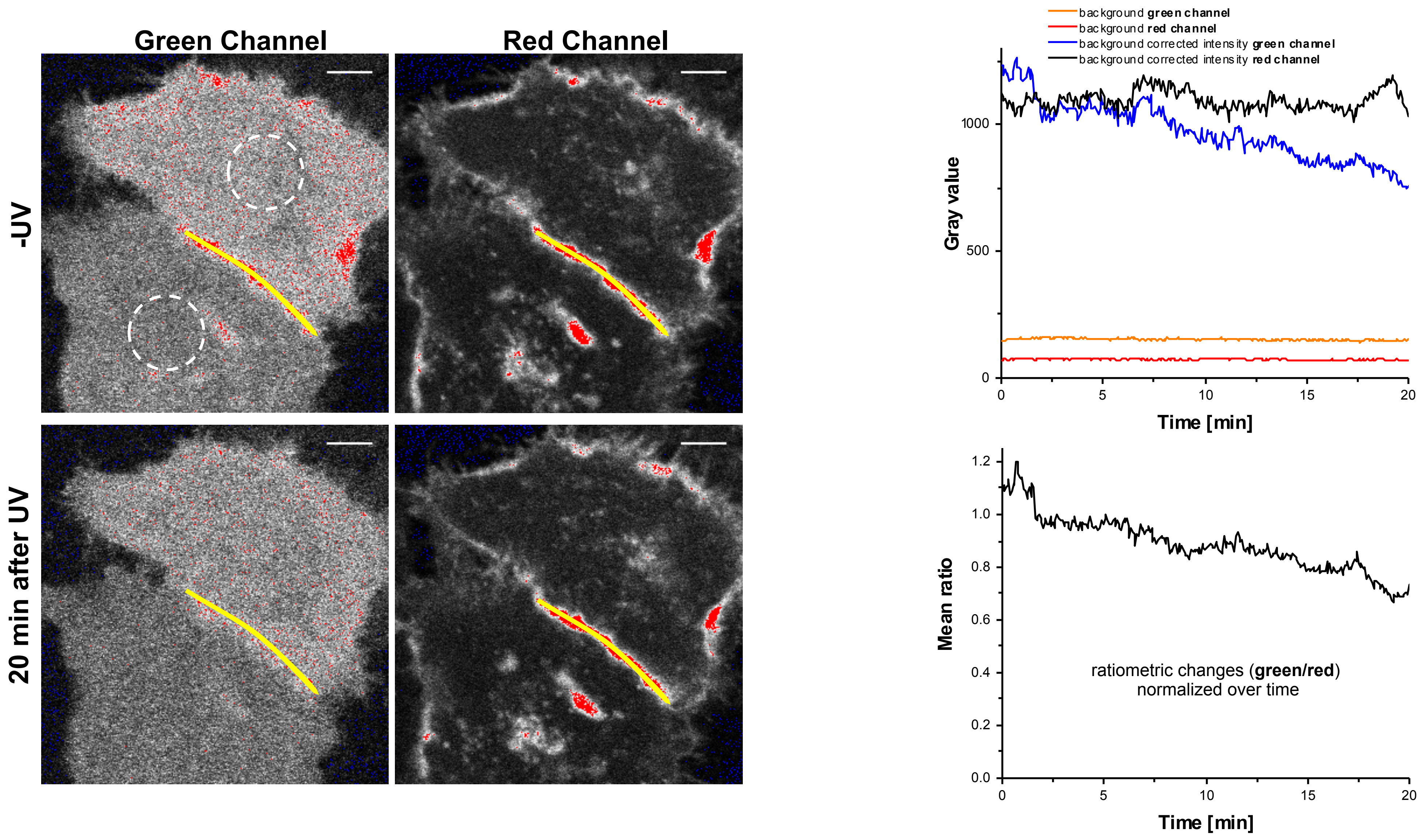

Supplementary Figure 17. Green and red channel recordings after co-overexpression of GFP-Akt-PH and RFP-GPI, incubation with DEACM-InsP7 and MoTr and UV-mediated uncaging. Following the uncaging UV-beam (dashed circles) the membrane signal exclusively decreased in the green fluorescence channel over the observation time. The signal of the GPI-membrane anchor in the red control channel remained constant. Ratiometric changes over the observation time were determined by normalizing the membrane signal in the green fluorescent channel to the signal of the red channel. Intensity signals are represented background corrected. Images are presented in pseudo-color, normalized over time. Intensities were acquired pre-saturated, with the entire dynamic range of intensity available. Scale bars: 5 µm.

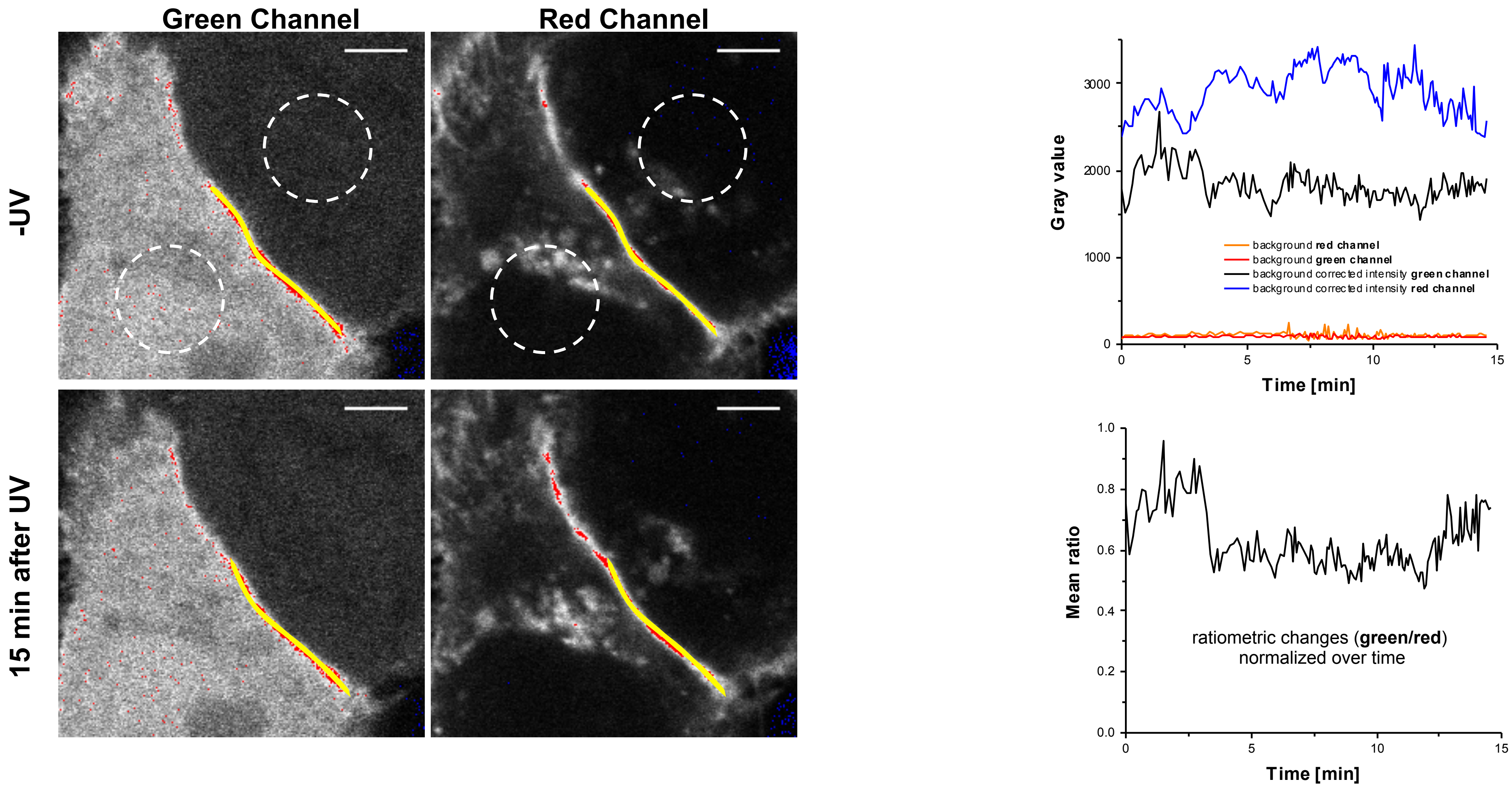

Supplementary Figure 18. Green and red channel recordings after co-overexpression of GFP-Akt-PH and RFP-GPI, incubation with DEACM-InsP7 (-MoTr) and UV-mediated uncaging. Following the uncaging UV-beam (dashed circles) the membrane signals of indicated regions remained at constant levels in the red and green fluorescent channels over observation time. Intensity signals are represented background corrected. Ratiometric changes over the observation time were determined by normalizing the membrane signal in the green fluorescent channel to the signal of the red channel. Images are presented in pseudo-color, normalized over time. Intensities were acquired pre-saturated, with the entire dynamic range of intensity available. Scale bars: 5 µm.

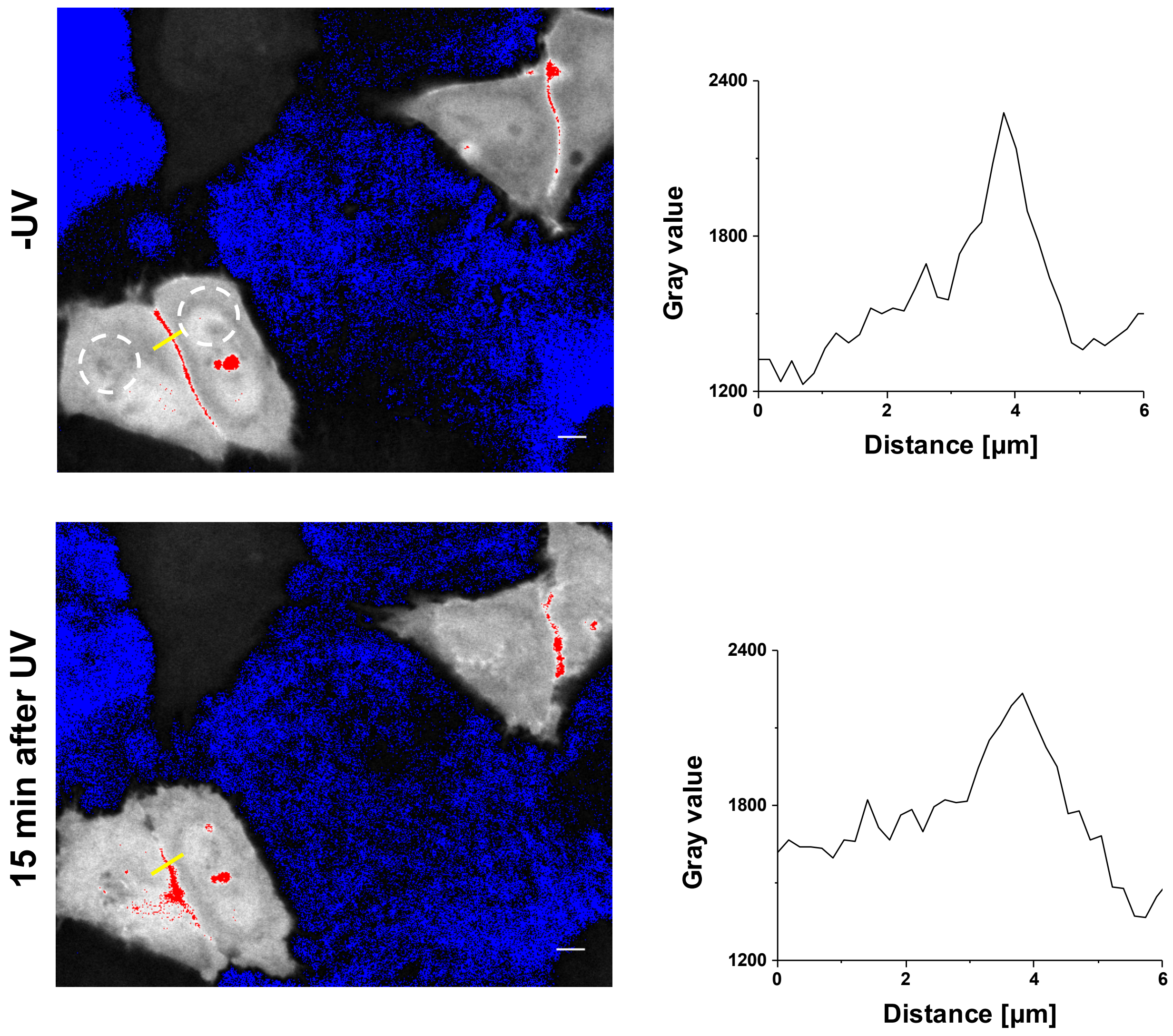

Supplementary Figure 19. Green recordings after overexpression of GFP-Akt-PH, incubation with DEACM-InsP7 (-MoTr) and UV-mediated uncaging. Treatment of cells with DEACM-InsP7 **9** in the absence of MoTr **11** does not lead to efficient loading of cells. After uncaging (dashed circles), no significant decrease in membrane fluorescence is observed. Scale bars: 5 µm.

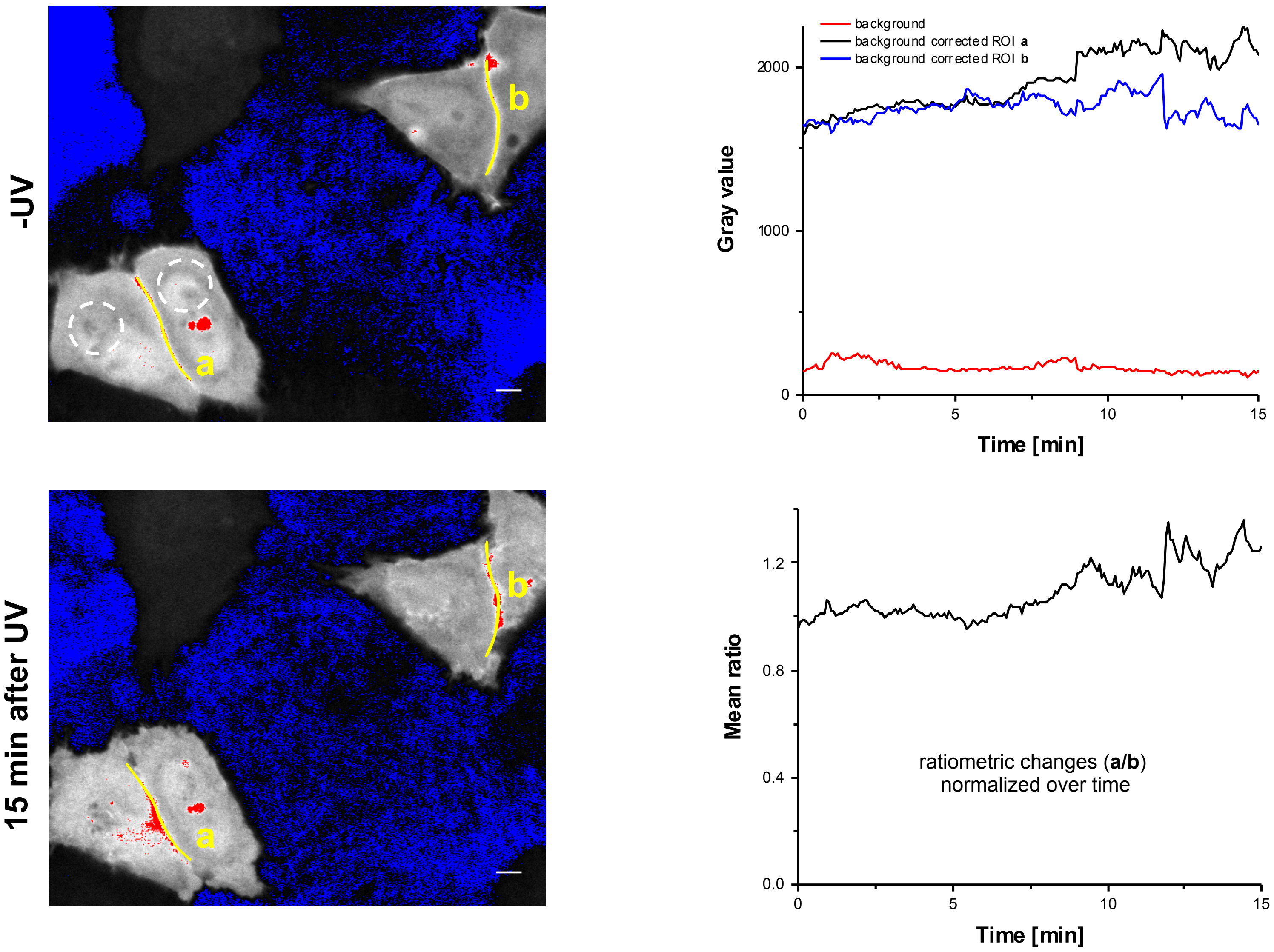

Supplementary Figure 20. Green recordings after overexpression of GFP-Akt-PH, incubation with DEACM-InsP7 (-MoTr) and UV-mediated uncaging. The membrane signal remained constant over the observation time – even after application of the UV pulse. UV-settings in presented control experiments were the same as before in presence of DEACM-InsP7. Ratiometric changes over the observation time were determined by normalizing the membrane signal **a** to the signal of the control section **b.** Intensity signals are represented background corrected. Intensities were acquired pre-saturated, with the entire dynamic range of intensity available. Images are presented in pseudo-color, normalized over time. Scale bars: 5 μm.

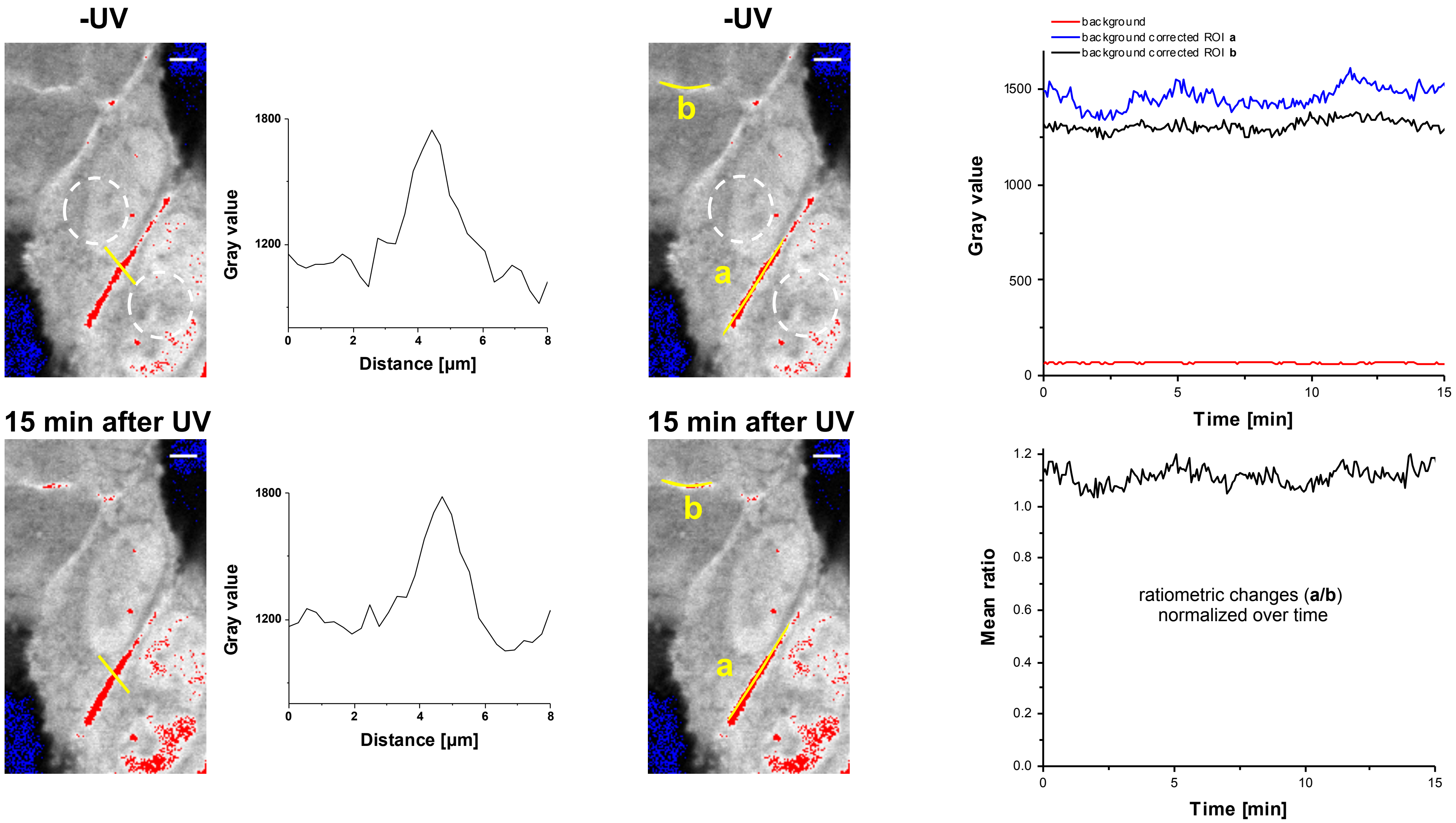

Supplementary Figure 21. Green channel recordings of GFP-Akt-PH (-DEACM-InsP7 / -MoTr). The membrane signal remained constant over the observation time – even after application of the UV pulse. UV-settings in presented control experiments were the same as before in presence of DEACM-InsP7. Ratiometric changes over the observation time were determined by normalizing the membrane signal **a** to the signal of the control section **b.** Intensity signals are represented background corrected. Intensities were acquired pre-saturated, with the entire dynamic range of intensity available. Images are presented in pseudo-color, normalized over time. Scale bars: 5 µm.

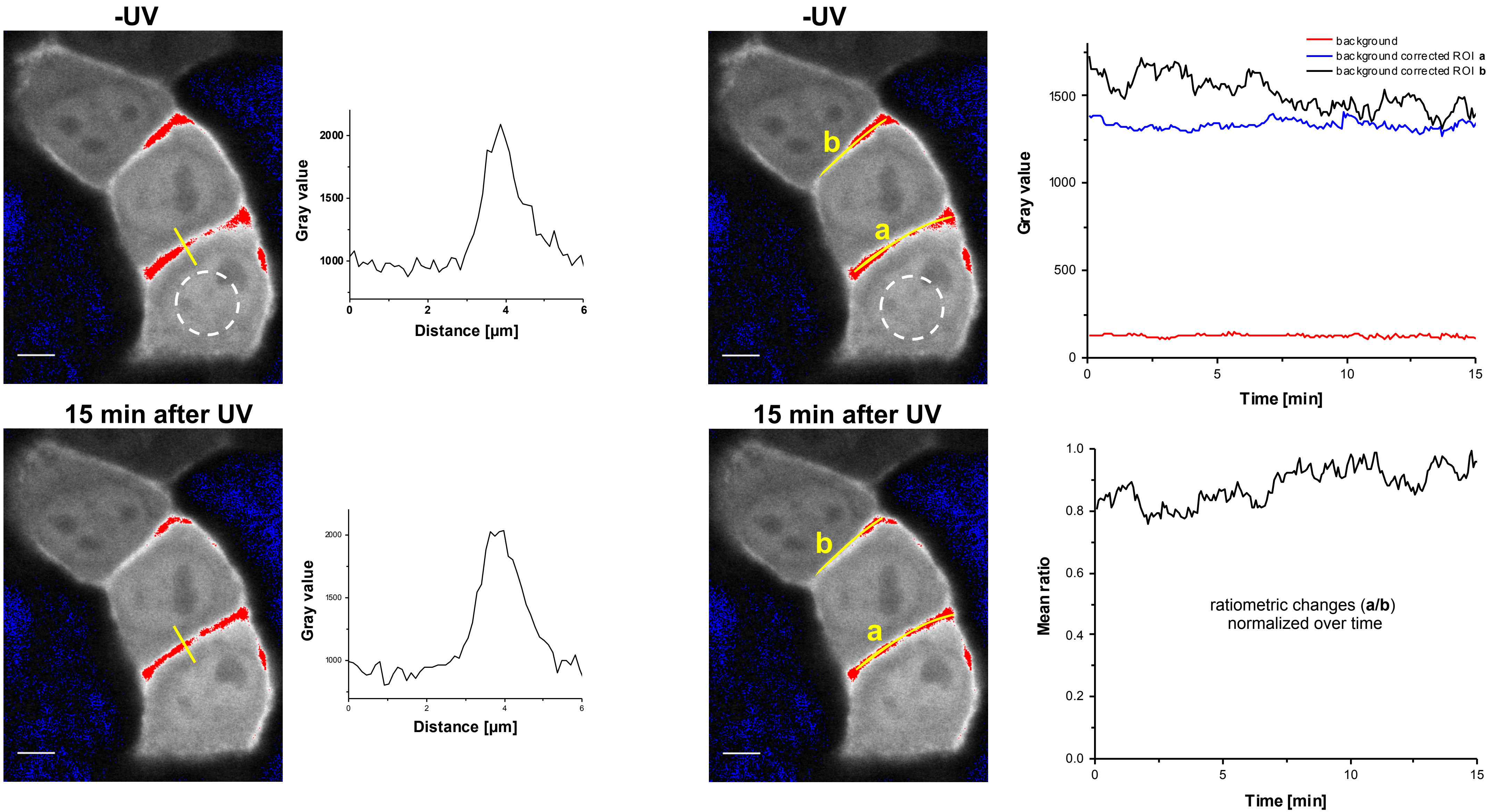

Supplementary Figure 22. Green channel recordings of GFP-Akt-PH (-DEACM-InsP7 / +MoTr). The membrane signal remained constant over the observation time – even after application of the UV pulse (cross-section). UV-settings in presented control experiments were the same as before in presence of DEACM-InsP7. Ratiometric changes over the observation time were determined by normalizing the membrane signal **a** to the signal of the control section **b.** Intensities were acquired pre-saturated, with the entire dynamic range of intensity available. Images are presented in pseudo-color, normalized over time. Scale bars: 5 µm.

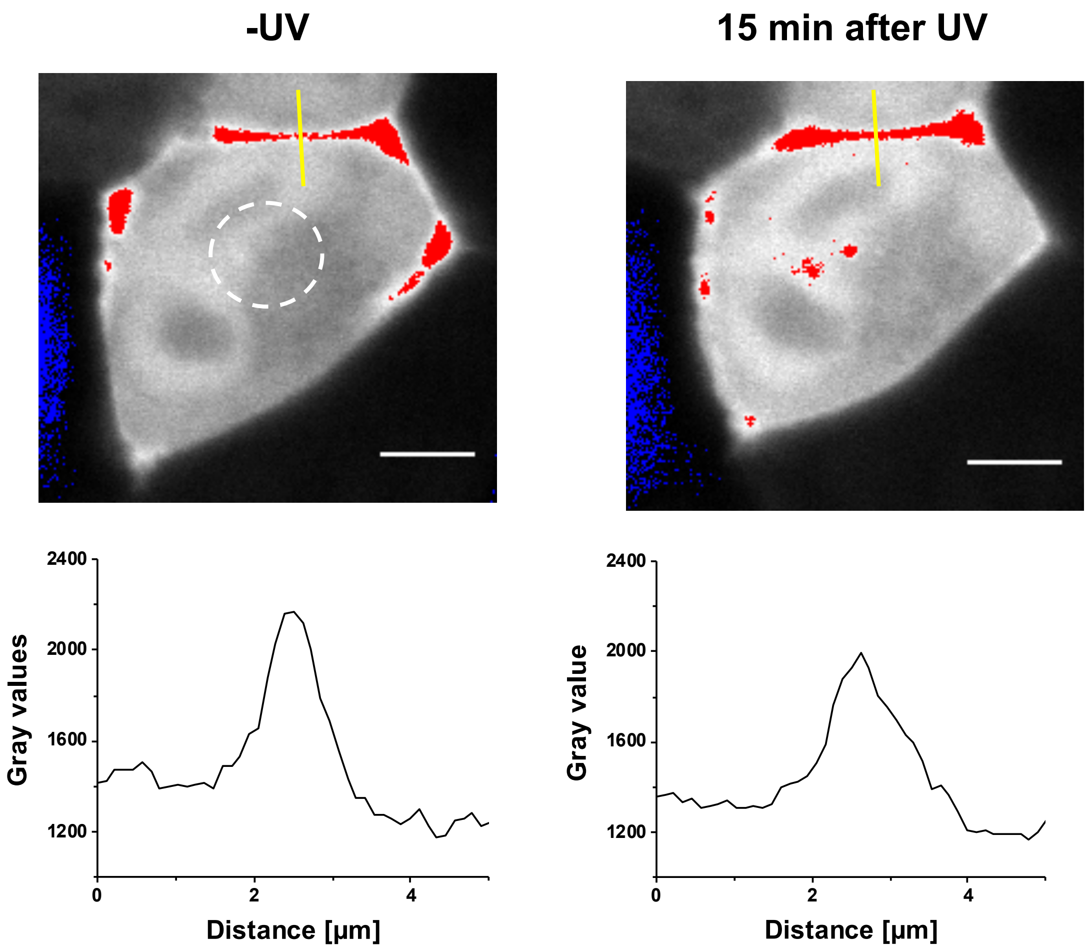

Supplementary Figure 23. Green channel recordings of GFP-Akt-PH (-DEACM-InsP7 / +MoTr). The membrane signal remained constant over the observation time – even after application of the UV pulse. UV-settings in presented control experiments were the same as before in presence of DEACM-InsP7 **9**. Changes in the overall intensity distribution (especially in the right corner of the cell) are attributed to movements of the cell under investigation during observation time. Intensities were acquired pre-saturated, with the entire dynamic range of intensity available. Images are presented in pseudo-color, normalized over time. Scale bars: 5 µm.

Compound 3b, 1H

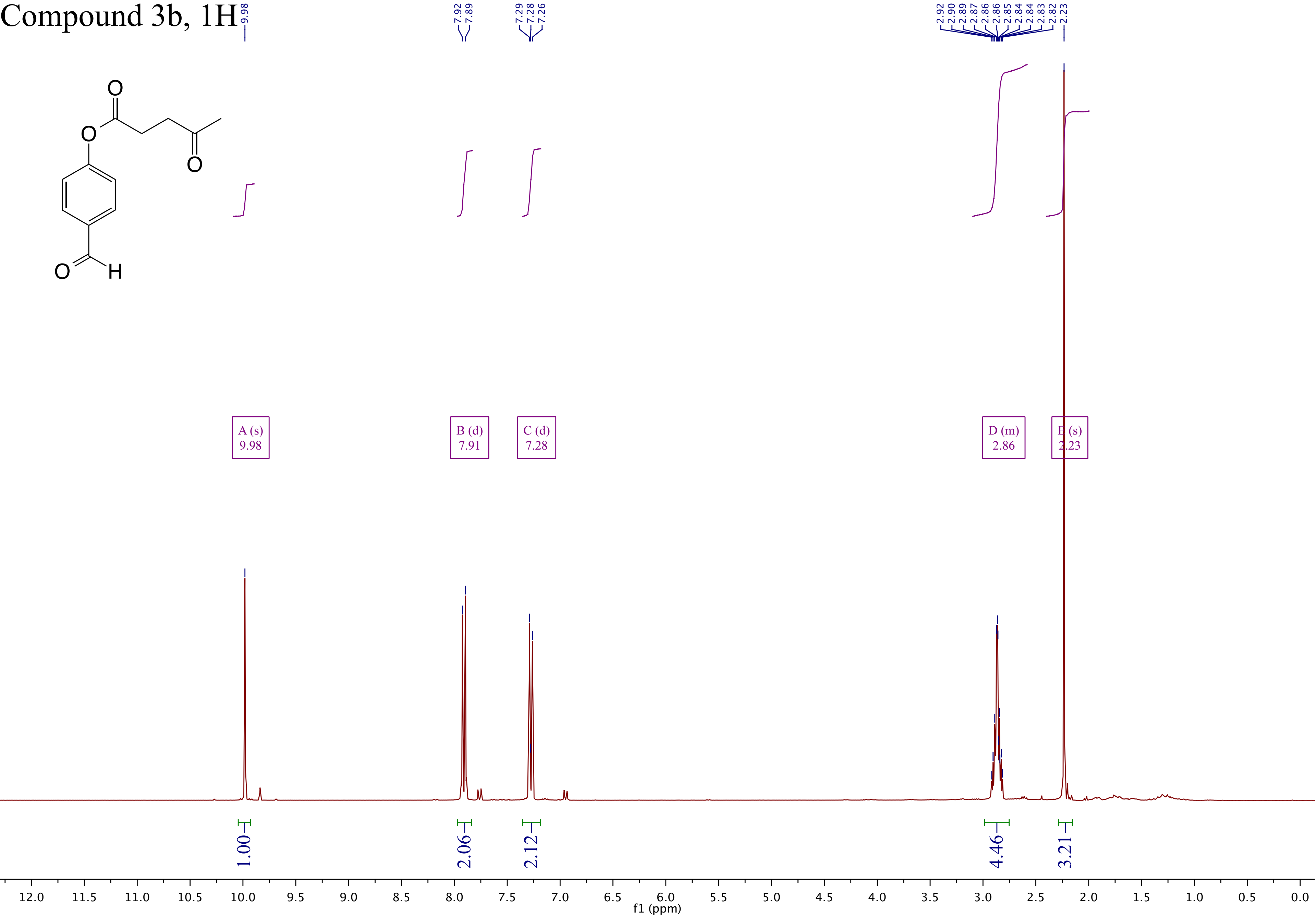

Supplementary Figure 24. 1H NMR of compound 3b.

Compound 3b, 13C

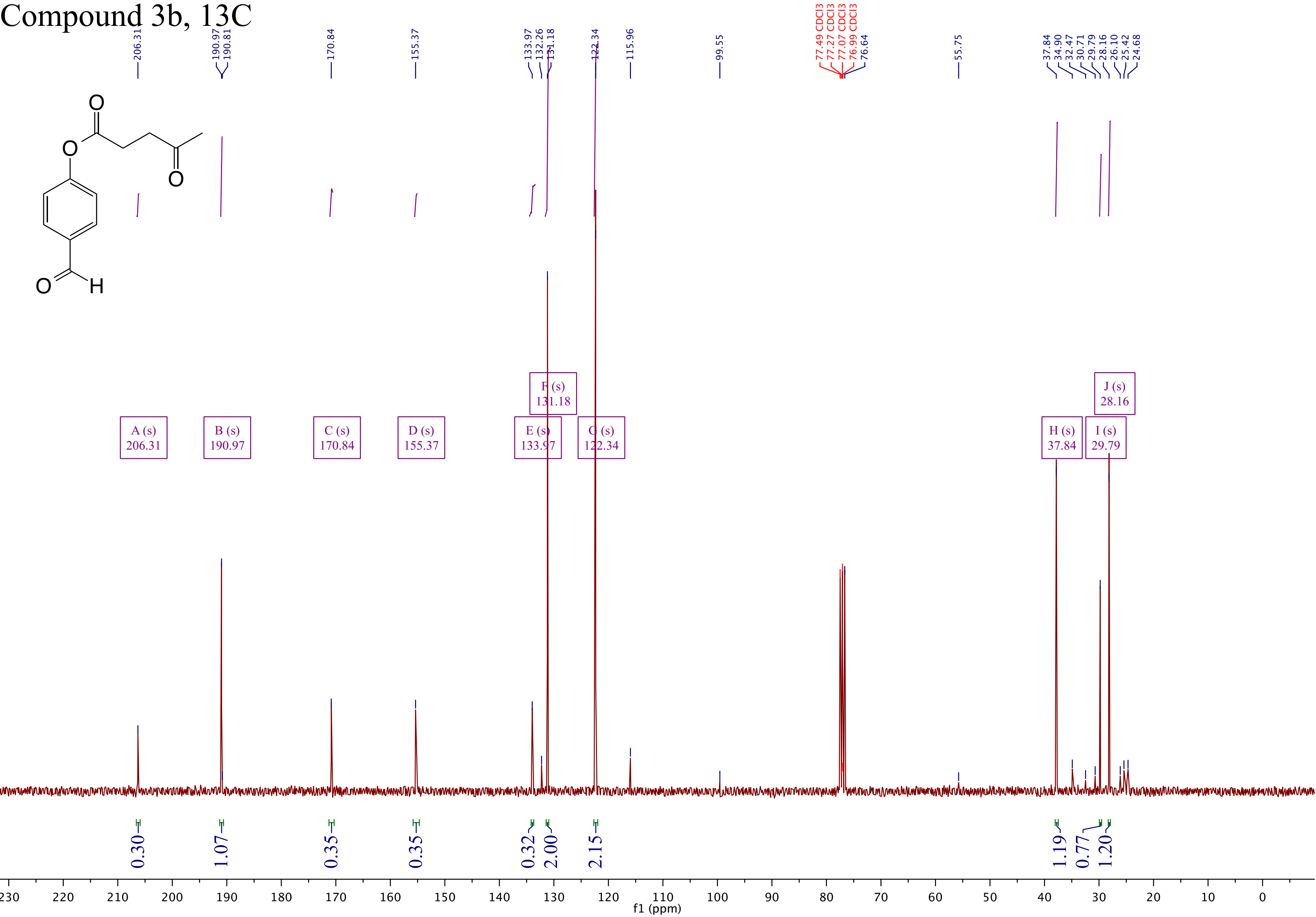

Supplementary Figure 25. 13C NMR of compound 3b

HR-ESI-MS (Bruker maXis)

Analysis Info

Analysis Name	D:\Data\Service\6814sihres.d	Acquisition Date	8/29/2014 12:38:44 PM	
Method	tune_low_modified_09_01_14_pos.m	Operator	ust	
Sample Name	IP-3-82P1	Instrument	maXis	255552.00033
Comment	Solvent: MeOH + NaOH			
	Client: Unzue			

Acquisition Parameter

Source Type	ESI	Ion Polarity	Positive	Set Nebulizer	0.4 Bar
Scan Begin	50 m/z	Set Capillary	1600 V	Set Dry Heater	180 °C
Scan End	3000 m/z	Set End Plate Offset	-500 V	Set Dry Gas	4.0 l/min

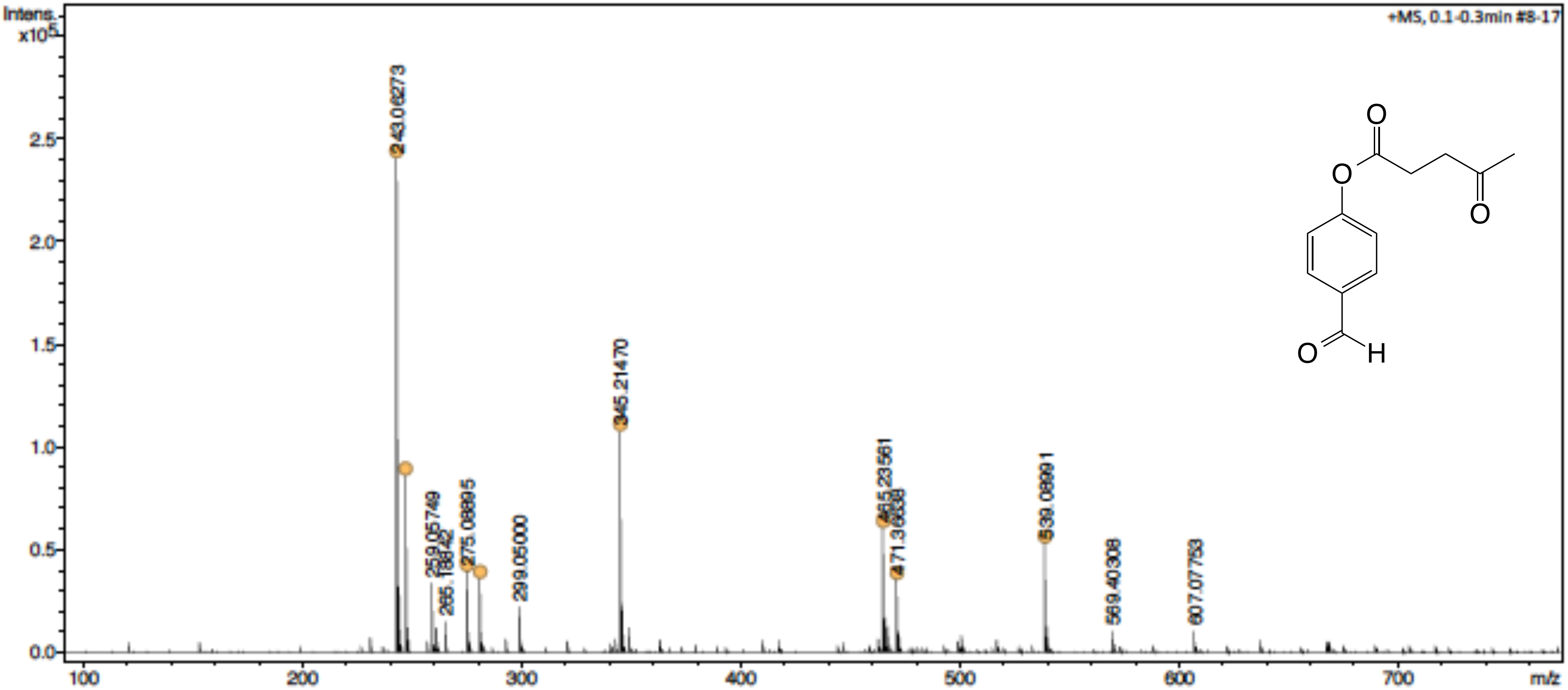

Supplementary Figure 26. ESI MS analysis of compound 3b

Compound 3c, 1H

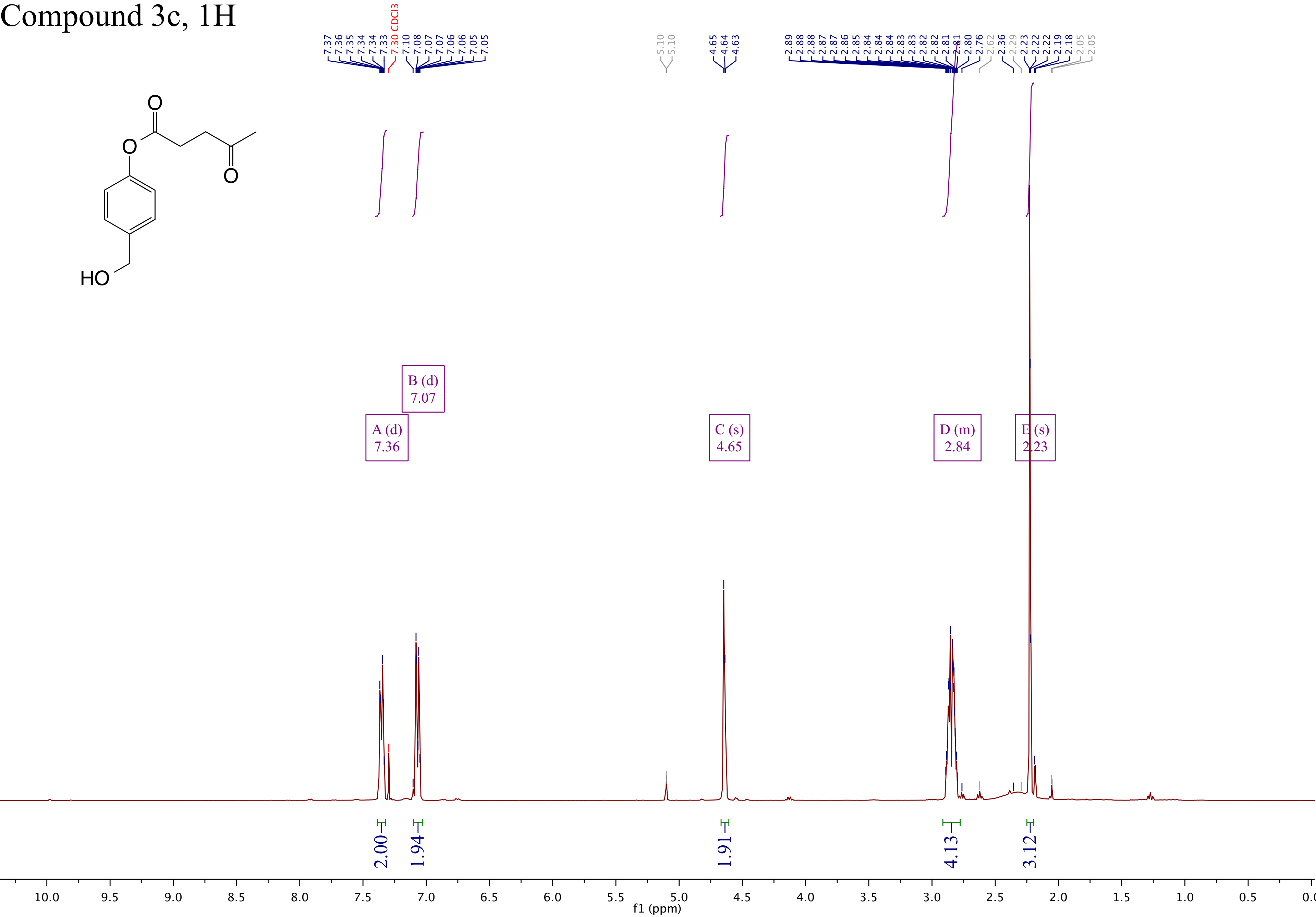

Supplementary Figure 27. 1H NMR of compound 3c.

Compound 3c, 13C

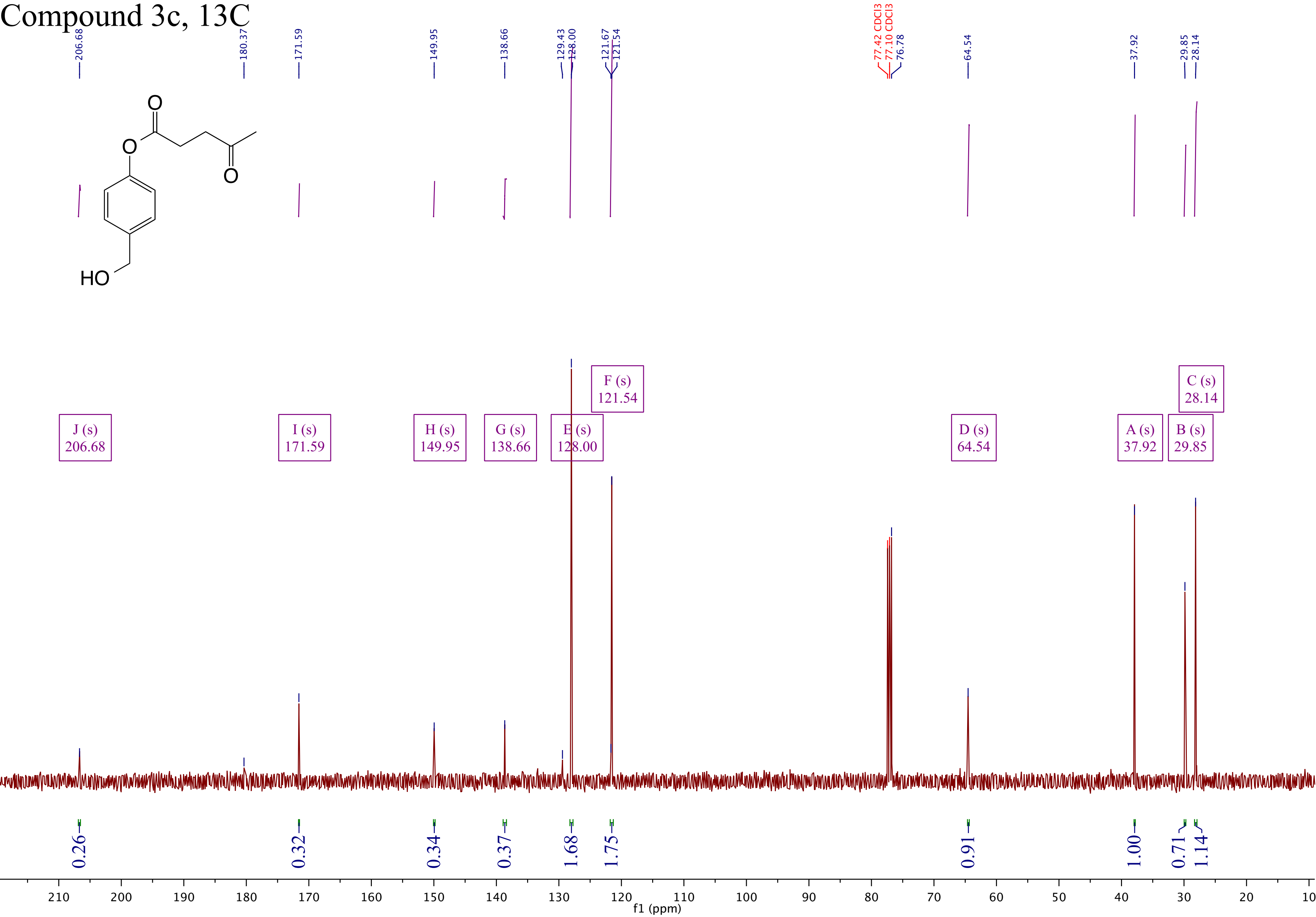

Supplementary Figure 28. 13C NMR of compound 3c

HR-ESI-MS (Bruker maXis)

Analysis Info

Analysis Name	D:\Data\Service\6815sihres.d	Acquisition Date	8/29/2014 1:10:06 PM
Method	tune_low_modified_09_01_14_pos.m	Operator	ust
Sample Name	IP-3-91P1	Instrument	maXis 255552.00033
Comment	Solvent: MeOH + NaOH		
	Client: Pavlovic		

Acquisition Parameter

Source Type	ESI	Ion Polarity	Positive	Set Nebulizer	0.4 Bar
Scan Begin	50 m/z	Set Capillary	1600 V	Set Dry Heater	180 °C
Scan End	3000 m/z	Set End Plate Offset	-500 V	Set Dry Gas	4.0 l/min

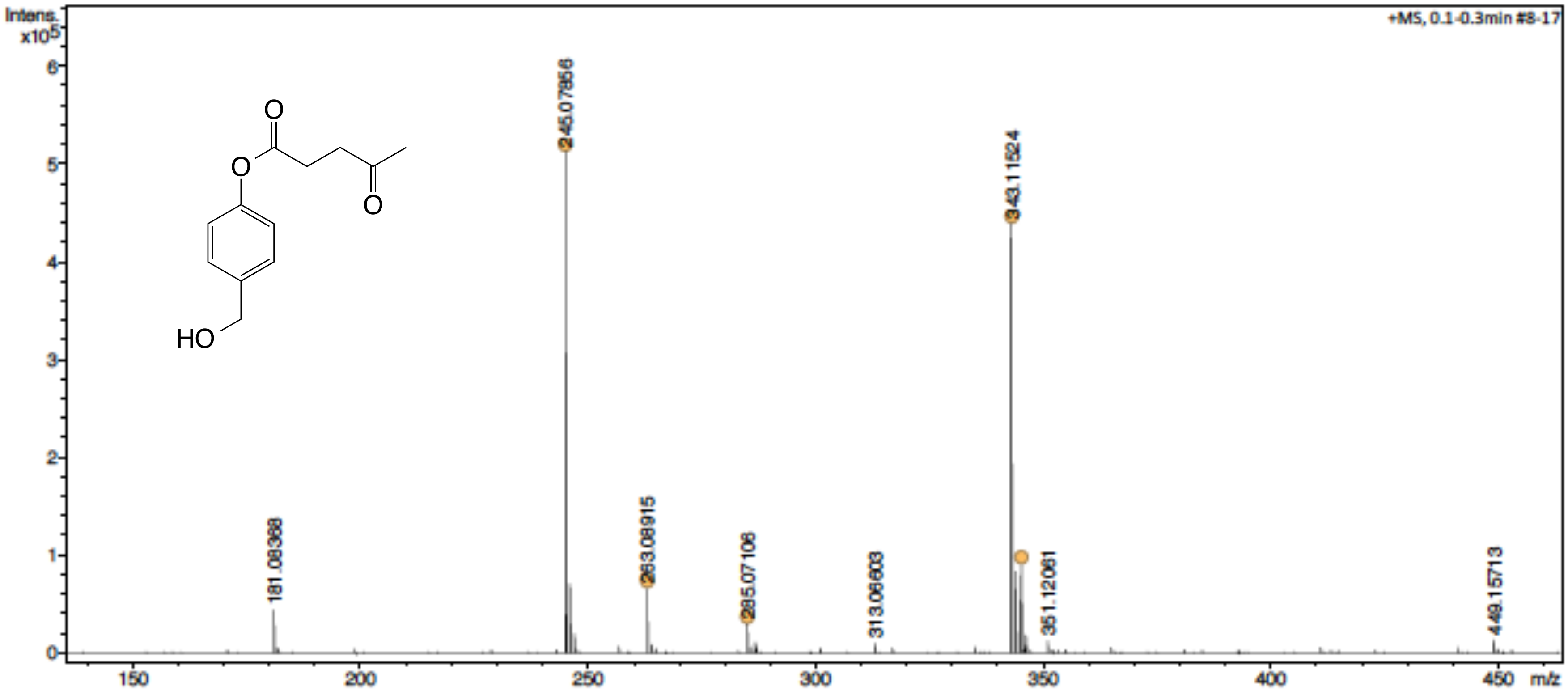

Supplementary Figure 29. ESI MS analysis of compound 3c.

Compound 3, 1H

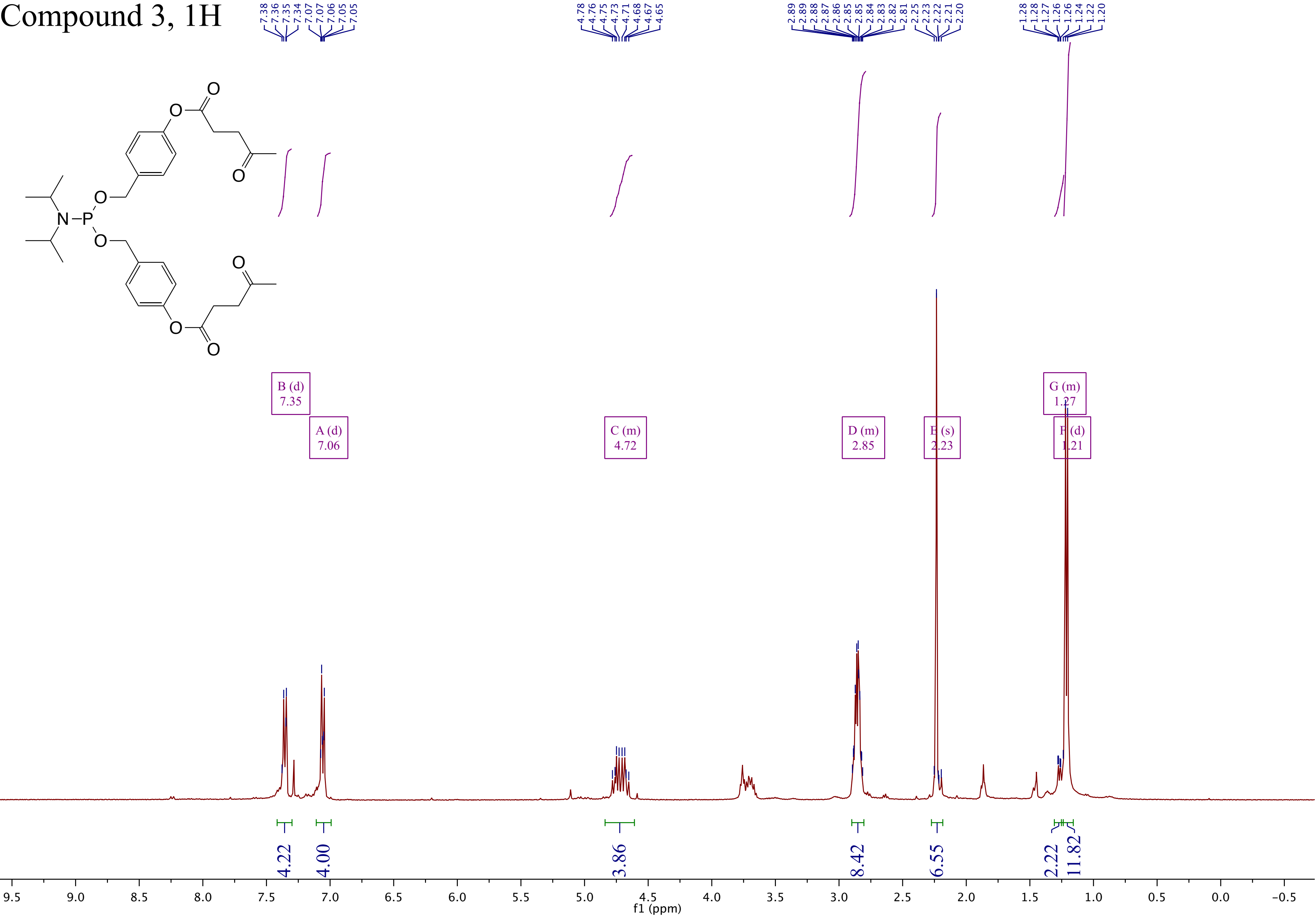

Supplementary Figure 30. 1H NMR of compound 3.

Compound 3, 13C

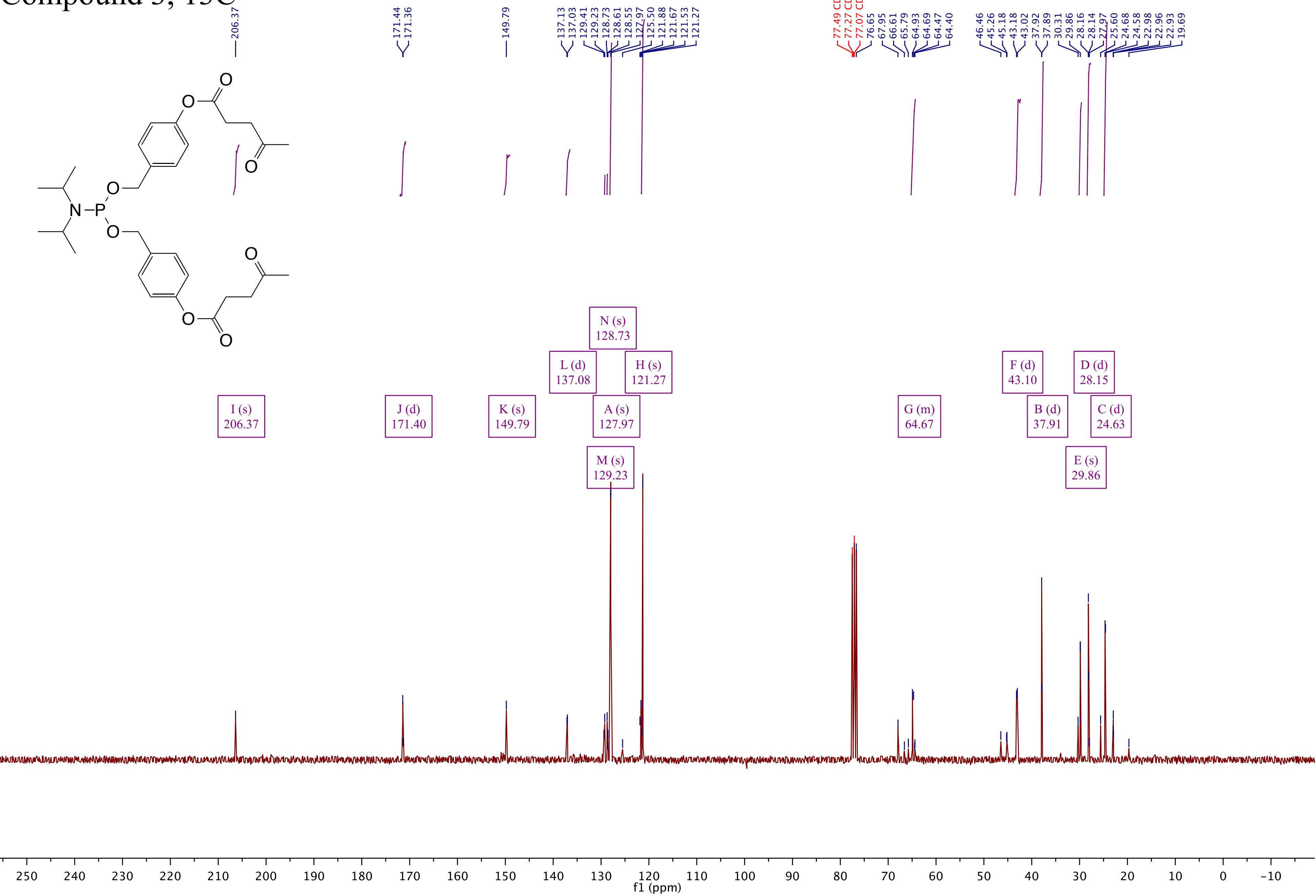

Supplementary Figure 31. 13C NMR of compound 3.

Compoun 3, 31P 1H decoupled

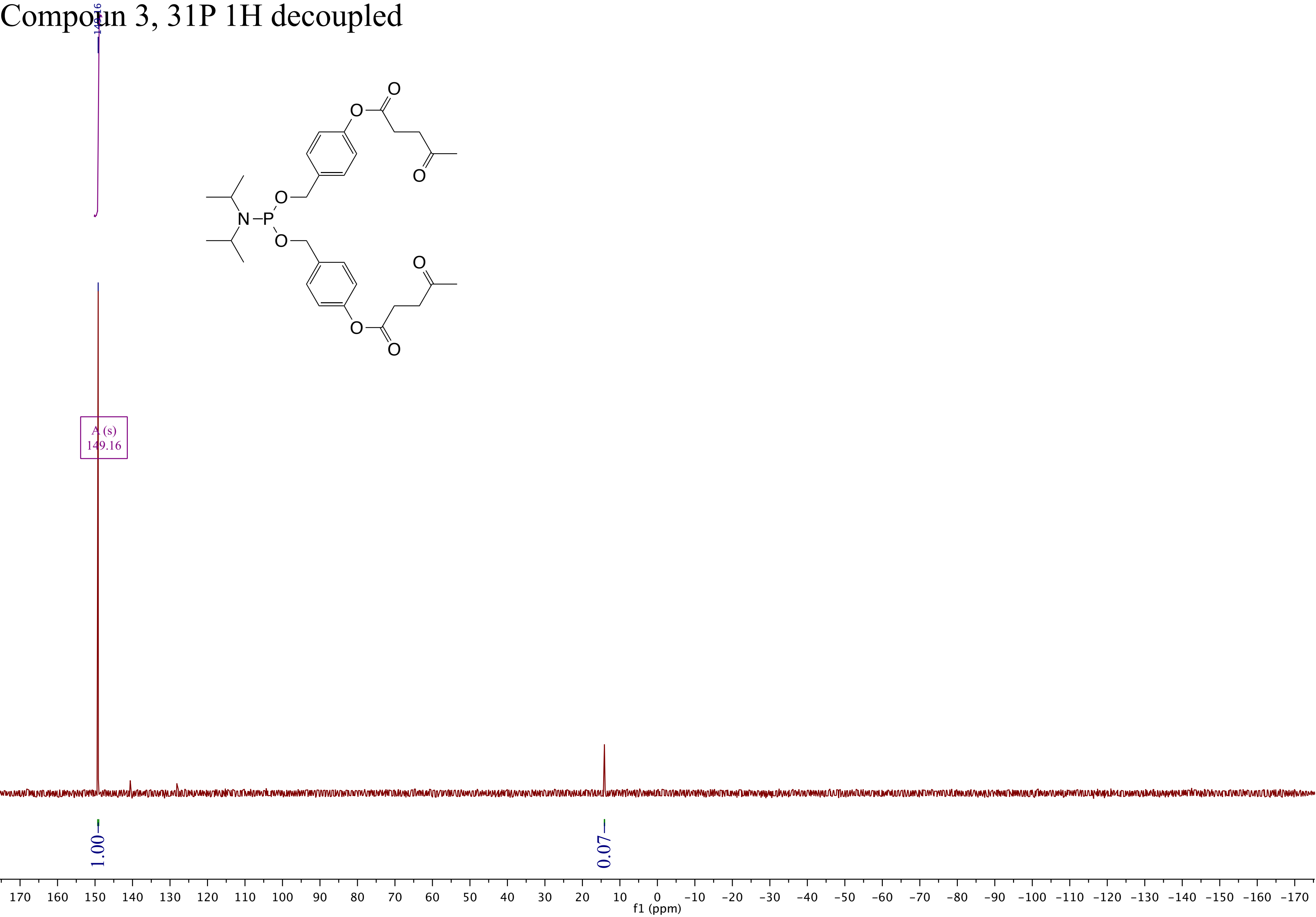

Supplementary Figure 32. 31P NMR of compound 3.

Compound 3, 31P 1H coupled

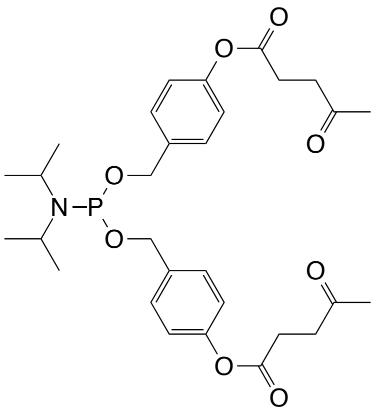

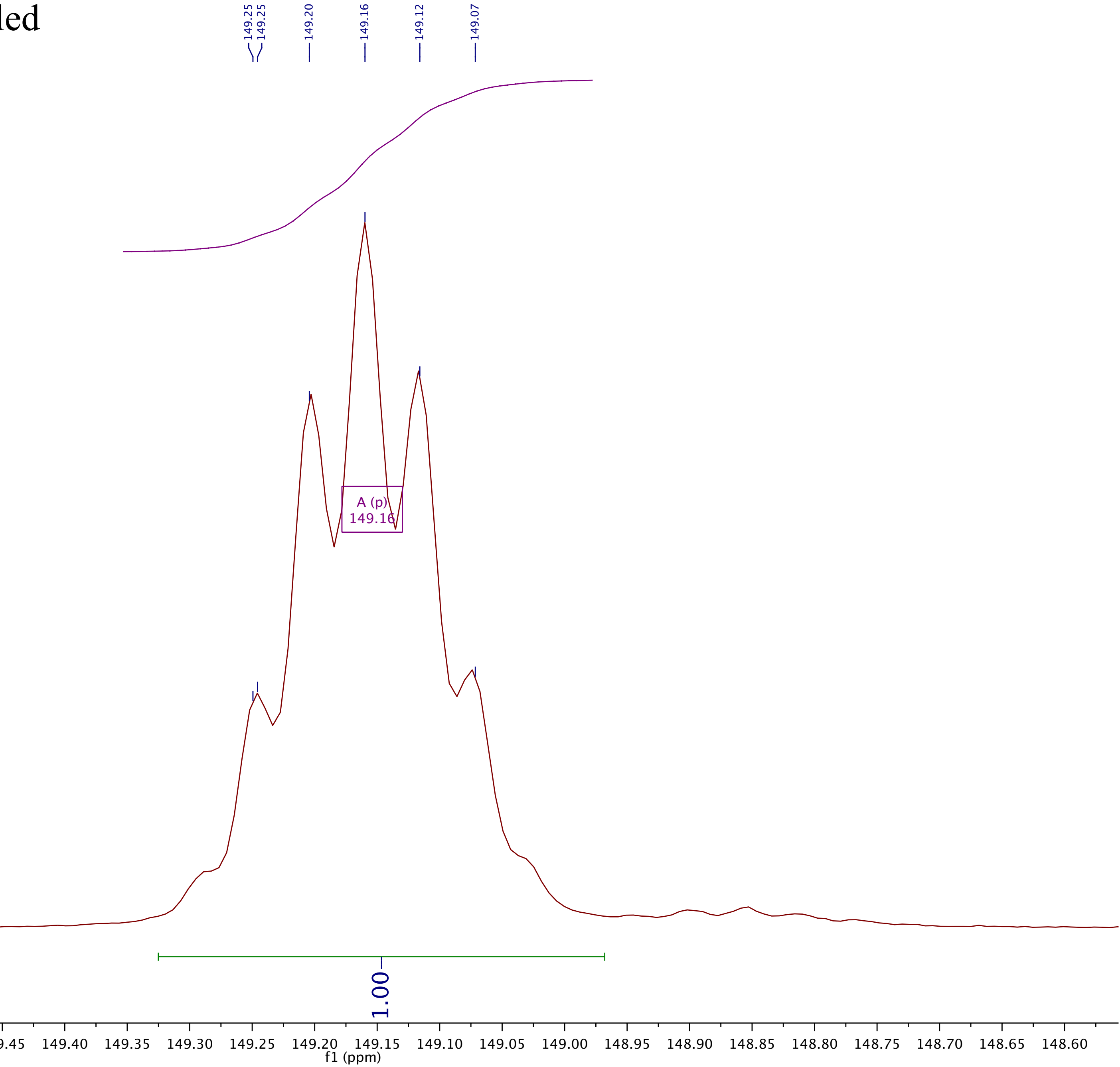

Supplementary Figure 33. 31P NMR 1H coupled of compound 3.

HR-ESI-MS (Bruker maXis)

Analysis Info

Analysis Name	D:\Data\Service\6816sihres.d	Acquisition Date	8/29/2014 2:50:58 PM
Method	tune_low_modified_09_01_14_pos.m	Operator	ust
Sample Name	IP-3-91P1	Instrument	maXis 255552.00033
Comment	Solvent: MeOH + NaI		
	Client: Pavlovic		

Acquisition Parameter

Source Type	ESI	Ion Polarity	Positive	Set Nebulizer	0.4 Bar
Scan Begin	50 m/z	Set Capillary	4000 V	Set Dry Heater	180 °C
Scan End	3000 m/z	Set End Plate Offset	-500 V	Set Dry Gas	4.0 l/min

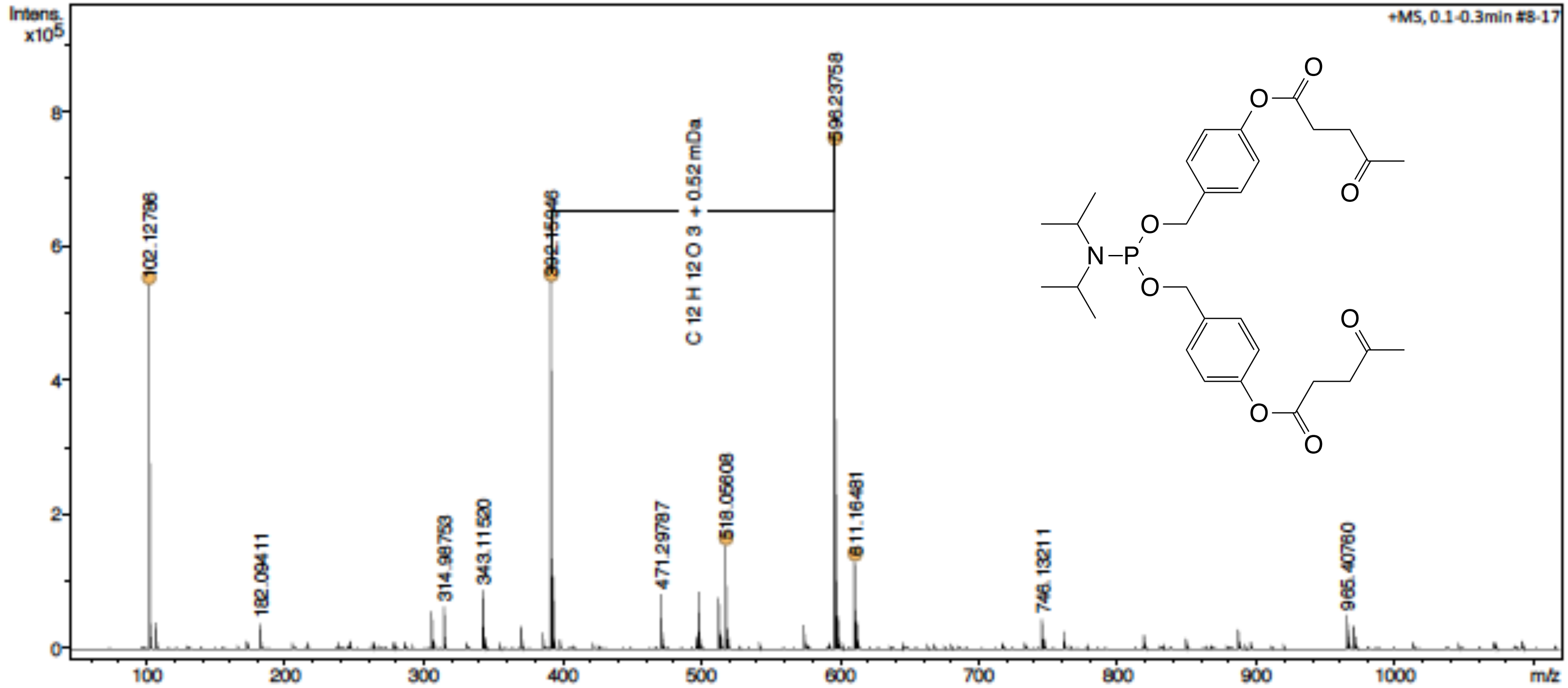

Supplementary Figure 34. ESI MS of compound 3.

Compound 4, 1H

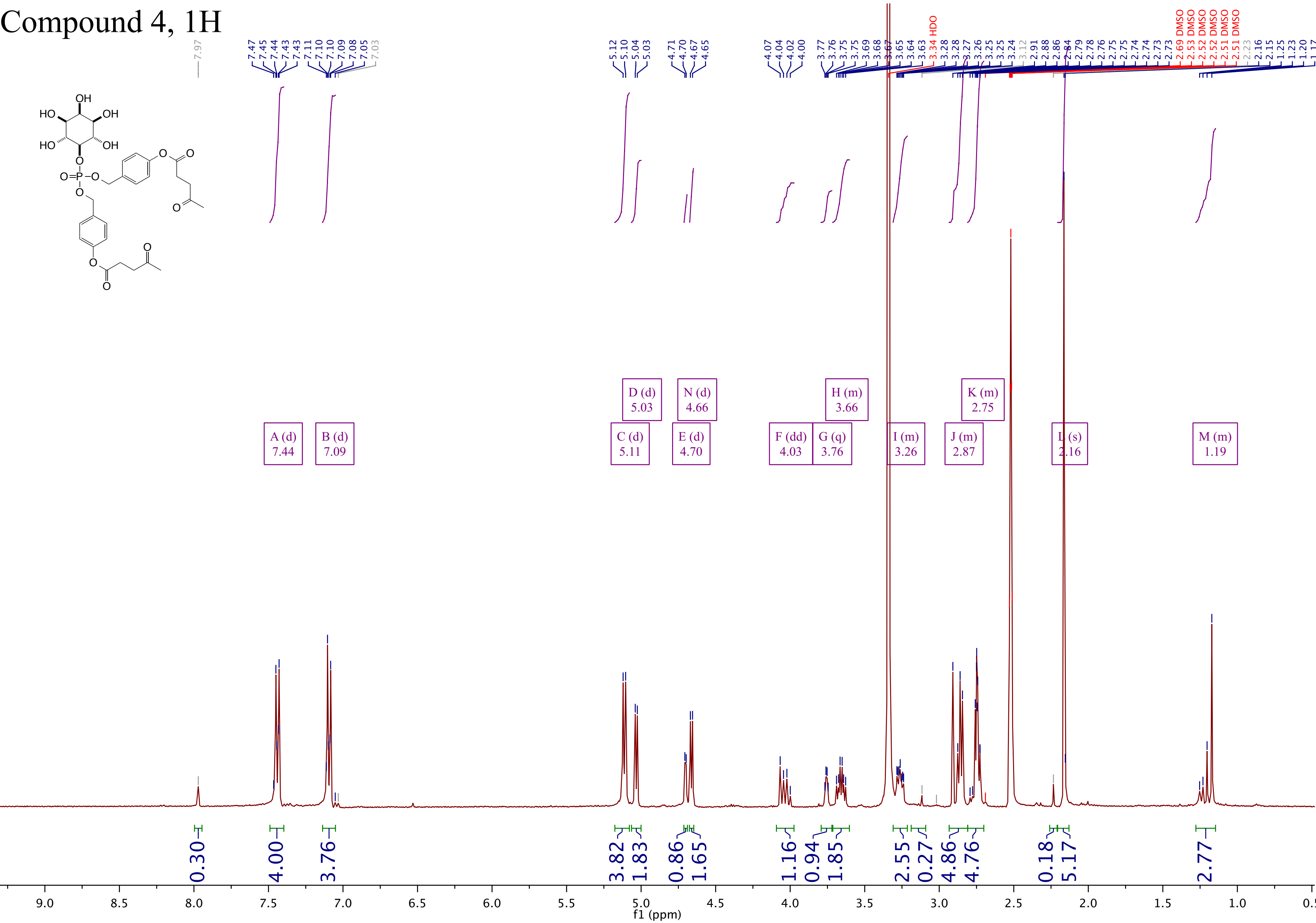

Supplementary Figure 35. 1H NMR of compound 4.

Compound 4, 13C

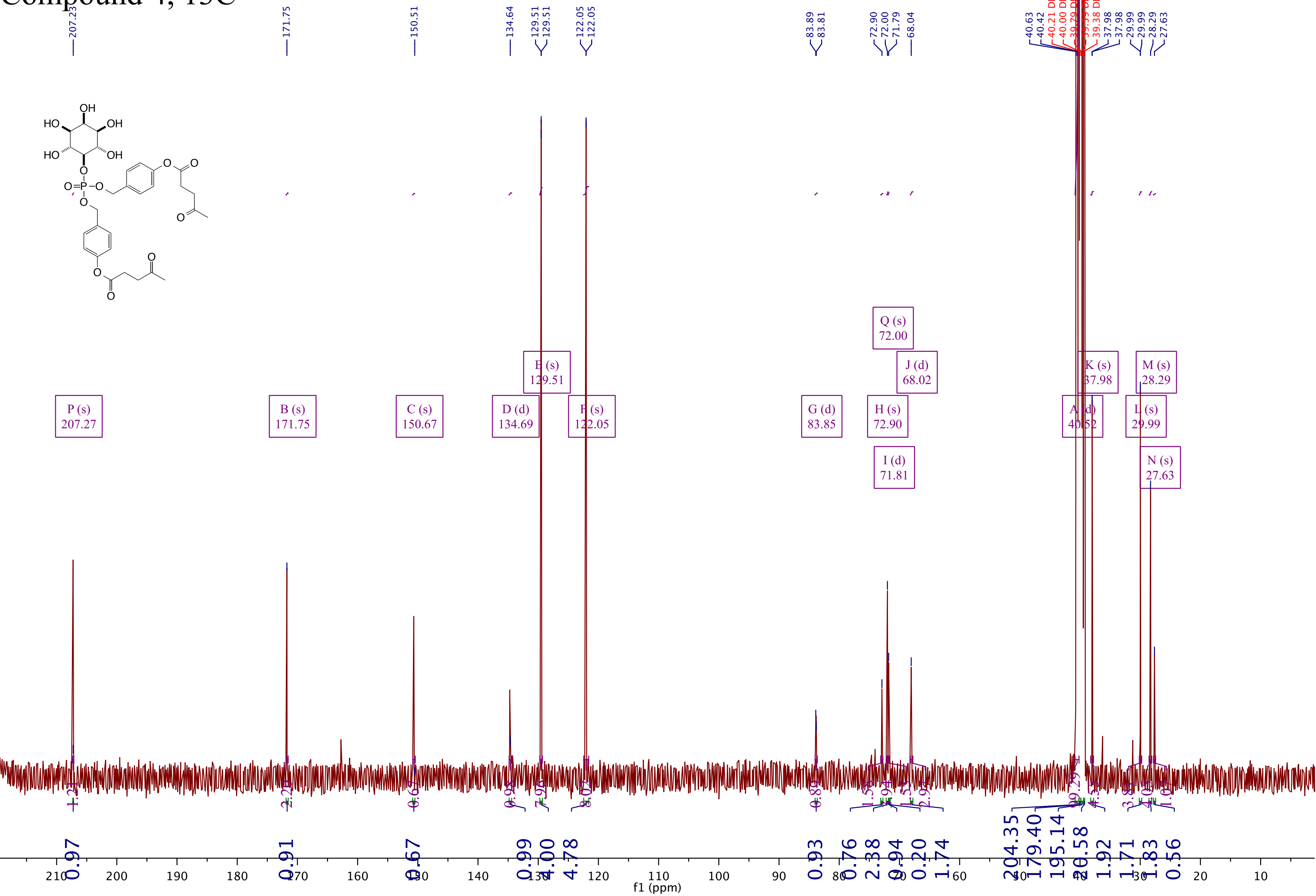

Supplementary Figure 36. 13C NMR of compound 4.

Compound 4, 31P 1H decoupled

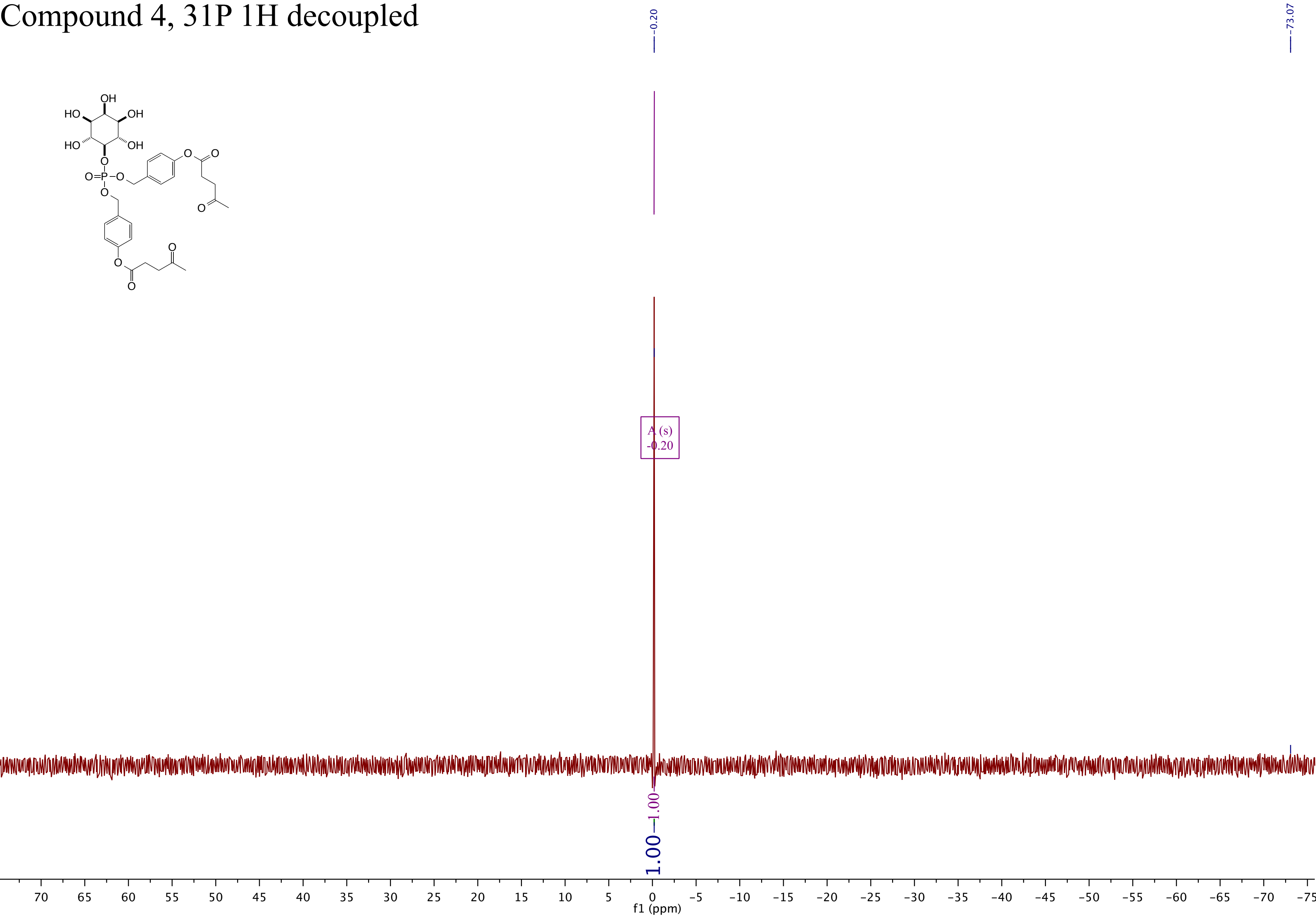

Supplementary Figure 37. 31P NMR of compound 4.

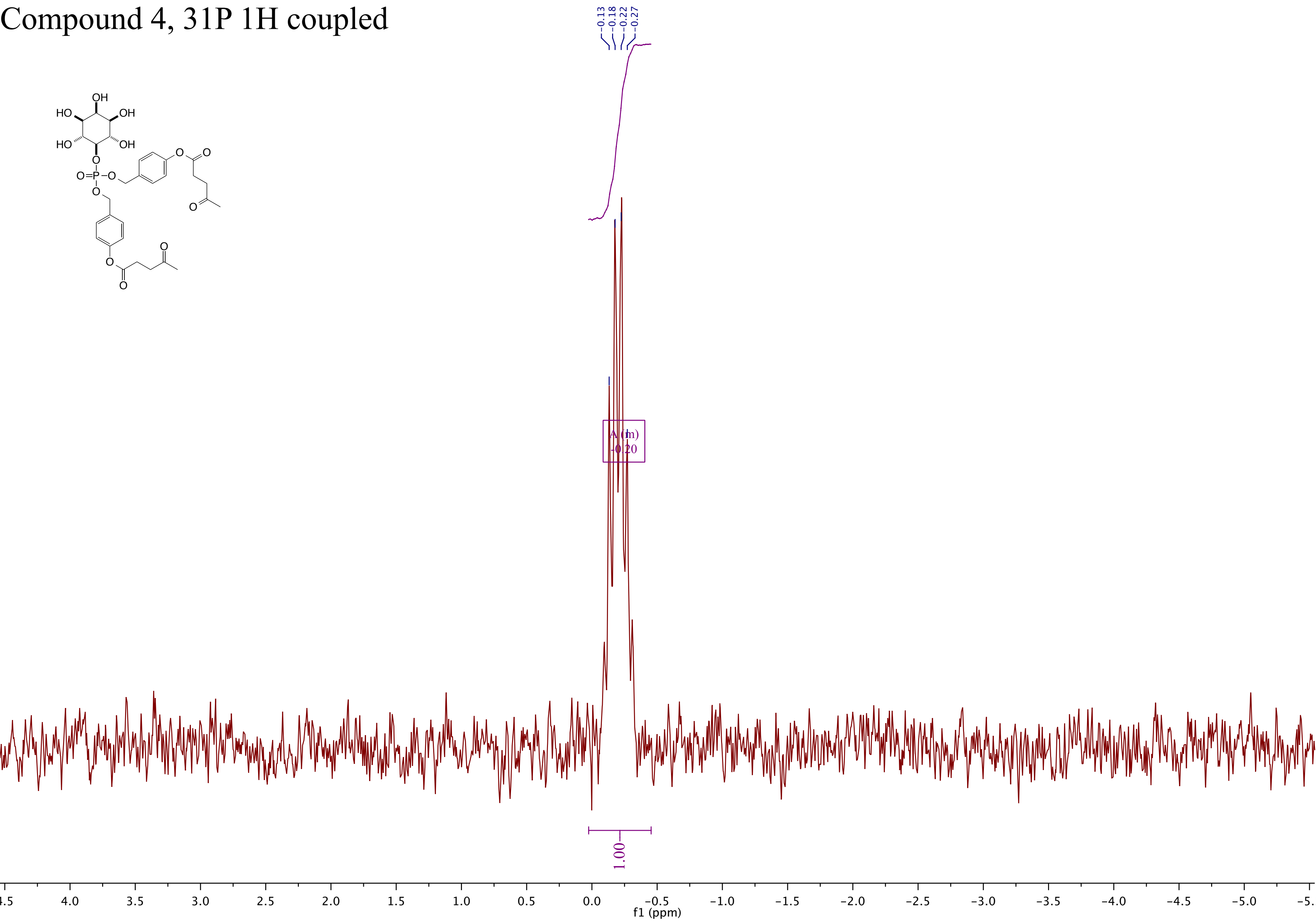

Supplementary Figure 38. 31P NMR 1H coupled of compound 4.

HR-ESI-MS (Bruker maXis)

Analysis Info

**Analysis Name**	D:\Data\Service\5907sihres.d	**Acquisition Date**	3/18/2014 2:30:53 PM
**Method**	tune_low_modified_09_01_14_pos.m	**Operator**	ust
**Sample Name**	IP-4-52P1	**Instrument**	maXis 255552.00033
**Comment**	Solvent: MeOH + NaOH<br>Client: Pavlovic		

Acquisition Parameter

Source Type	ESI	Ion Polarity	Positive	Set Nebulizer	0.5 Bar
Scan Begin	50 m/z	Set Capillary	2000 V	Set Dry Heater	180 °C
Scan End	3000 m/z	Set End Plate Offset	-500 V	Set Dry Gas	4.0 l/min

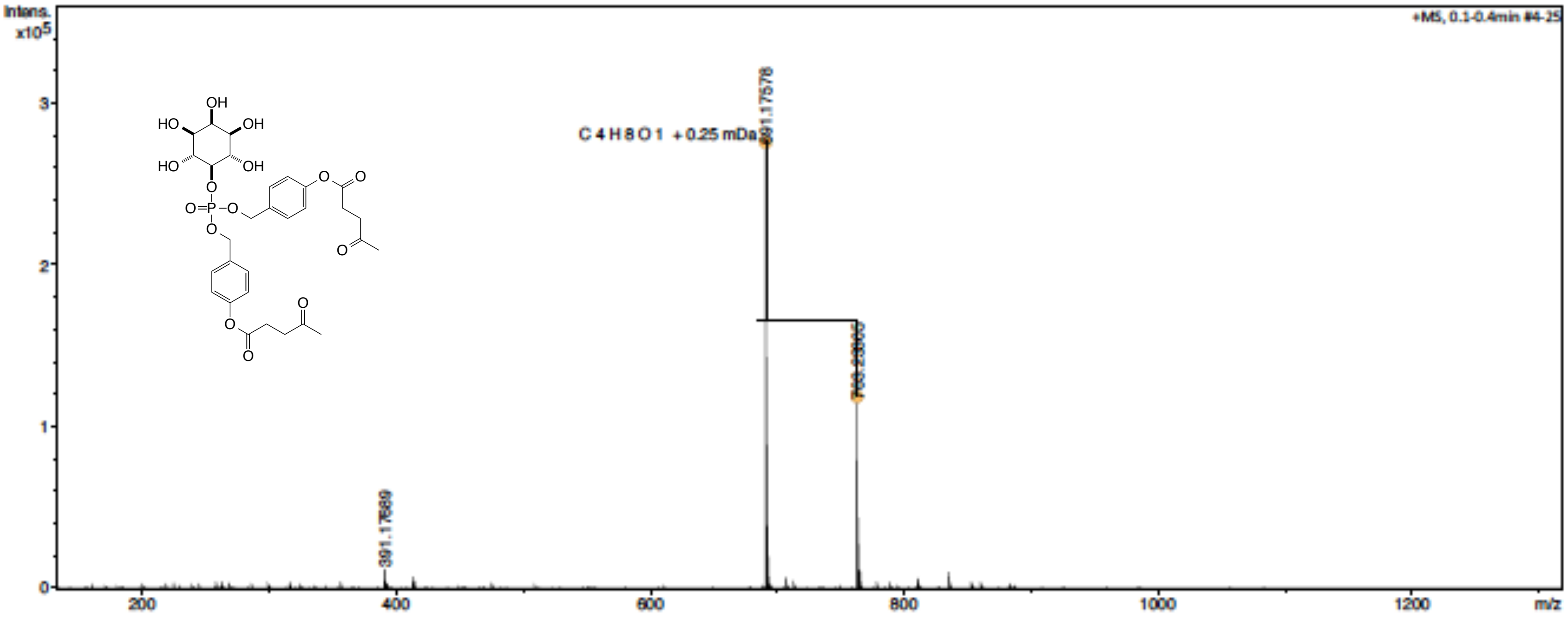

Supplementary Figure 39. ESI MS of compound 4.

Compound 6, 1H

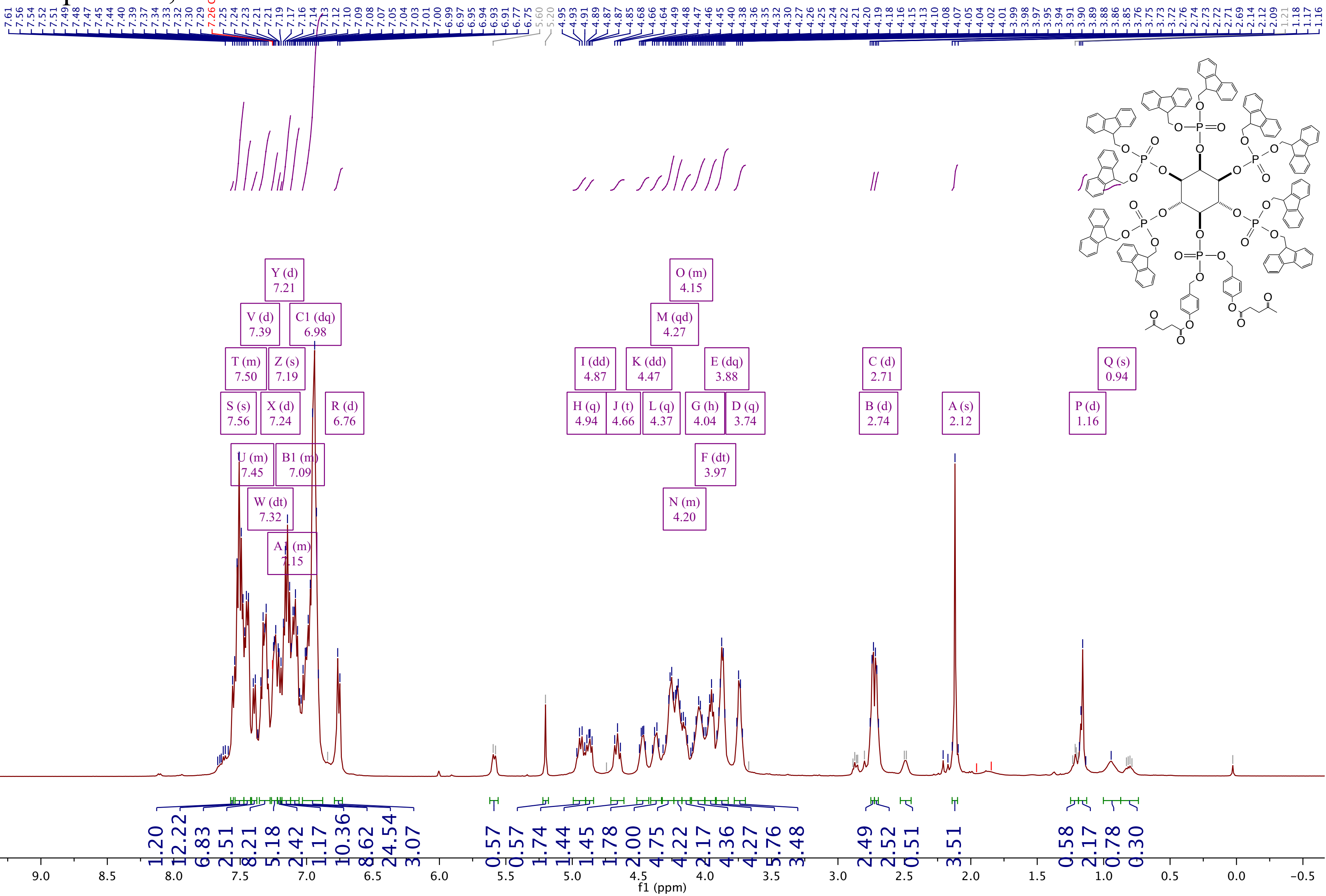

Supplementary Figure 40. 1H NMR of compound 6.

Compound 6, 13C

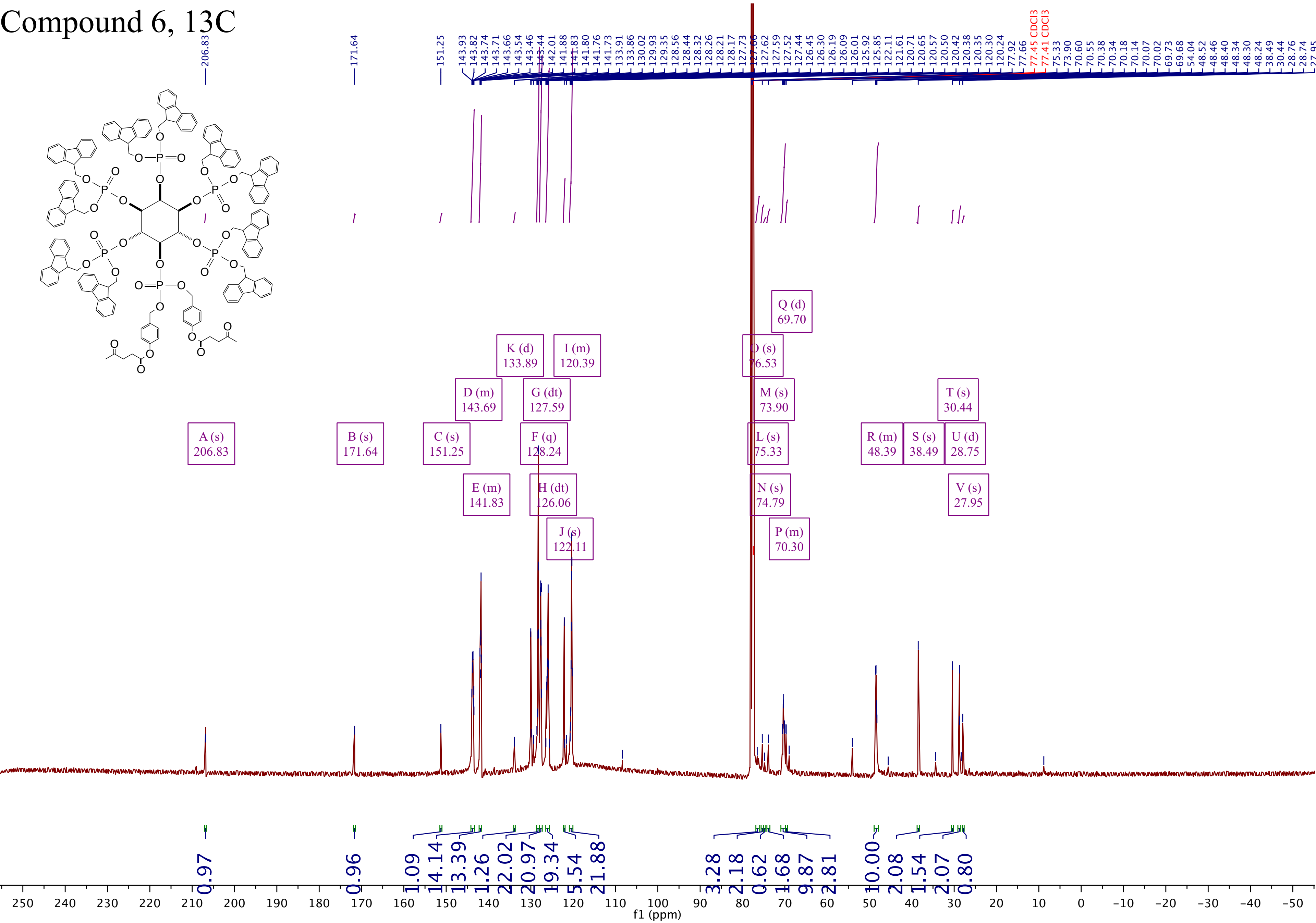

Supplementary Figure 41. 13C NMR of compound 6.

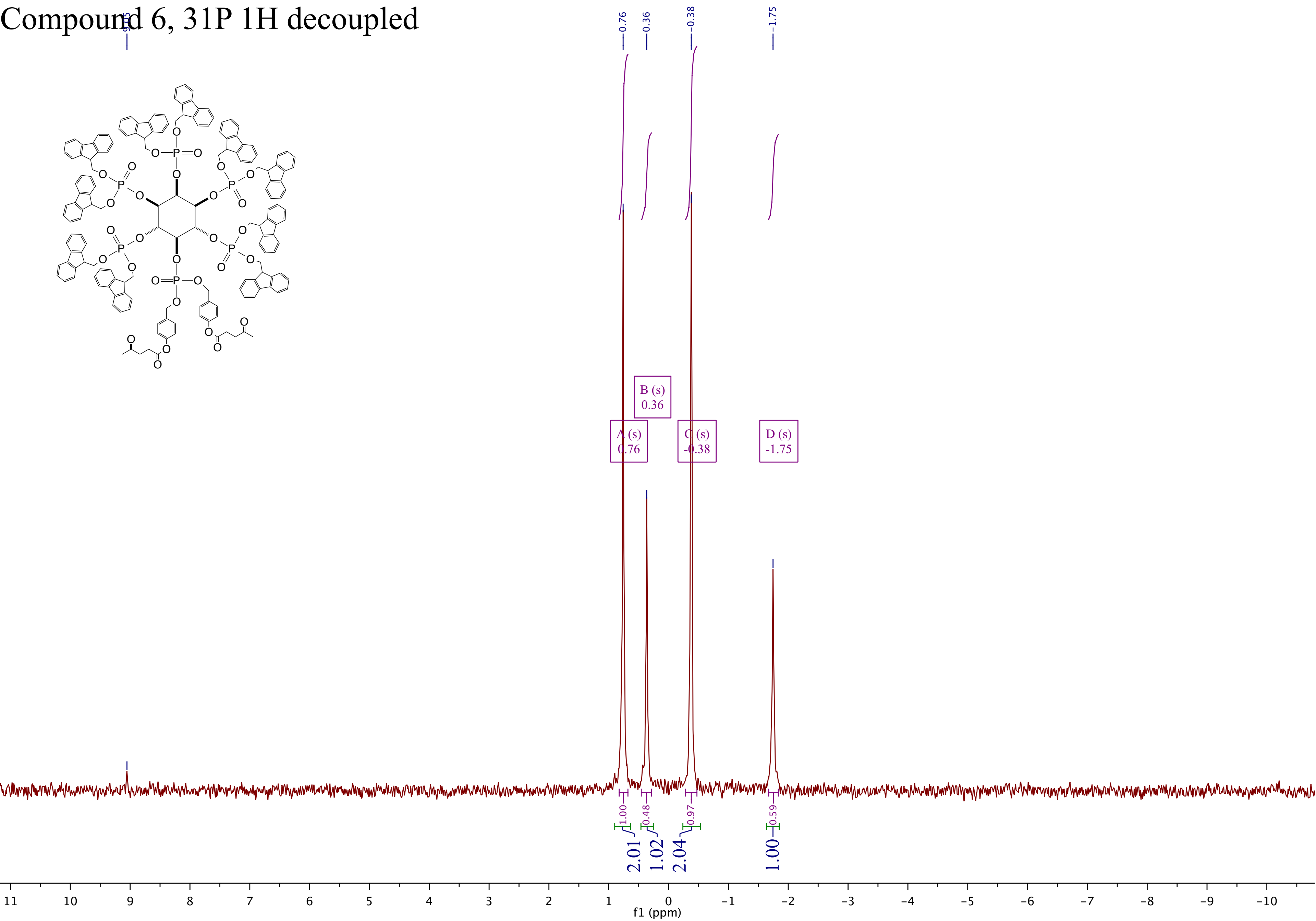

Supplementary Figure 42. 31P NMR of compound 6.

Compound 6, 31P 1H coupled

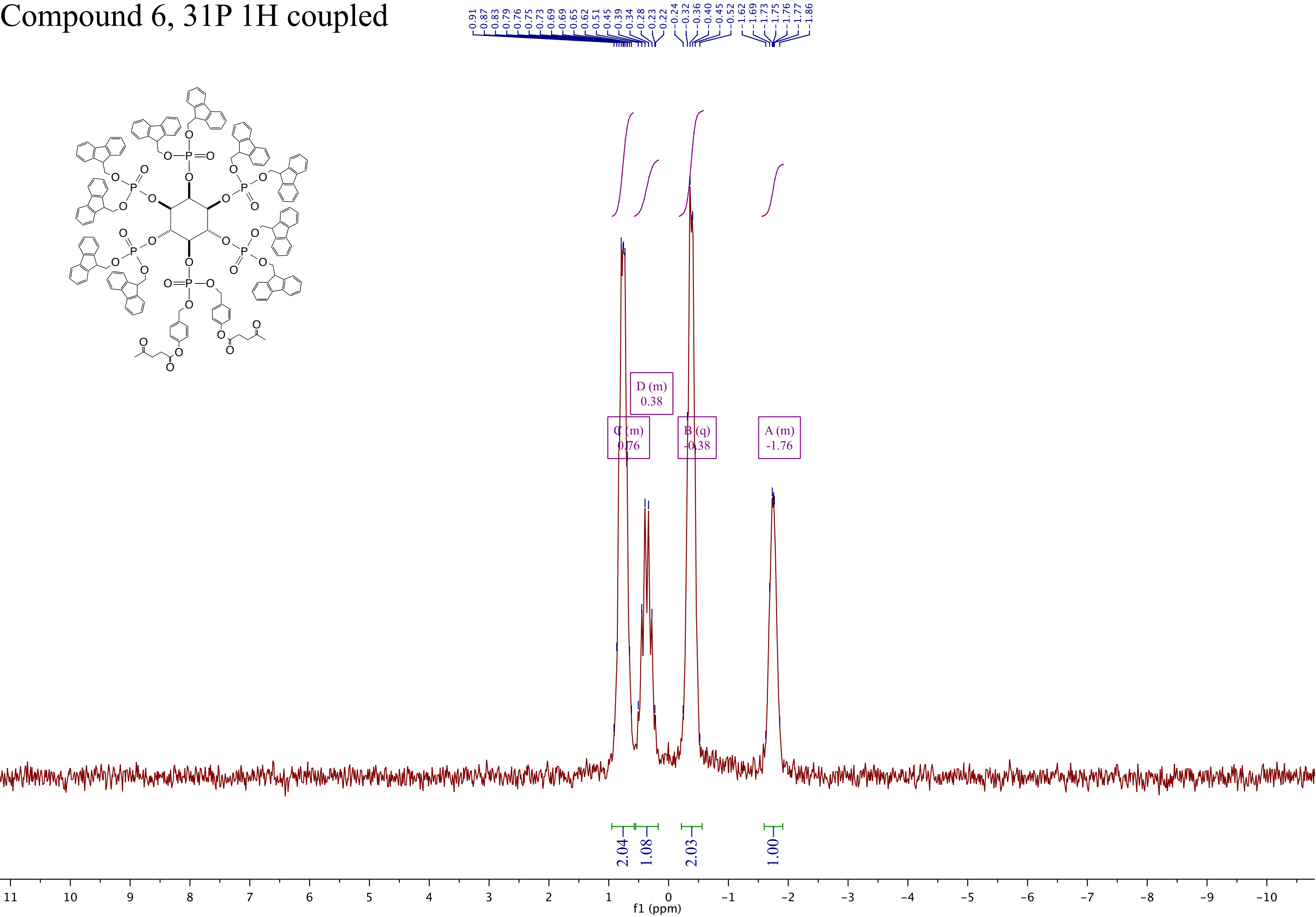

Supplementary Figure 43. 31P NMR 1H coupled of compound 6.

HR-ESI-MS (Bruker maXis)

Analysis Info

Analysis Name	D:\Data\Service\5593sihres.d	Acquisition Date	19.11.2013 15:53:54
Method	ESI_lowMR_TuneMix.m	Operator	Laurent
Sample Name	IP-4-07P1	Instrument / Ser#	maXis 33
Comment	Solvent: MeCN + NaI<br>Client: Pavlovic		

Acquisition Parameter

Source Type	ESI	Ion Polarity	Positive	Set Nebulizer	1.0 Bar
Focus	Not active			Set Dry Heater	180 °C
Scan Begin	50 m/z	Set Capillary	6000 V	Set Dry Gas	4.0 l/min
Scan End	10000 m/z	Set End Plate Offset	-500 V	Set Divert Valve	Source

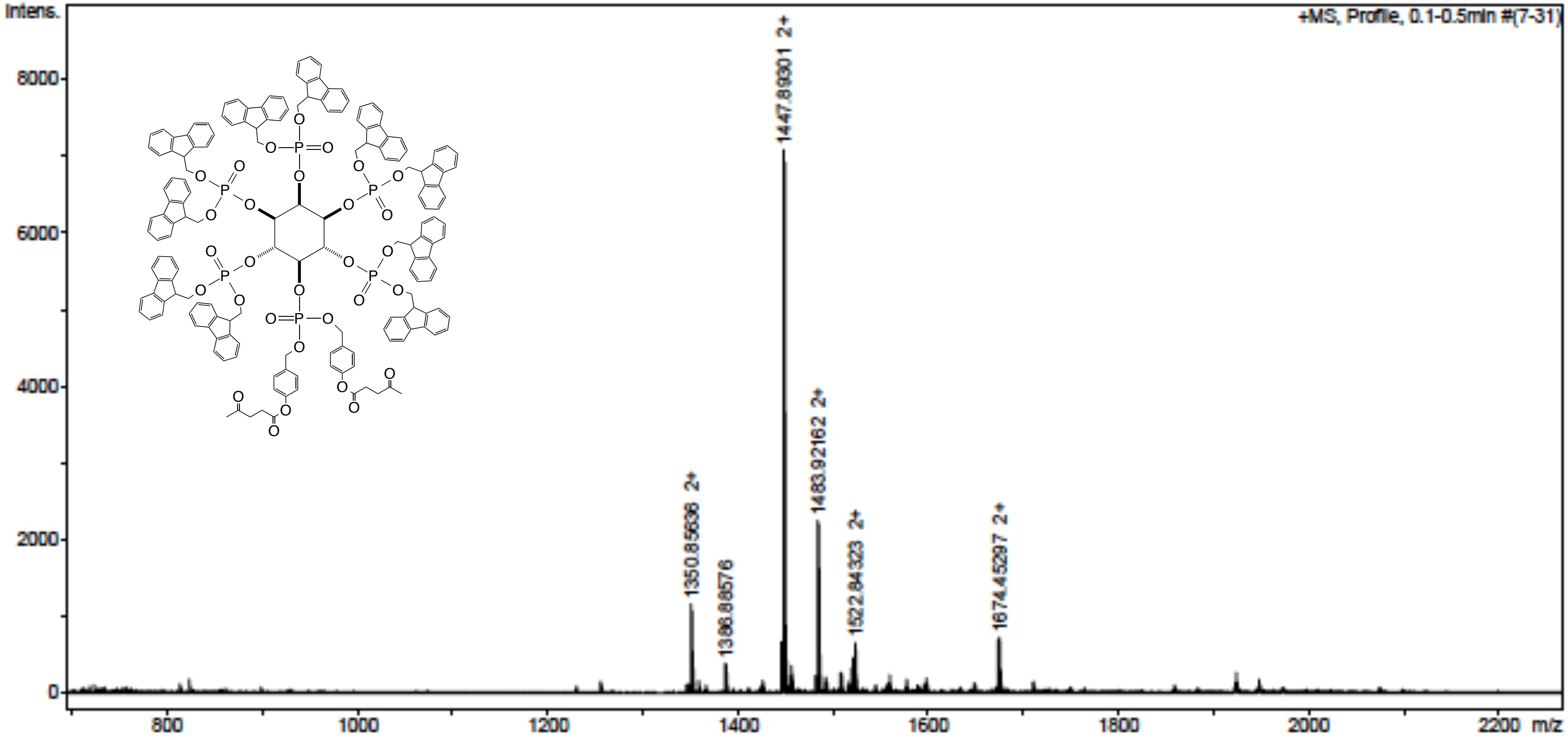

Supplementary Figure 44. ESI MS of compound 6.

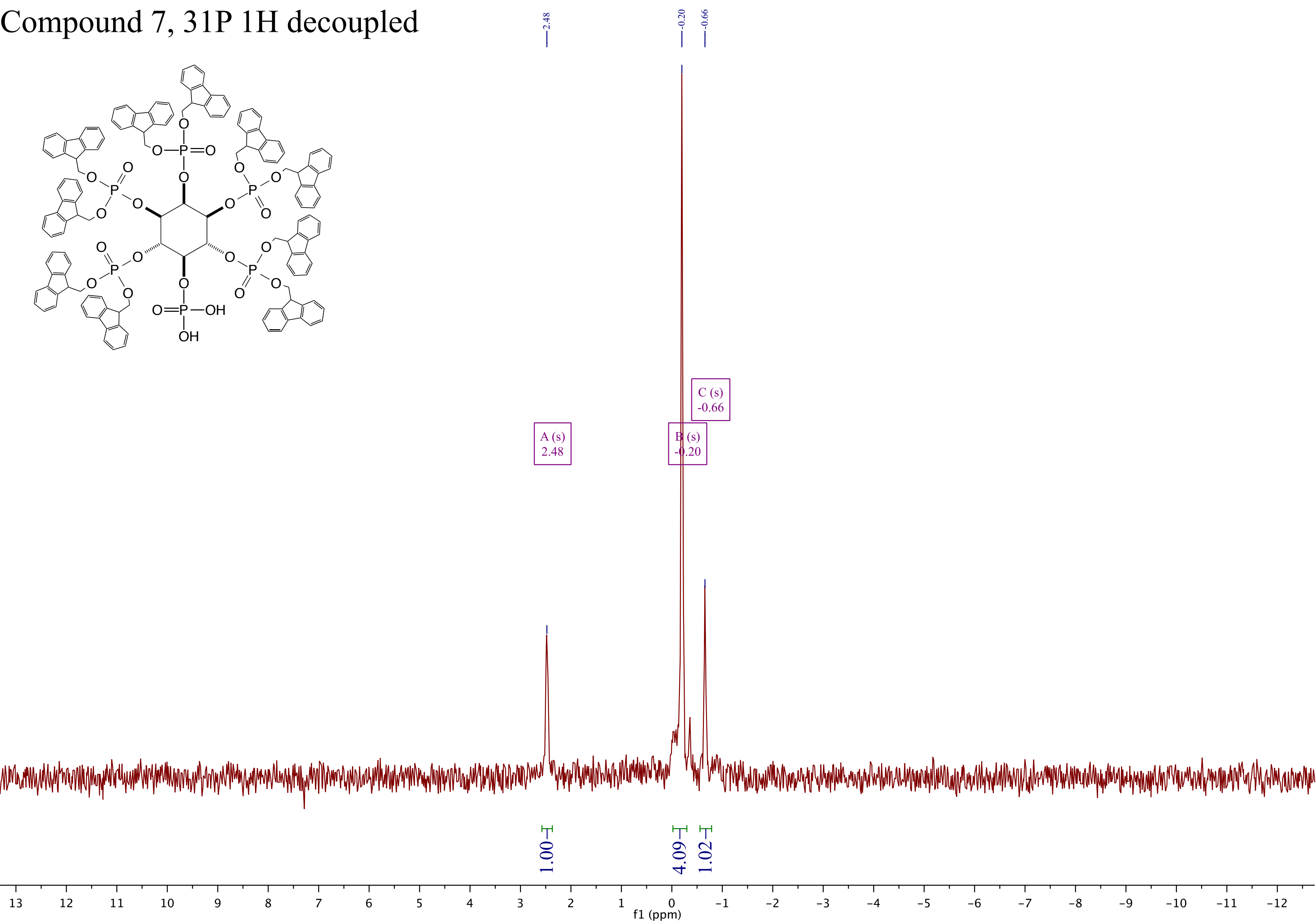

Supplementary Figure 45. 31P NMR of compound 7.

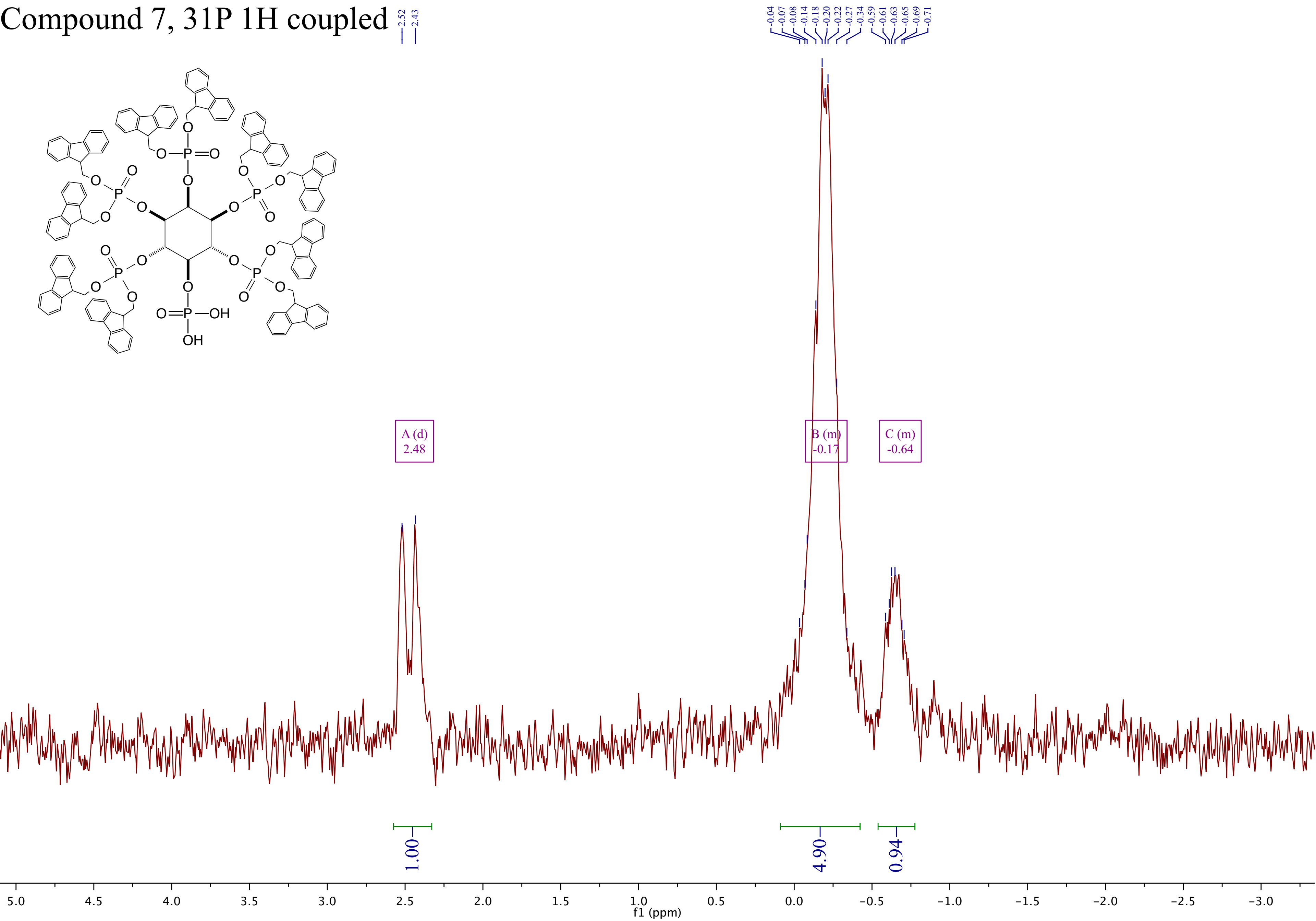

Supplementary Figure 46. 31P NMR 1H coupled of compound 7.

HR-ESI-MS (Bruker maXis)

Analysis Info

Analysis Name	D:\Data\Service\5627sihres.d	Acquisition Date	26.11.2013 12:53:57
Method	ESI_lowMR_TuneMix.m	Operator	ust
Sample Name	IP-4-24P1	Instrument / Ser#	maXis 33
Comment	Solvent: MeOH/CHCl3 3:2 + NaI<br>Client: Pavlovic		

Acquisition Parameter

Source Type	ESI	Ion Polarity	Negative	Set Nebulizer	1.0 Bar
Focus	Not active			Set Dry Heater	180 °C
Scan Begin	50 m/z	Set Capillary	6000 V	Set Dry Gas	4.0 l/min
Scan End	4000 m/z	Set End Plate Offset	-500 V	Set Divert Valve	Source

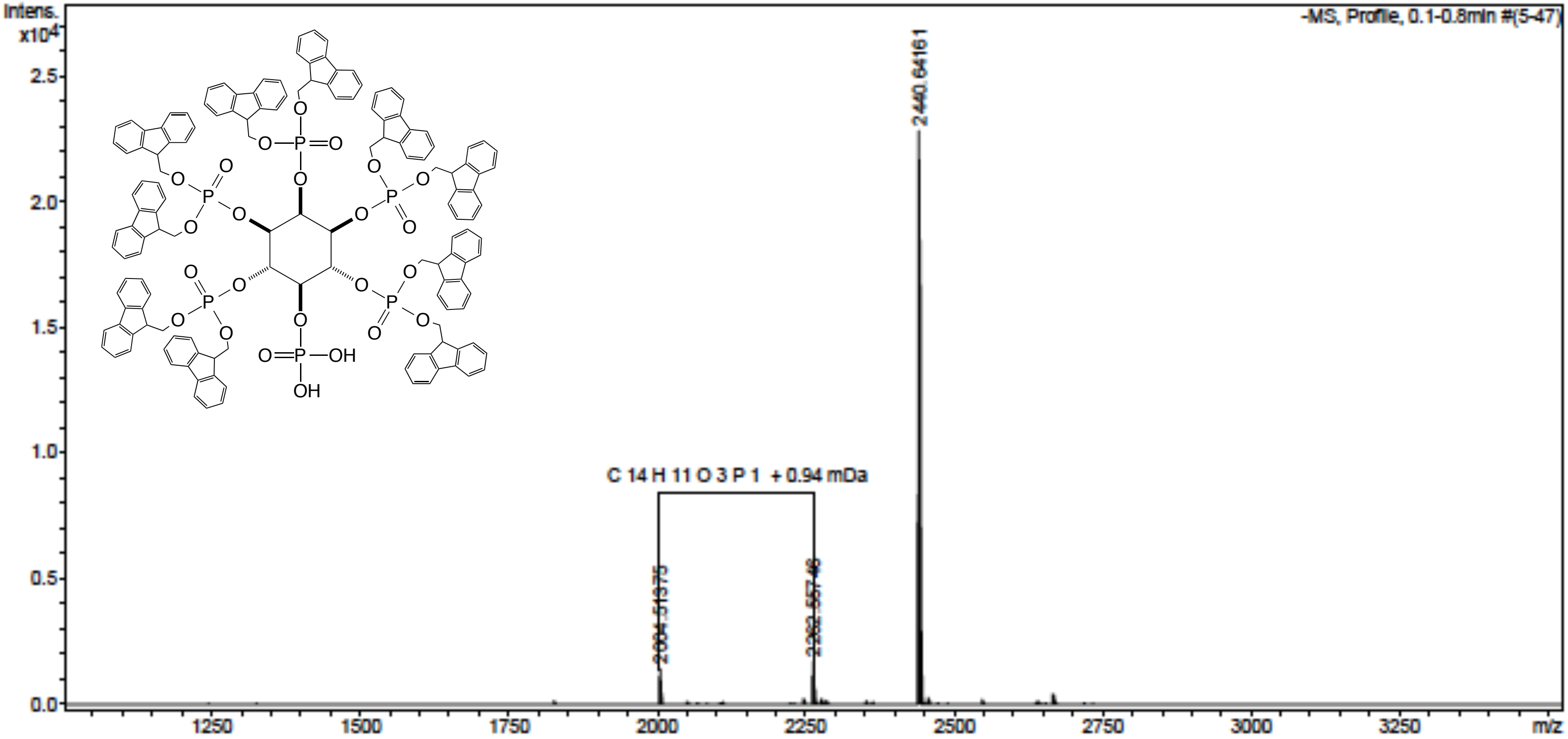

Supplementary Figure 47. ESI MS of compound 7.

Compound 7a, 31P 1H decoupled

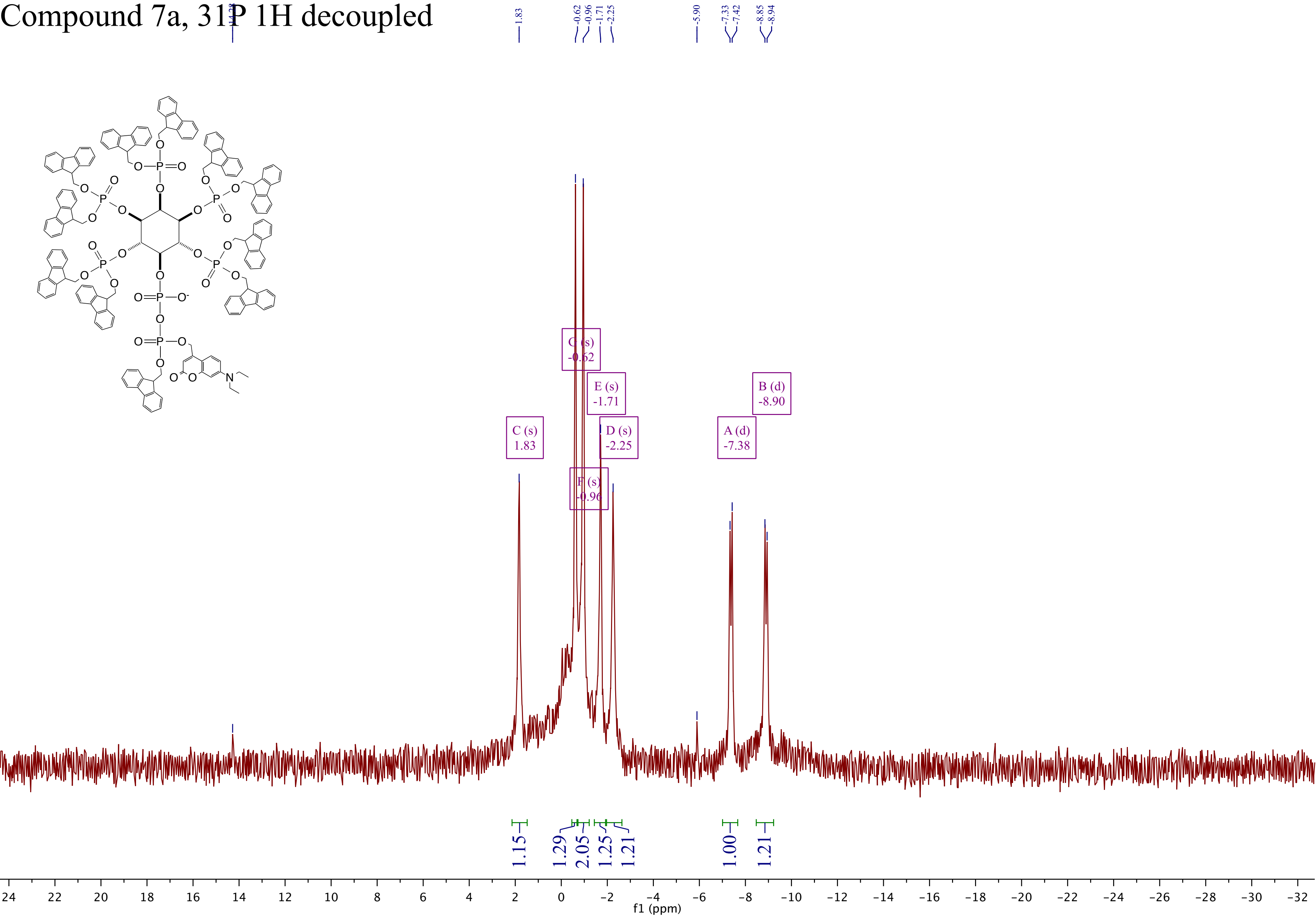

Supplementary Figure 48. 31P NMR of compund 7a.

Compound 7a, 31P 1H coupled

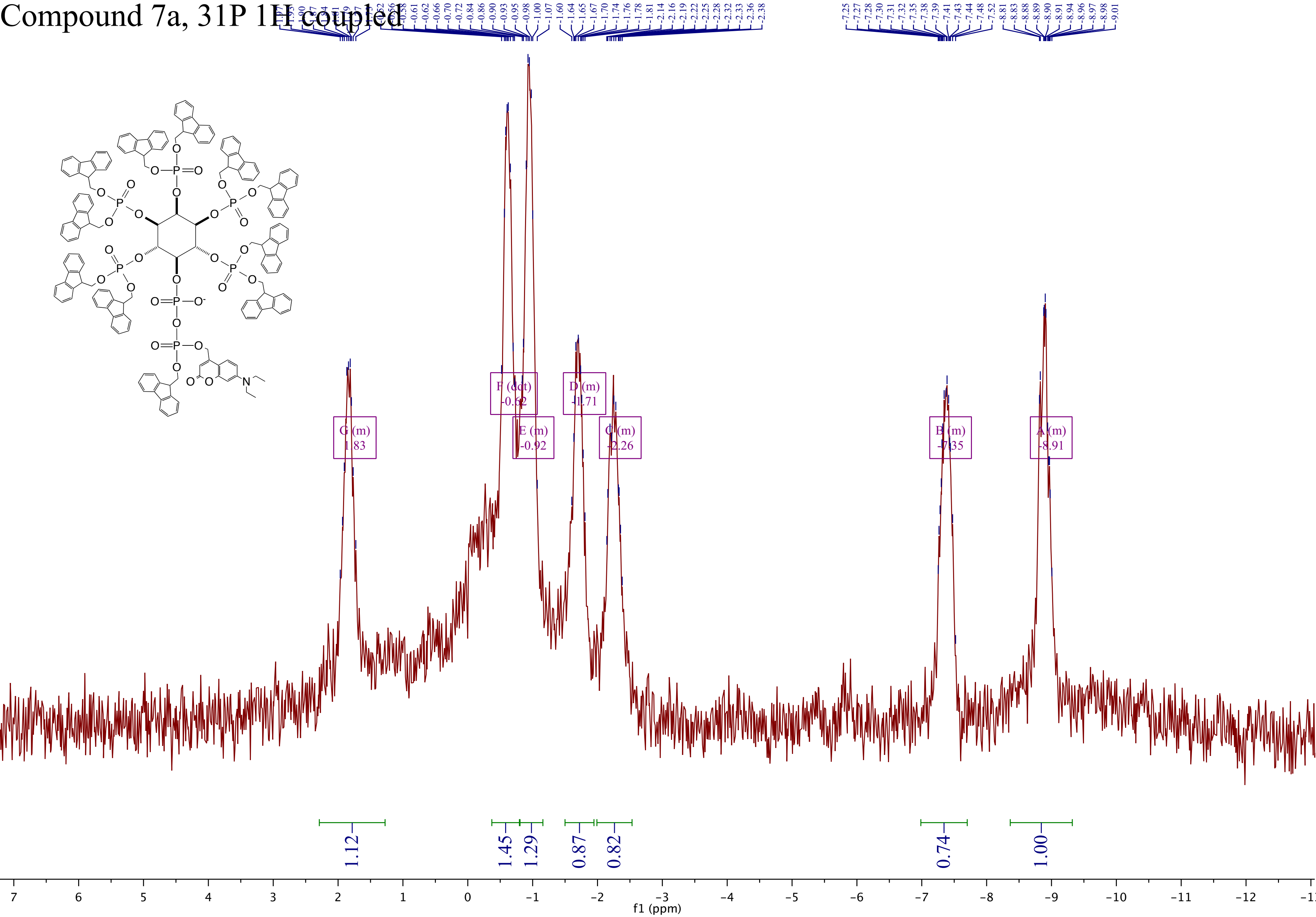

Supplementary Figure 49. 31P NMR 1H coupled of compund 7a.

Compound 7a, 1H

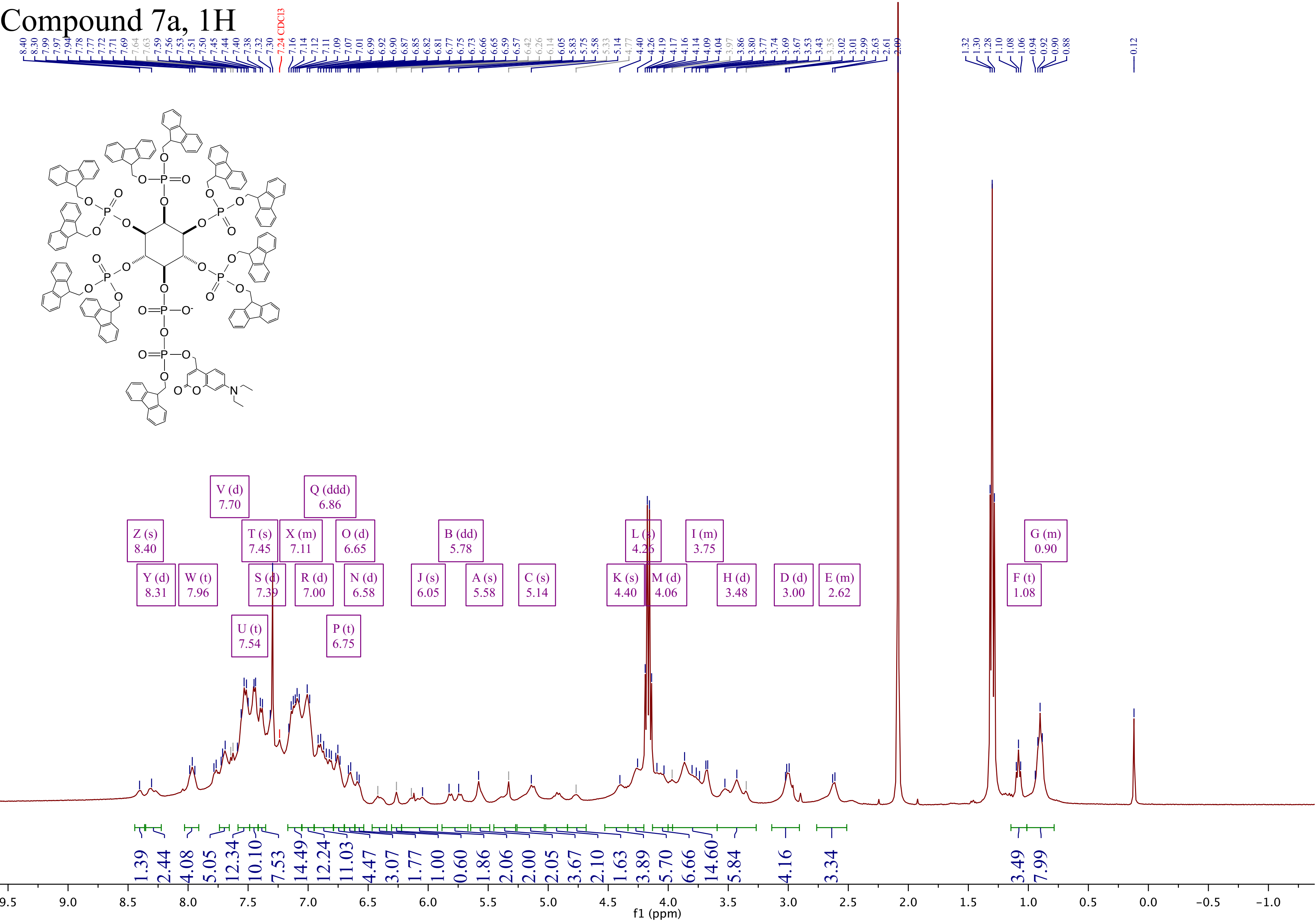

Supplementary Figure 50. 1H NMR of compund 7a.

Compound 7a, 13C

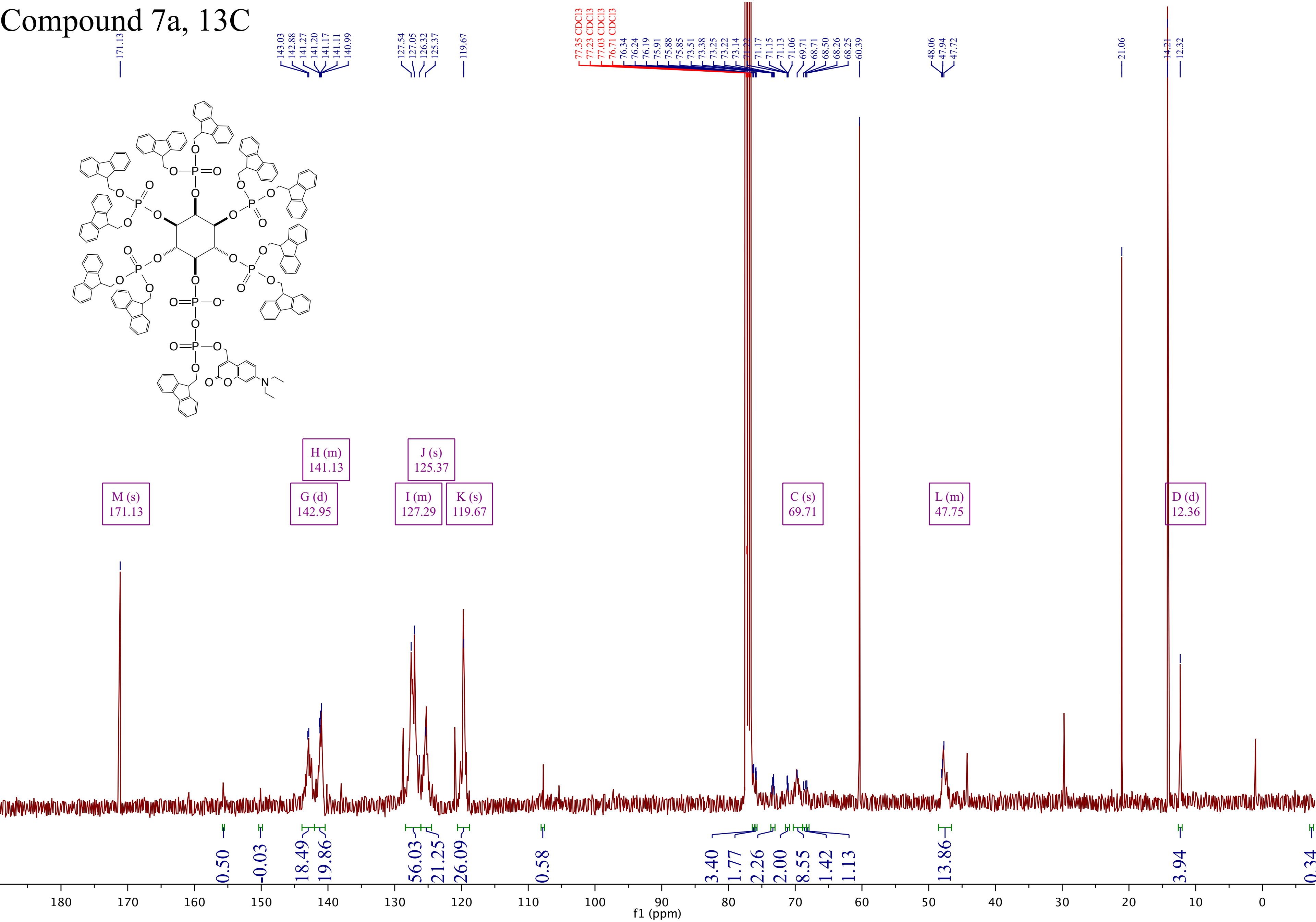

Supplementary Figure 51. 13C NMR of compund 7a.

HR-ESI-MS (Bruker maXis)

Analysis Info

Analysis Name	D:\Data\Service\5659hres.d	Acquisition Date	12/19/2013 3:16:01 PM
Method	ESI_wide_neg.m	Operator	Laurent
Sample Name	IP-4-39C1	Instrument	maXis 255552.00033
Comment	Solvent: MeOH<br>Client: Pavlovic		

Acquisition Parameter

Source Type	ESI	Ion Polarity	Negative	Set Nebulizer	0.2 Bar
Scan Begin	50 m/z	Set Capillary	3000 V	Set Dry Heater	180 °C
Scan End	5000 m/z	Set End Plate Offset	-500 V	Set Dry Gas	4.0 l/min

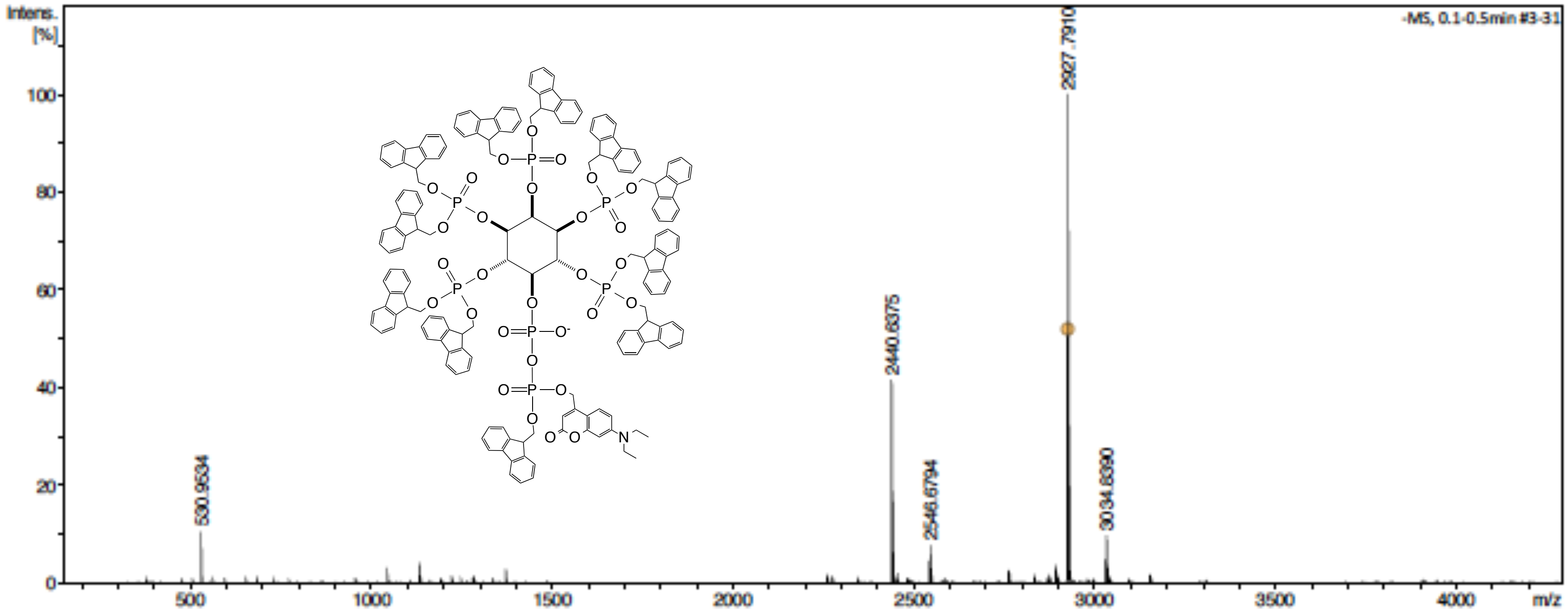

Supplementary Figure 52. ESI MS of compund 7a.

Compound 7b, 31P 1H decoupled

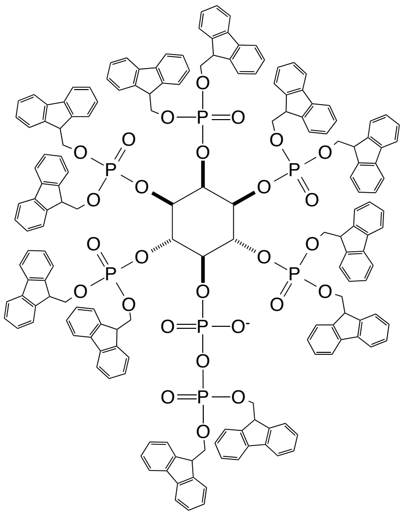

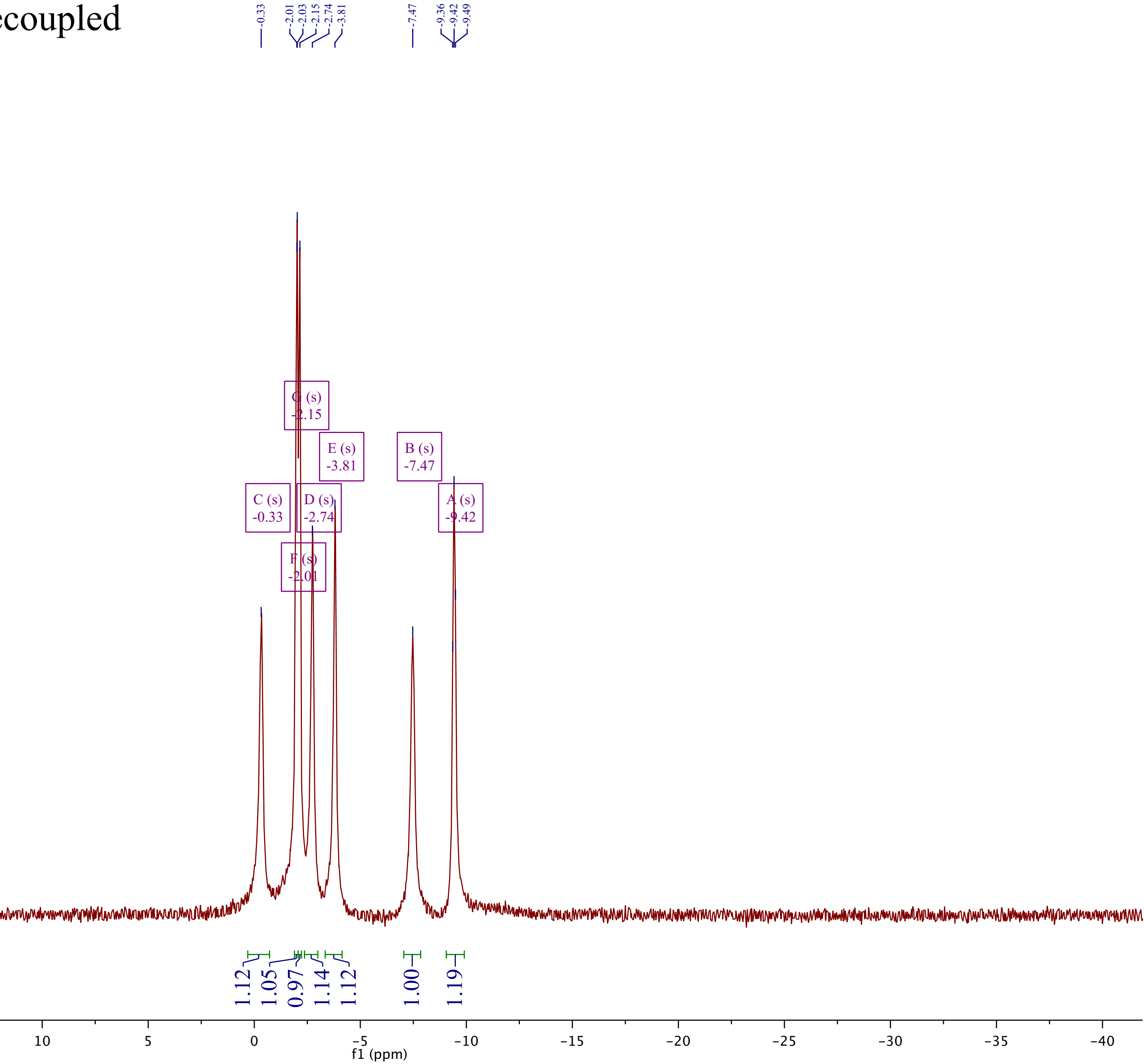

Supplementary Figure 53. 31P NMR of compund 7b.

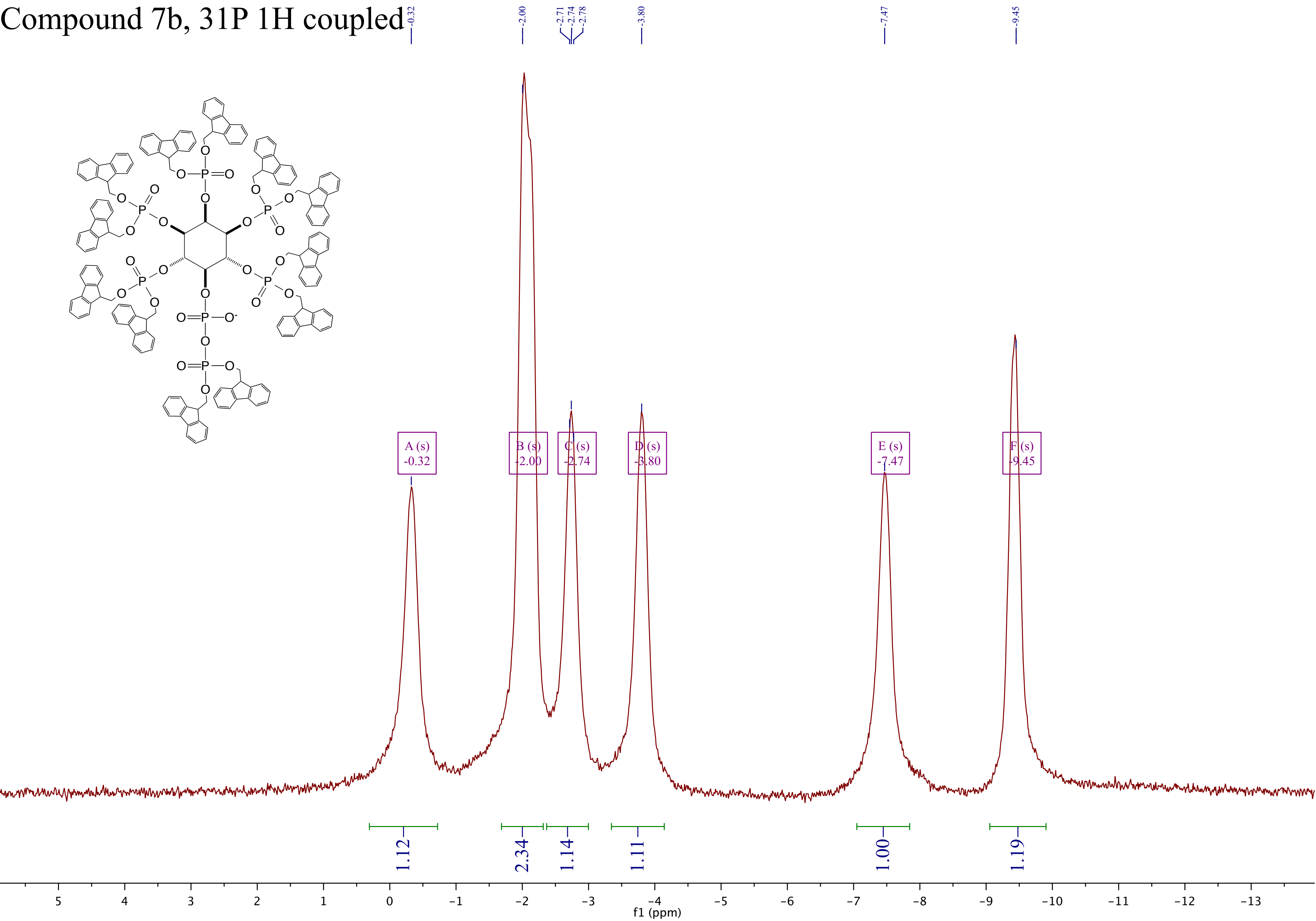

Supplementary Figure 54. 31P NMR 1H coupled of compund 7b.

Compound 7b. 1H

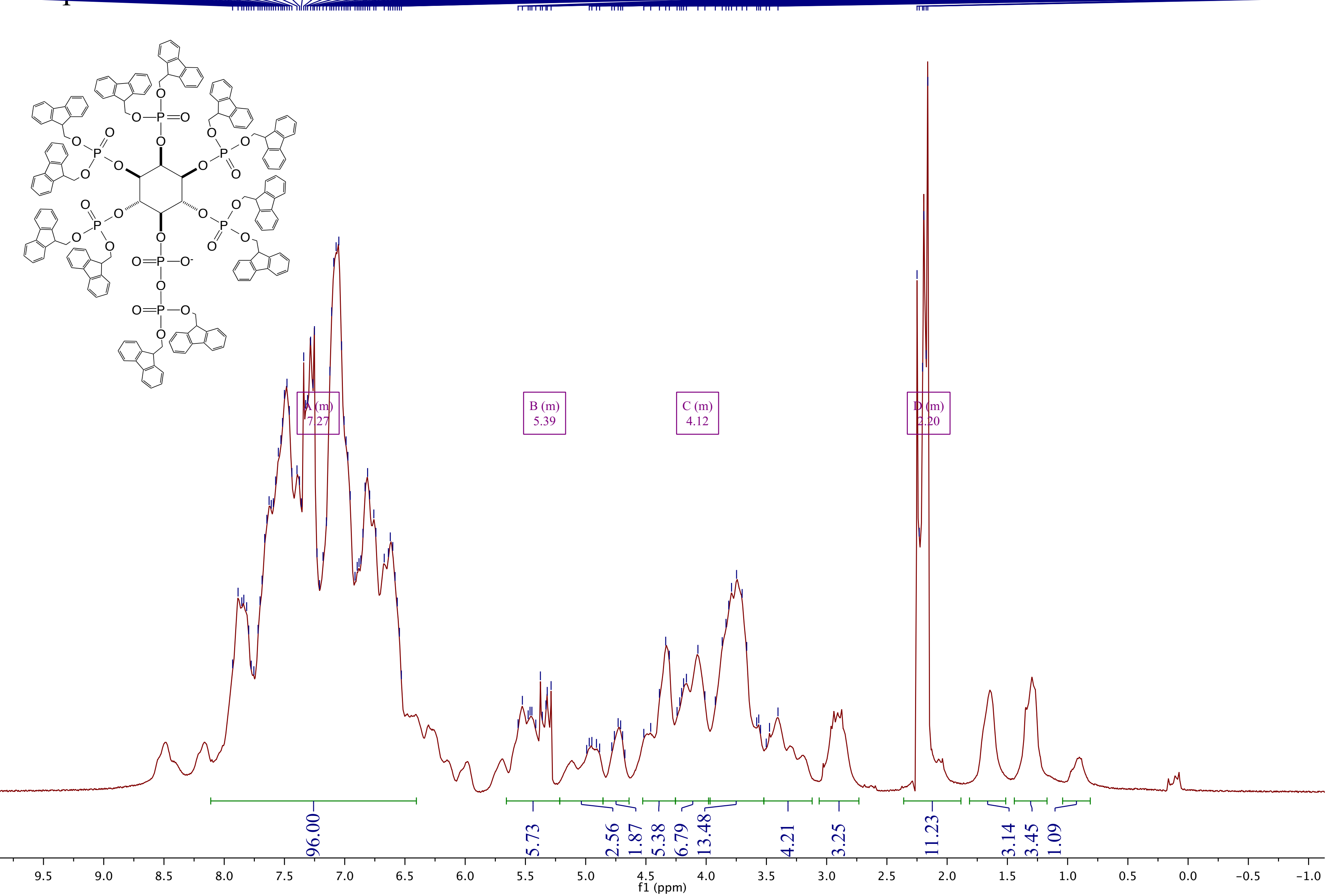

Supplementary Figure 55. 1H NMR of compund 7b.

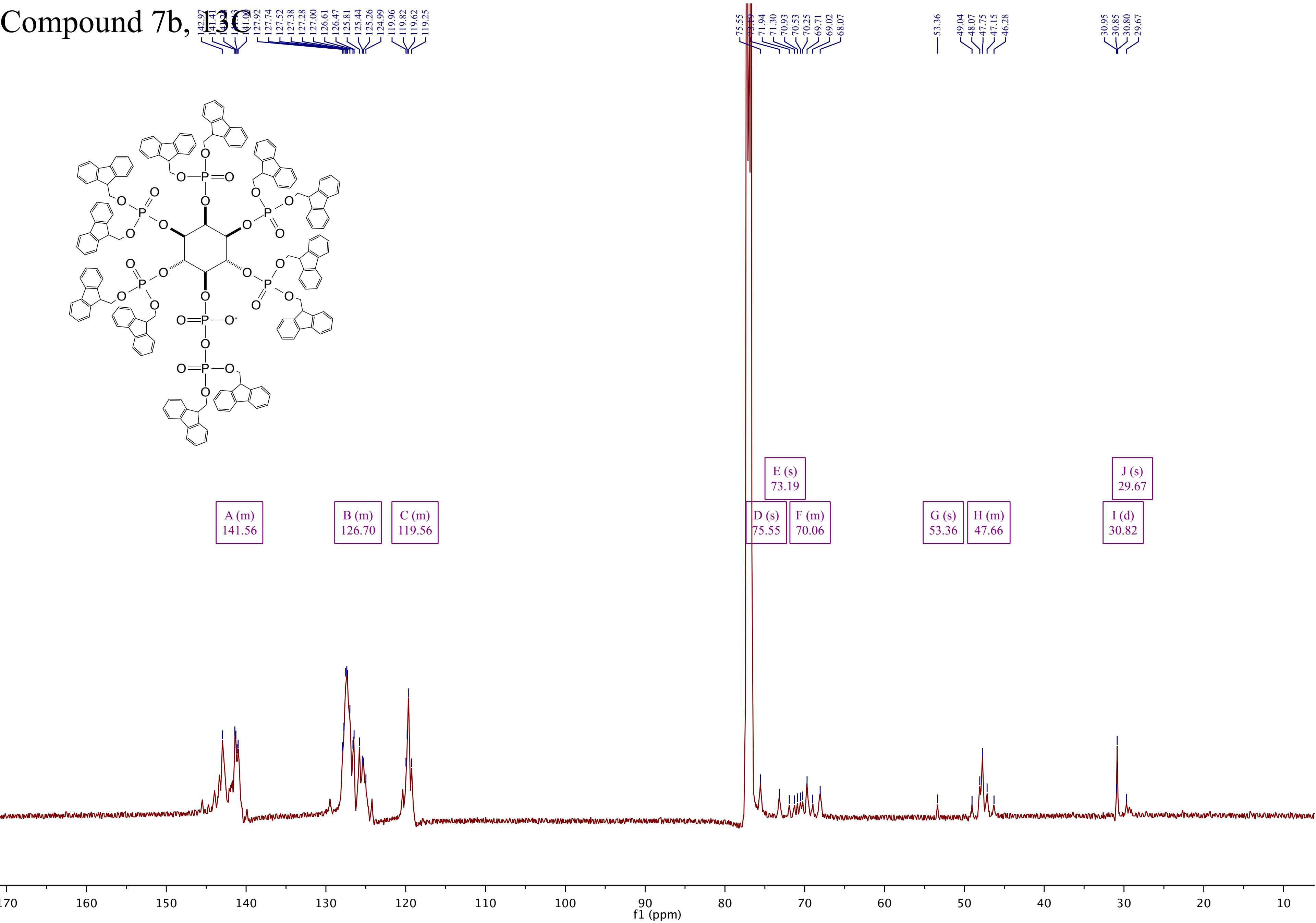

Supplementary Figure 56. 13C NMR of compund 7b.

HR-ESI-MS (Bruker maXis)

Analysis Info

Analysis Name	D:\Data\Service\5701sihres.d	Acquisition Date	1/22/2014 12:36:54 PM
Method	tune_wide_neg.m	Operator	Laurent
Sample Name	IP-4-60P1	Instrument	maXis 255552.00033
Comment	Solvent: MeCN<br>Client: Pavlovic		

Acquisition Parameter

Source Type	ESI	Ion Polarity	Negative	Set Nebulizer	0.4 Bar
Scan Begin	50 m/z	Set Capillary	5000 V	Set Dry Heater	180 °C
Scan End	4000 m/z	Set End Plate Offset	-500 V	Set Dry Gas	4.0 l/min

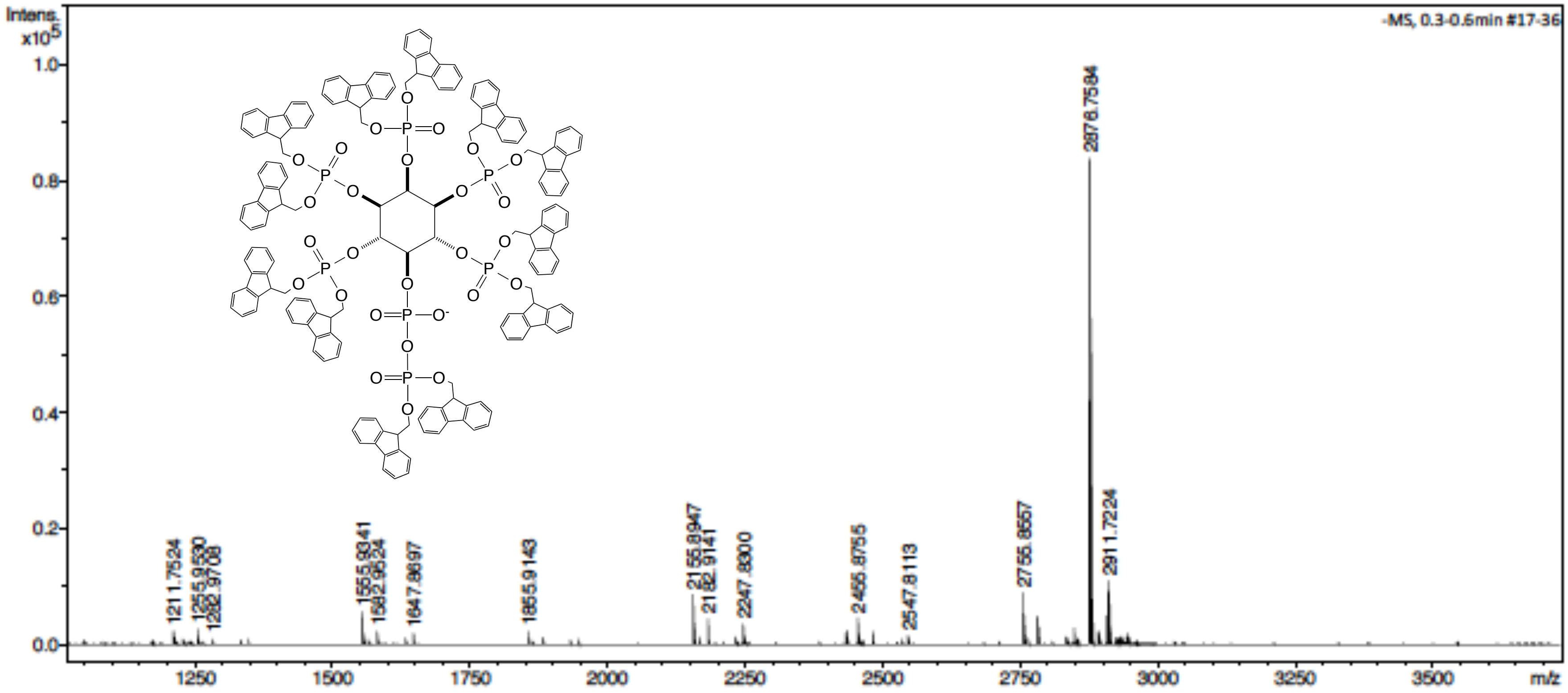

Bruker Compass DataAnalysis 4.1 printed: 1/22/2014 12:46:00 PM by: Laurent Page1 of 2

Supplementary Figure 57. ESI MS of compund 7b.

Compound 9, 31P 1H decoupled

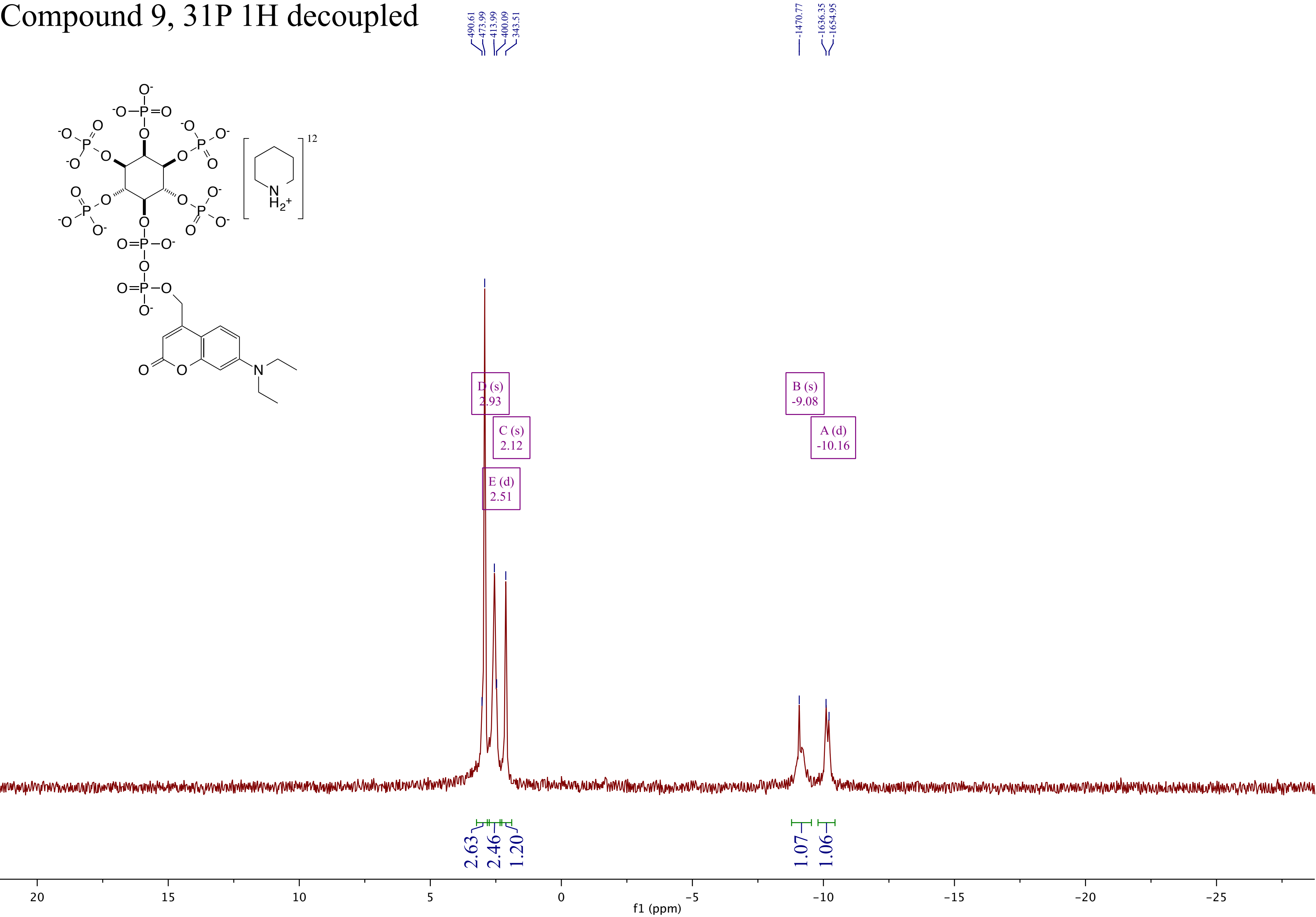

Supplementary Figure 58. 31P NMR of compund 9.

Compound 9, 31P 1H coupled

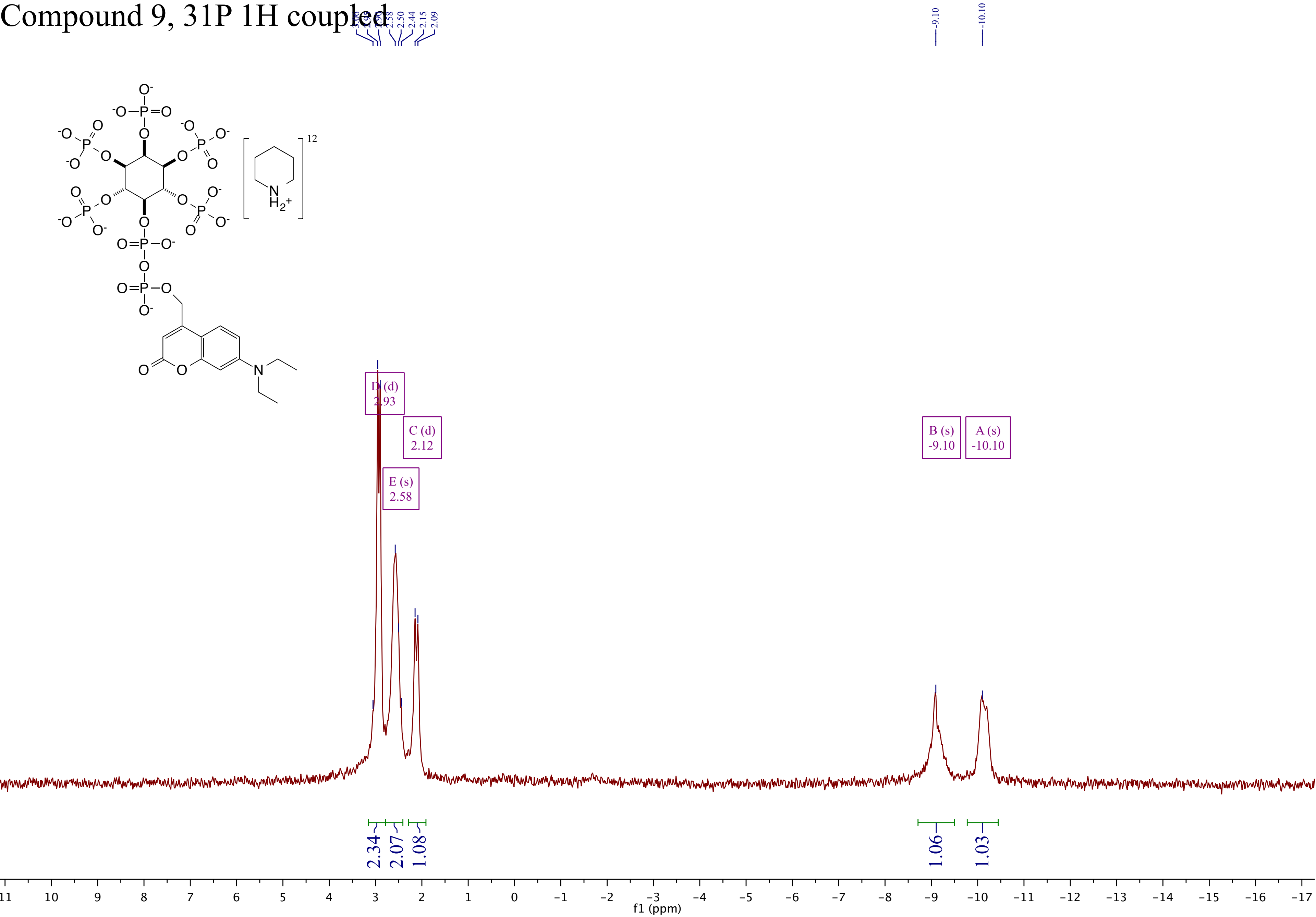

Supplementary Figure 59. 31P NMR 1H coupled of compund 9.

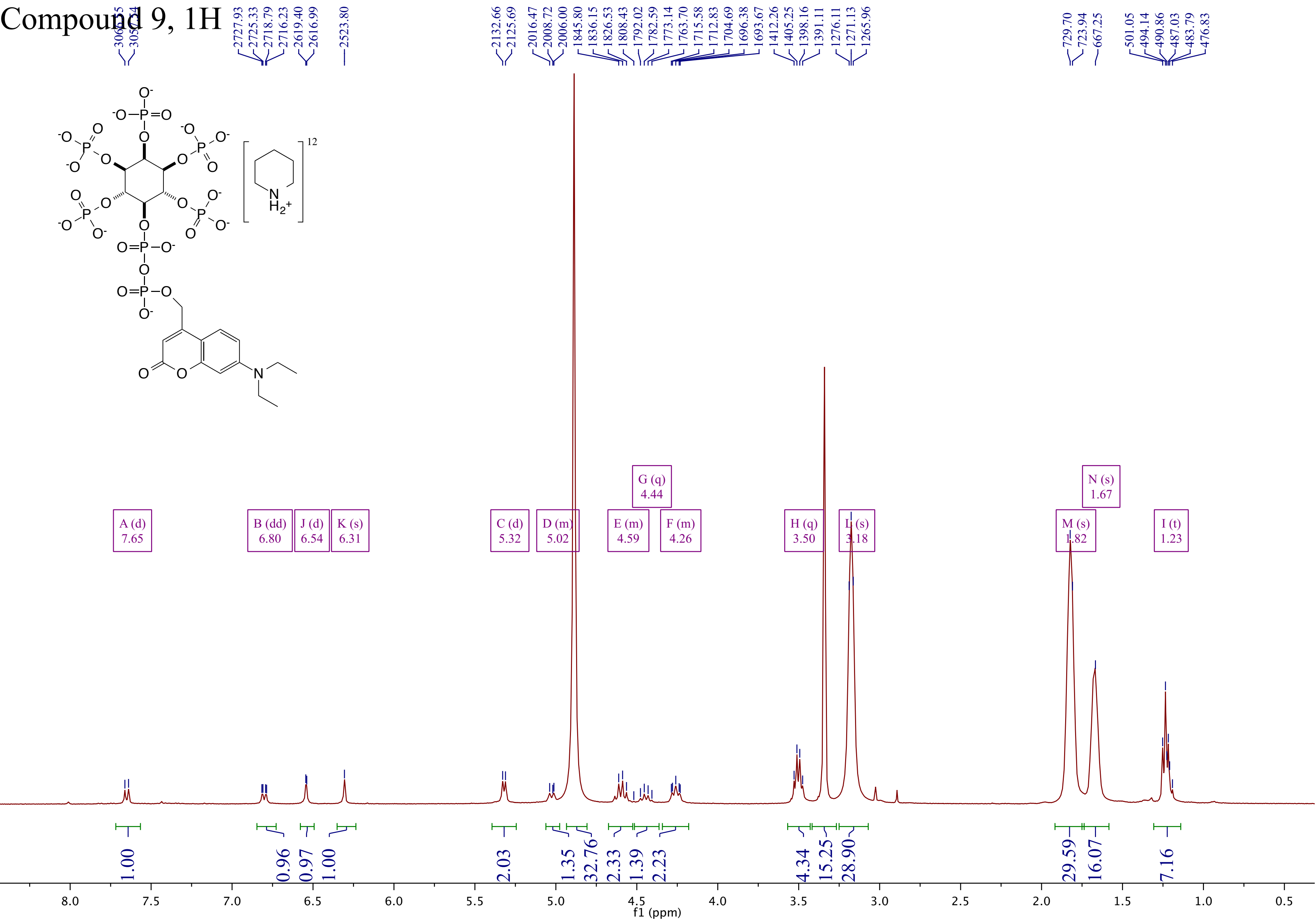

Supplementary Figure 60. 1H NMR of compund 9 as piperidinium salt.

Compound 9, 1H Na salt

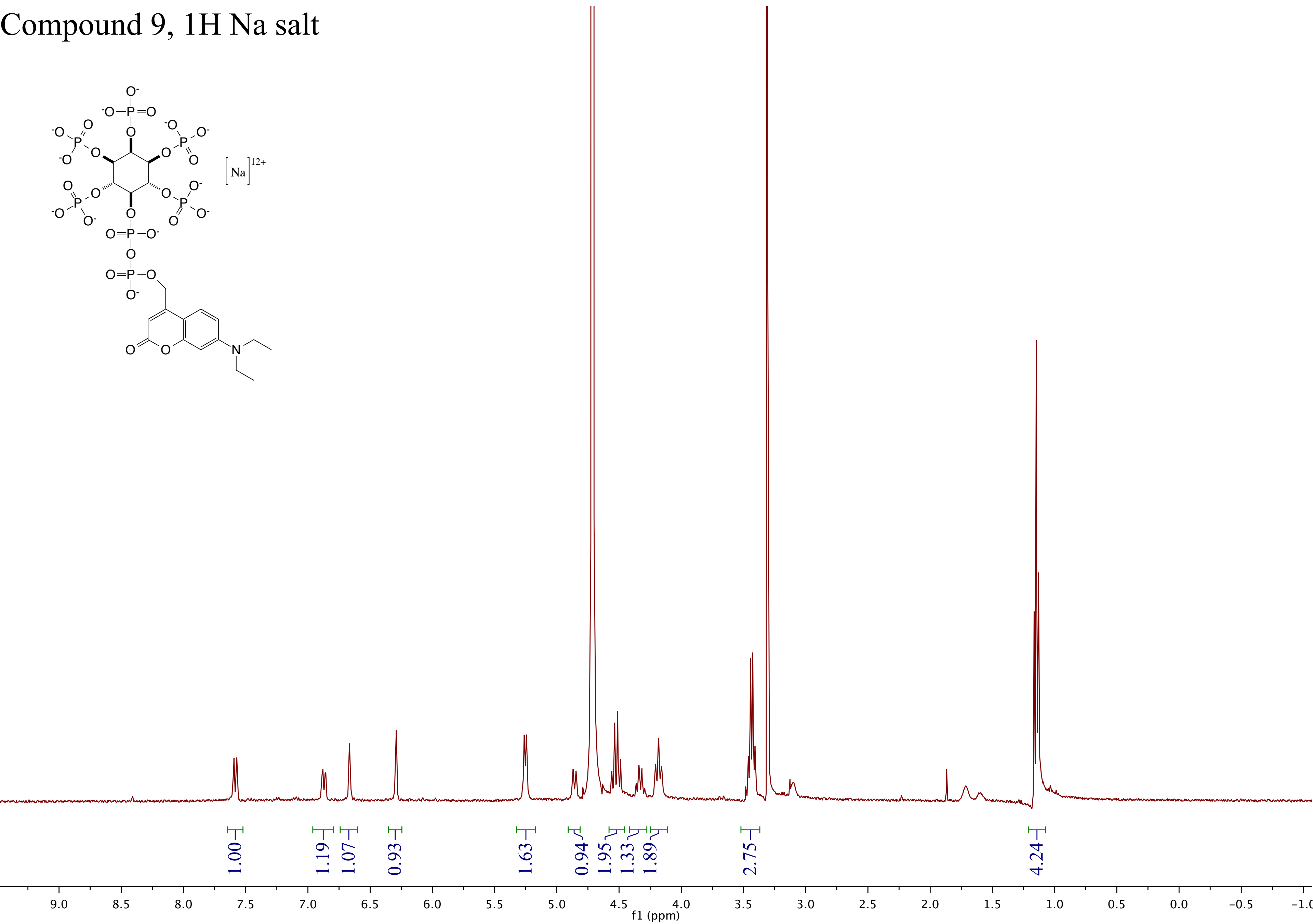

Supplementary Figure 61. 1H NMR of compund 9 as sodium salt.

Compound 9, 13C

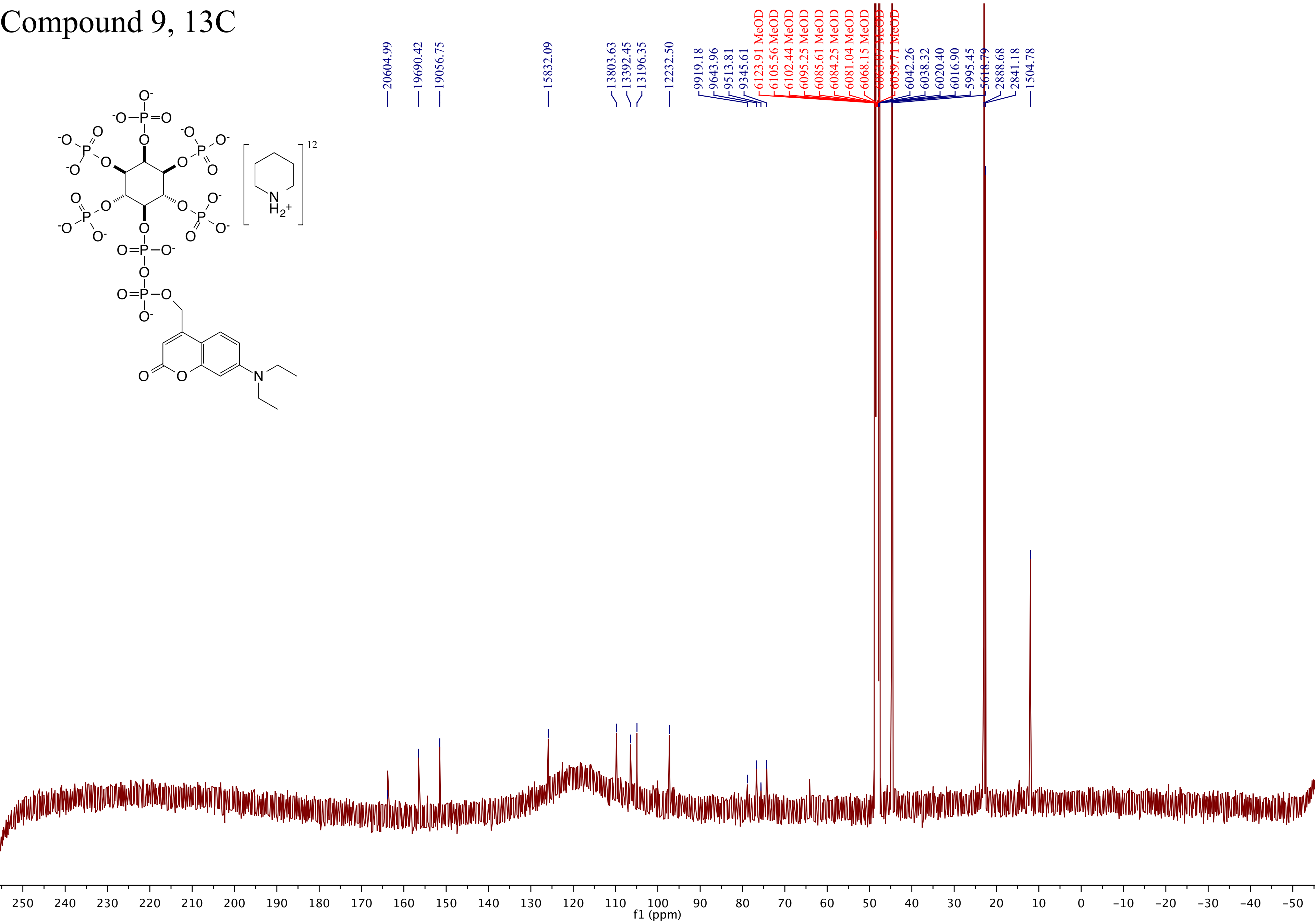

Supplementary Figure 62. 13H NMR of compund 9 as piperidinium salt.

HR-ESI-MS (Bruker maXis)

Analysis Info

Analysis Name	D:\Data\Service\5852sihres.d	Acquisition Date	3/6/2014 8:09:09 AM
Method	tune_low_modified_09_01_14_pos.m	Operator	ust
Sample Name	iP-5-04T1	Instrument	maXis 255552.00033
Comment	Solvent: H2O + NaI<br>Client: Pavlovic		

Acquisition Parameter

Source Type	ESI	Ion Polarity	Positive	Set Nebulizer	0.3 Bar
Scan Begin	50 m/z	Set Capillary	6000 V	Set Dry Heater	180 °C
Scan End	3000 m/z	Set End Plate Offset	-500 V	Set Dry Gas	4.0 l/min

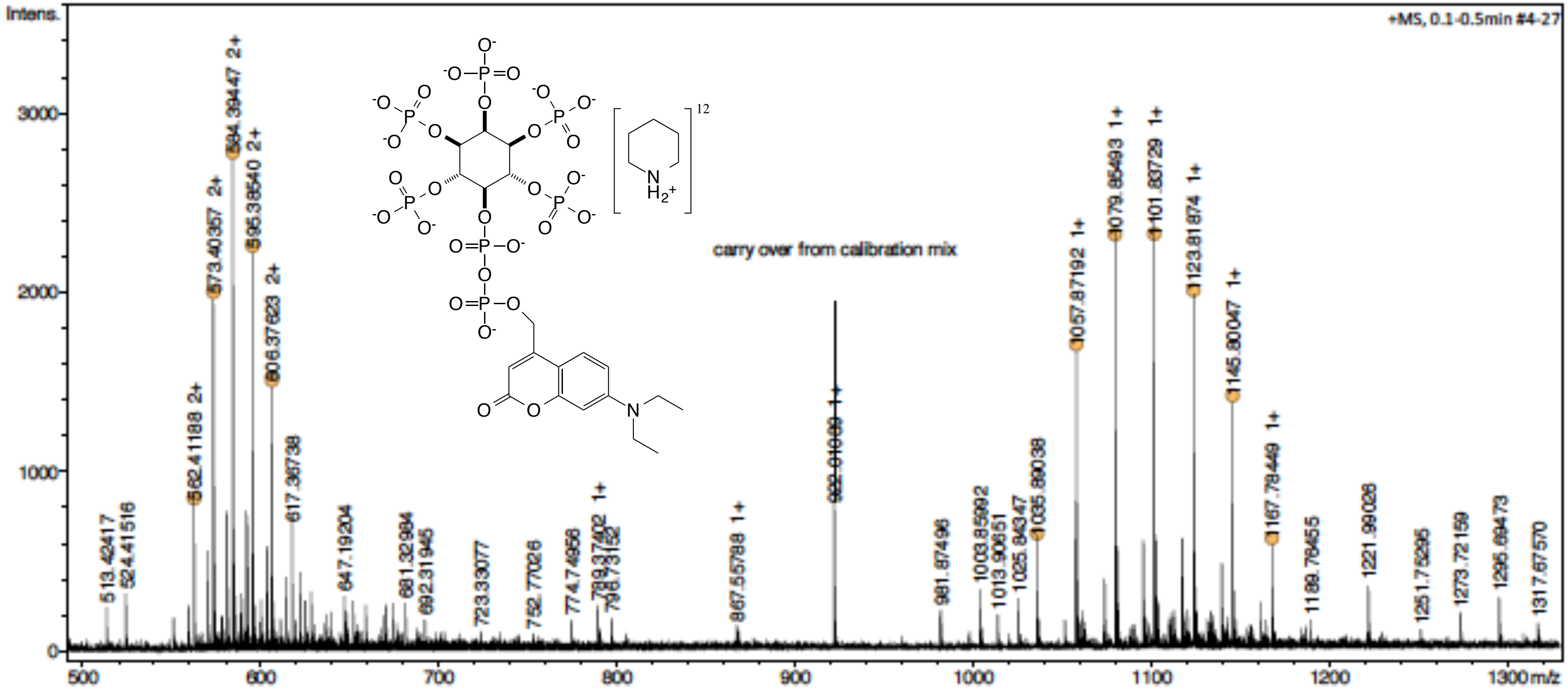

Supplementary Figure 63. ESI MS (positive mode) of compund 9 as piperidinium salt.

HR-ESI-MS (Bruker maXis)

Analysis Info

Analysis Name	D:\Data\Service\5834sihres.d	Acquisition Date	3/3/2014 4:33:06 PM
Method	tune_wide_neg.m	Operator	ust
Sample Name	IP-5-04T1	Instrument	maXis 255552.00033
Comment	Solvent: MeOH<br>Client: Pavlovic		

Acquisition Parameter

Source Type	ESI	Ion Polarity	Negative	Set Nebulizer	0.4 Bar
Scan Begin	50 m/z	Set Capillary	4000 V	Set Dry Heater	180 °C
Scan End	3000 m/z	Set End Plate Offset	-500 V	Set Dry Gas	4.0 l/min

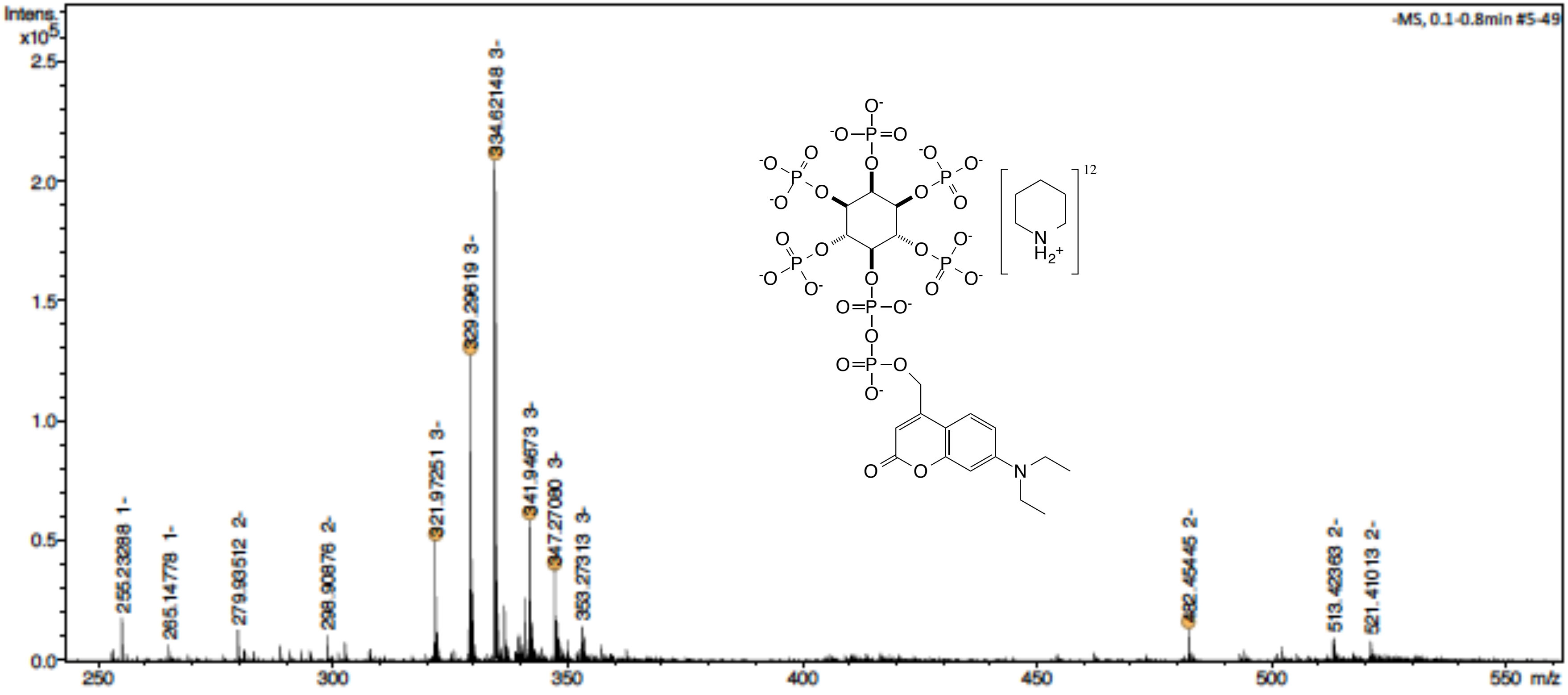

Supplementary Figure 64. ESI MS (negative mode) of compund 9 as piperidinium salt.

Compound 10, 31P 1H decoupled

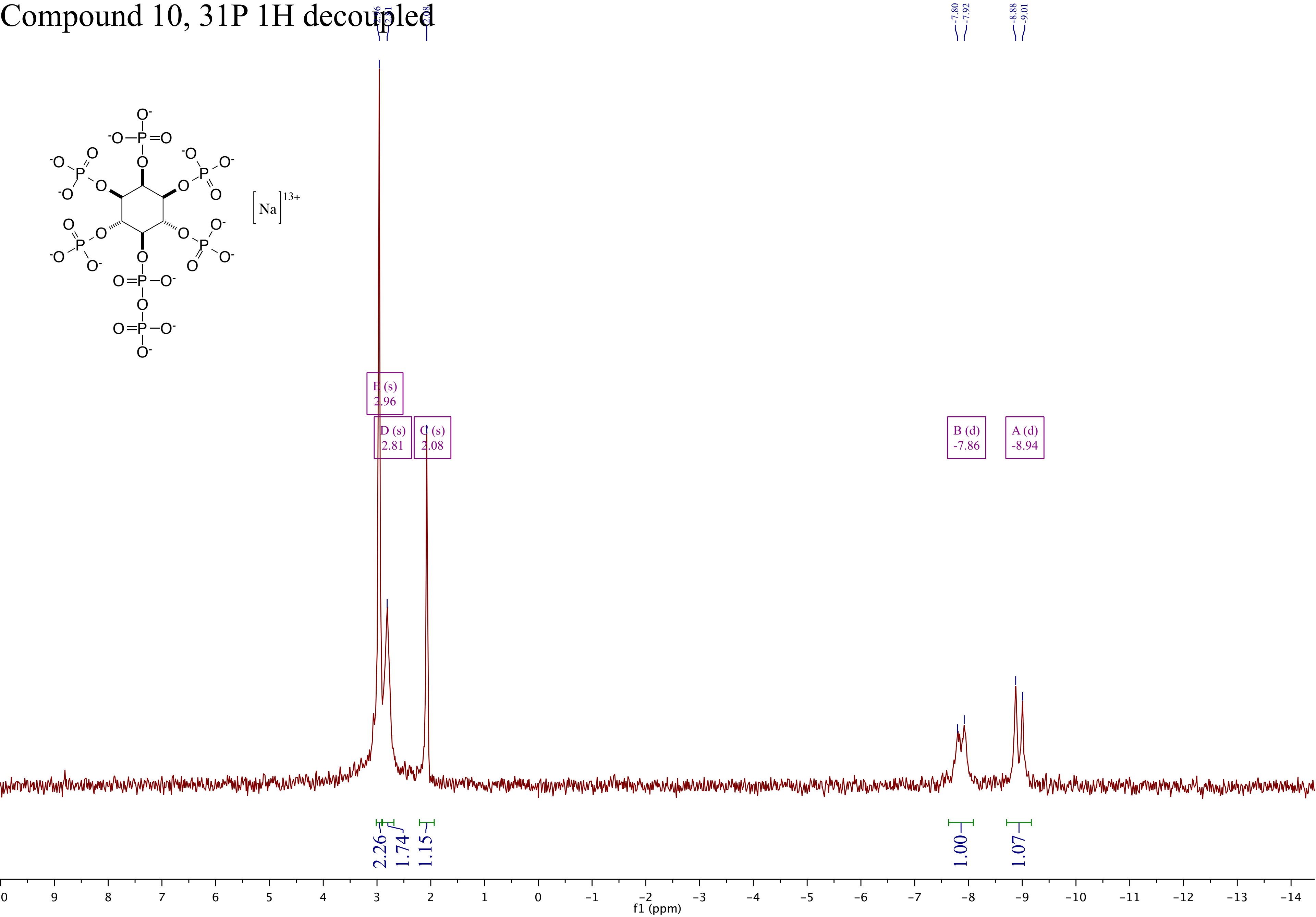

Supplementary Figure 65. 31P NMR of compund 10 as sodium salt.

Compound 10, 31P 1H coupled

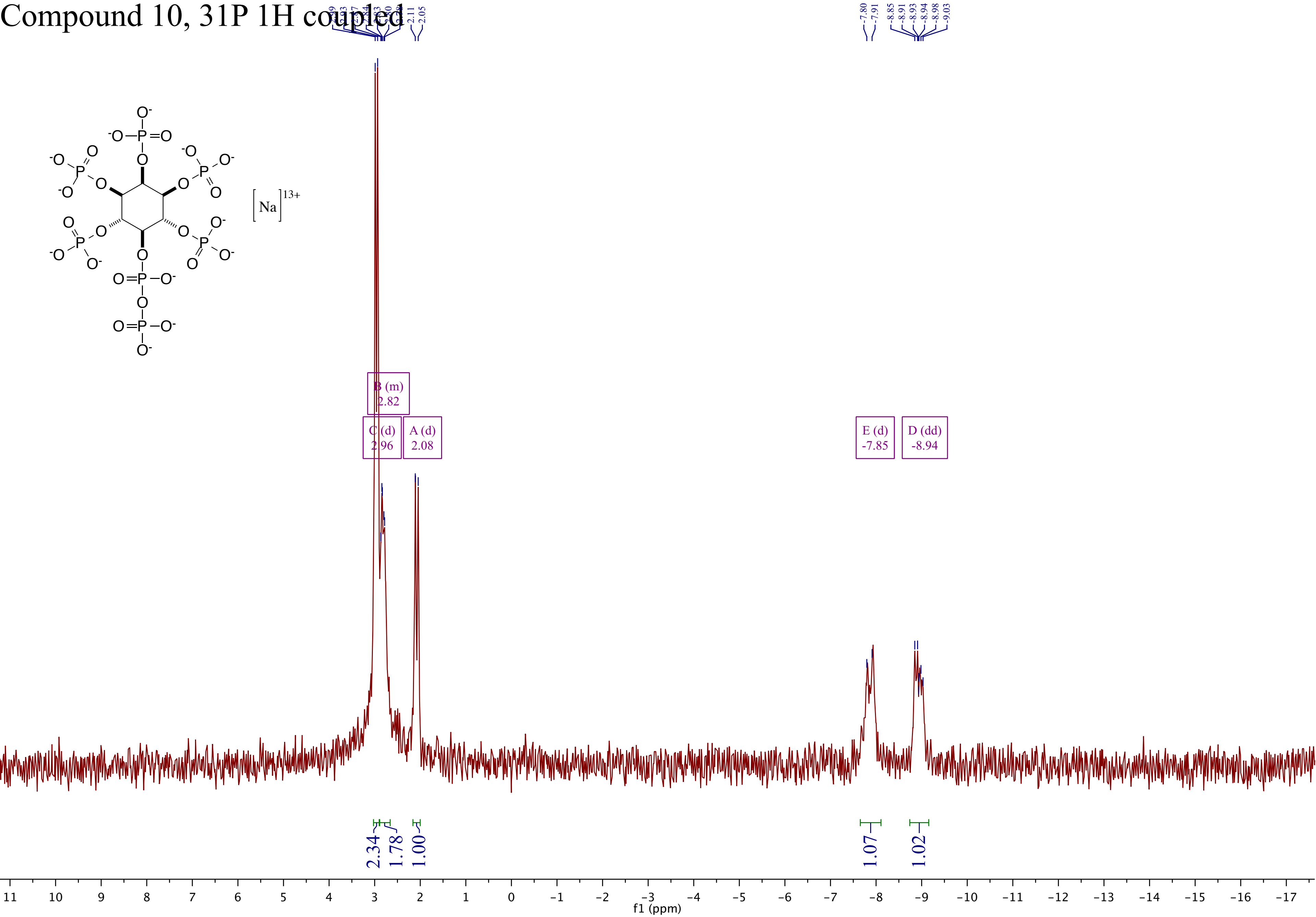

Supplementary Figure 66. 31P NMR 1H coupled of compund 10 as sodium salt.

Compound 10, 1H

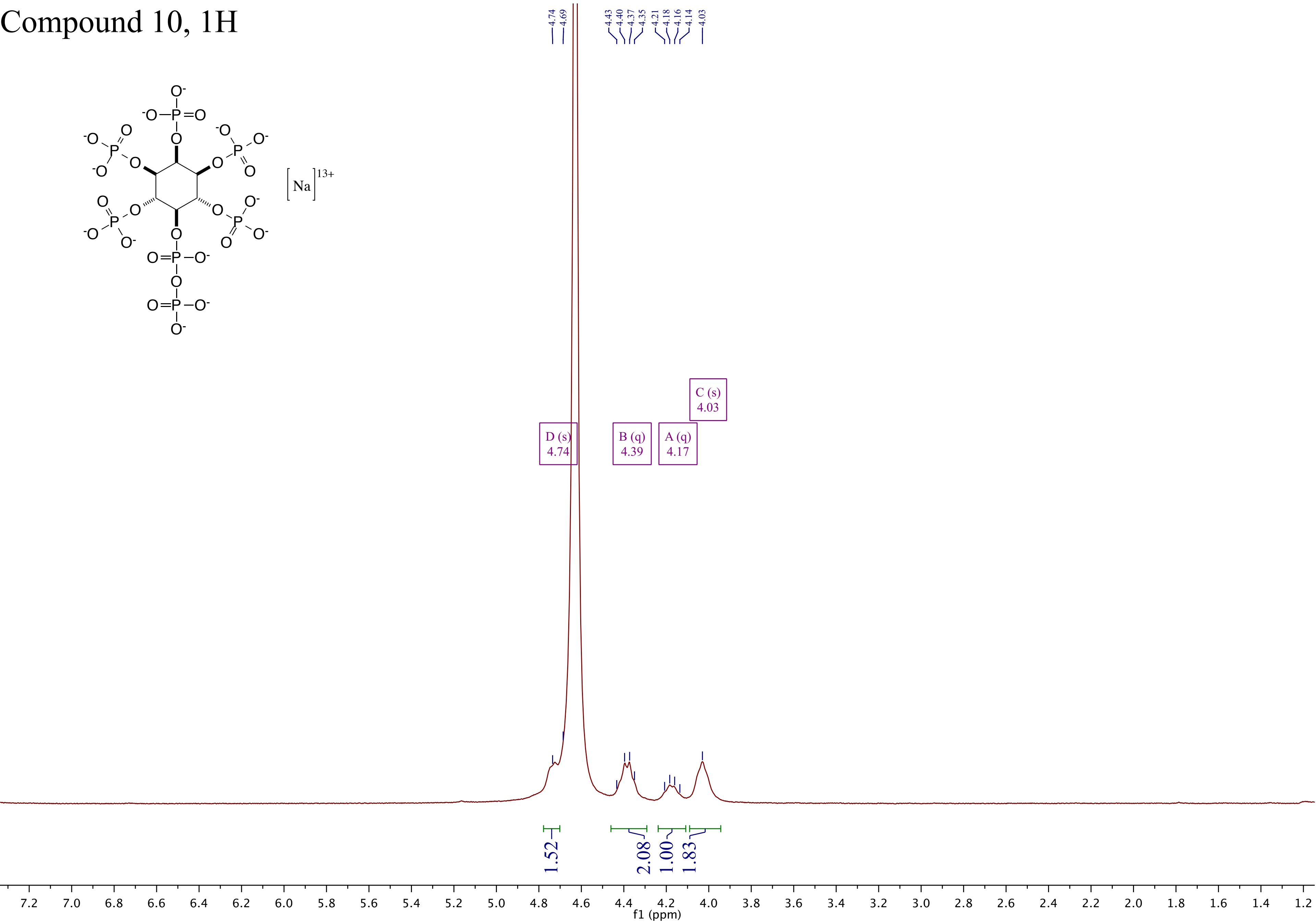

Supplementary Figure 67. 1H NMR of compund 10 as sodium salt.

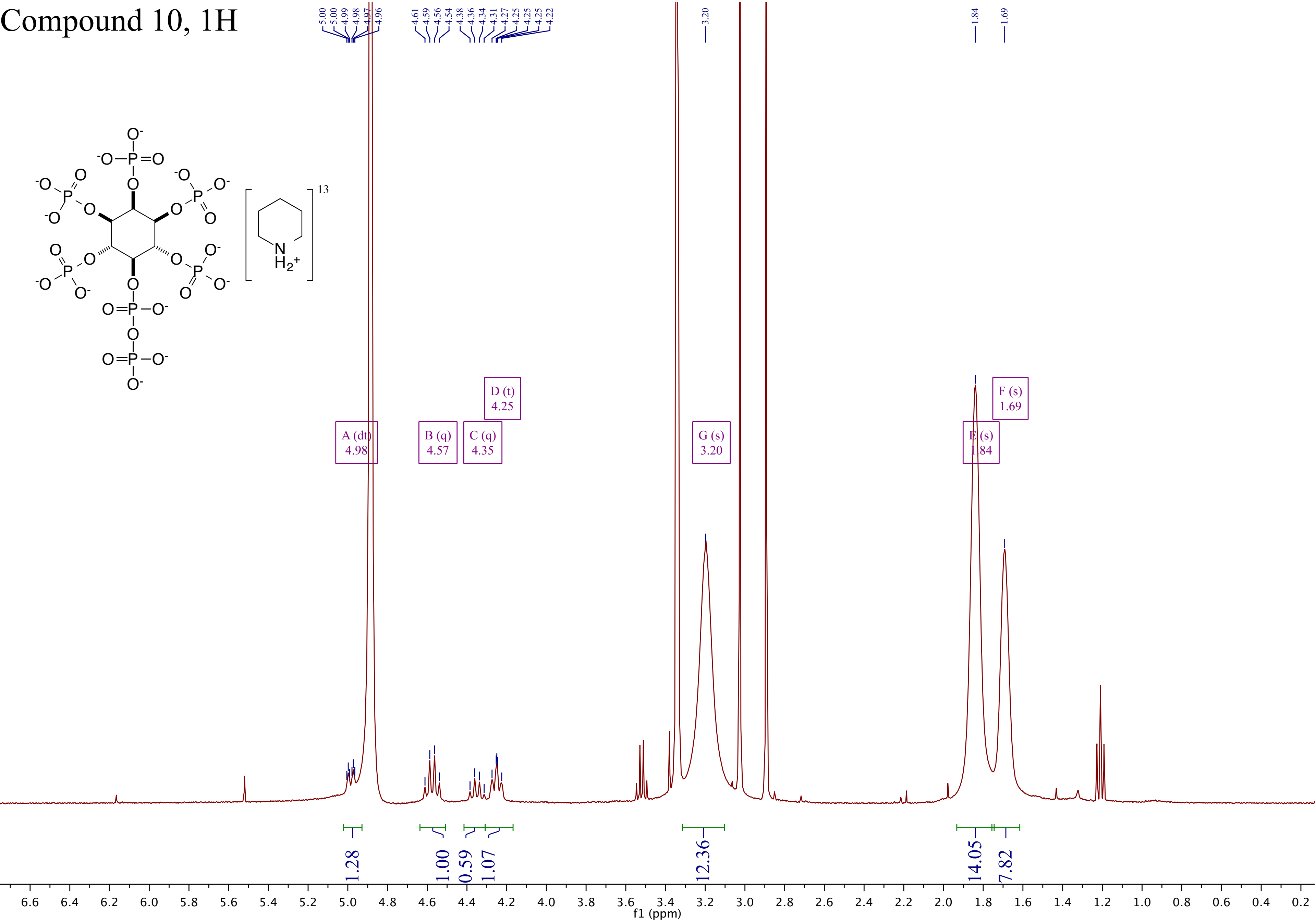

Supplementary Figure 68. 1H NMR of compund 10 as piperidinium salt.

Compound 10, 13C

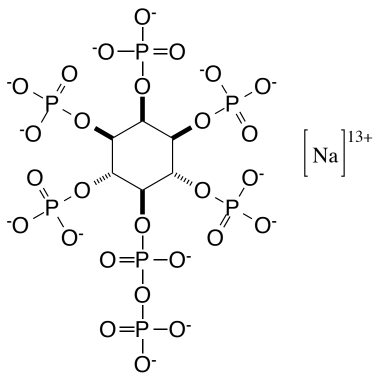

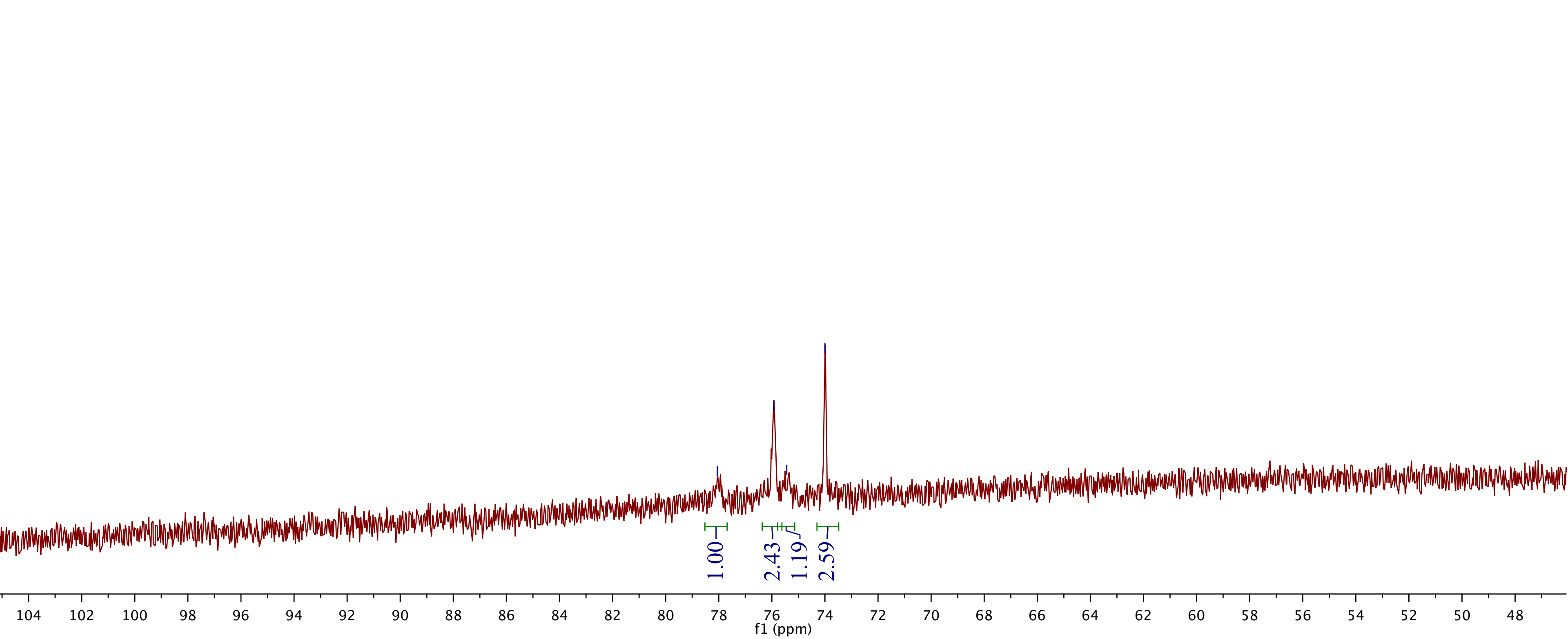

Supplementary Figure 69. 13C NMR of compund 10 as sodium salt.

HR-ESI Report

Analysis Info

Analysis Name	D:\Data\Service\2645sihres.d	Acquisition Date	10/31/2011 9:33:37 AM
Method	ESI_lowMR.m	Operator	ust
Sample Name	HJ-91 (MS/MS 368.90)	Instrument / Ser#	maXis 33
Comment	Solvent: MeOH/H2O 1:1		
	Client: Jessen		

Acquisition Parameter

Source Type	ESI	Ion Polarity	Negative	Set Nebulizer	0.4 Bar
Focus	Not active			Set Dry Heater	180 °C
Scan Begin	100 m/z	Set Capillary	4000 V	Set Dry Gas	4.0 l/min
Scan End	2500 m/z	Set End Plate Offset	-500 V	Set Divert Valve	Source

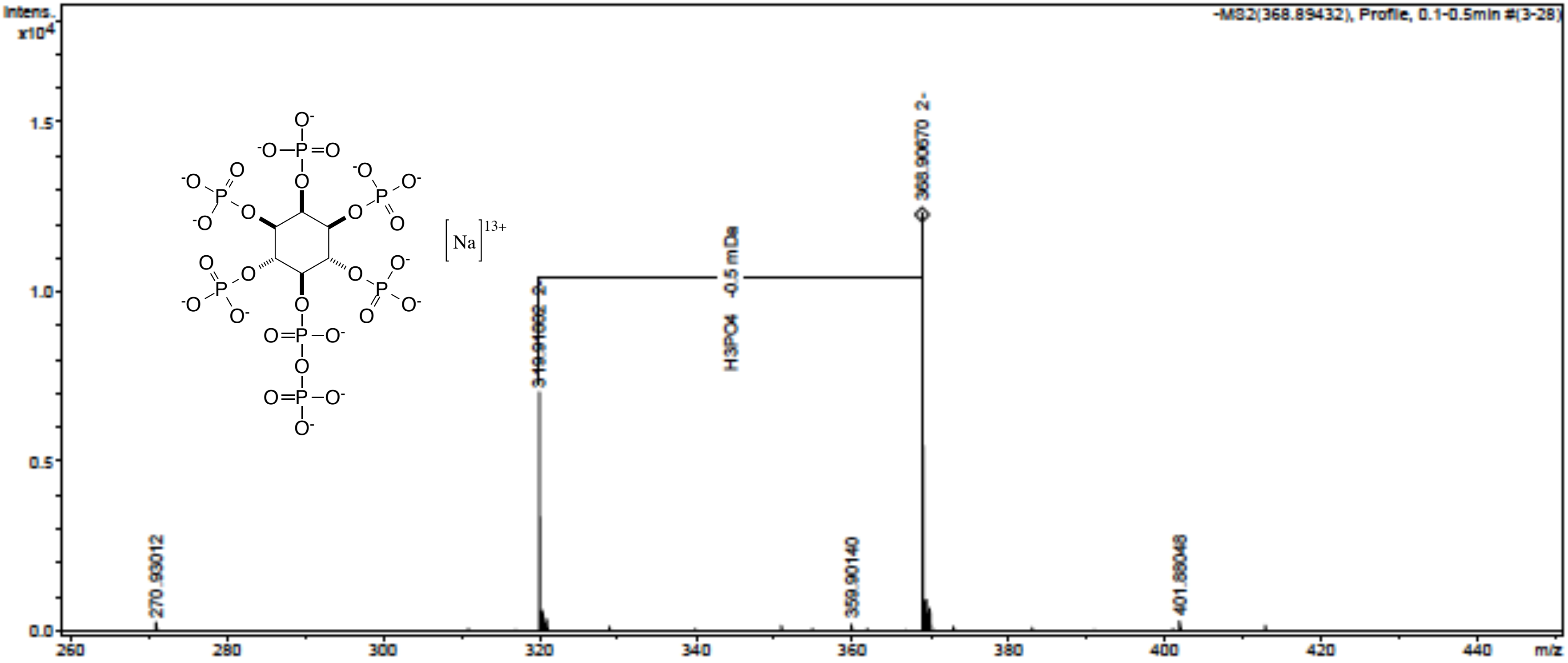

Supplementary Figure 70. ESI MS (negative mode) of compund 10 as sodium salt.

Supplementary Methods

Tissue and cell homogenate preparation.

Tissue homogenate preparation:

Mouse brain and liver tissues were a kind donation from Saiardi Laboratory (Medical Research Council (MRC), Cell Biology Unit and Laboratory for Molecular Cell Biology, University College London) and stored at -80^{0}C.

Brain/liver tissue were thawed on ice. 200 µL tissue lysis buffer were added (50mM TrisHCl (pH = 7.4), 10mM $MgCl_2$, 1mM EGTA and 1 mM DDT). Additionally, 2µL 1X protease inhibitor cocktail (Sigma P8340 500X) were addded. The tissue was gently homogenated by using tissue homogenizer for 15-20 times and incubated on ice for 5min (1 ml tissue grinder, Dounce, Wheaton). Tissue homogenate was pelleted at 14000 rpm, 20min at 4^{0}C and the supernatant transferred into a new Eppendorf tube (repeat 2x). Supernatant was stored at 4^{0}C and protein concentration determined by BCA/ Nano drop (Brain: ≈57µg/ml and Liver: ≈ 71µg/ml).

Cell extract preparation:

Mammalian cell lines HCT116 and MCF7 were grown under standard conditions. For in vitro experiments, mammalian cells were grown up to 80–90% confluence in DMEM media with 10% FCS, 200mM L-glycine, high glucose (4.5g/l) and 5% CO_2 (100mm dishes ≈20 X 10^6 cells). Cells were collected for centrifugation by scraping or adding trypsin. Cells were centrifuged at 2200 rpm for 3–5 min and supernatant was discarded and used for homogenate preparation or stored at -20^{0}C for further experiments. The cells were thawed on ice and 200µl tissue lysis buffer (50mM Tris-HCl (pH7.4), 10mM $MgCl_2$, 1mM EGTA and 1 mM DDT) and 2µl 1X protease inhibitor cocktail (Sigma P8340 500X) were added.

Cells were homogenated by using tissue homogenizer for 15-20 times and incubated on ice for 5min (1ml tissue grinder, Dounce, Wheaton). The cell homogenate was centrifuged at 14000 rpm, 20min at 4^{0}C. The supernatant was transferred into a new Eppendorf cap and the centrigugation process was repeated once more. Supernatant was stored at 4^{0}C and protein concentration determined by BCA/ Nano drop (HCT116, MCF7: ca. 50 µg/ml).

PAGE procedure:

The PAGE procedure was conducted as described previously.[1]

*Photophysical properties of DEACM-5-InsP$_7$ **9**.*

DEACM 5-IP$_7$ **9** (c=10 μM) properties:

l_{abs}^{max} = 386 nm; maximum of the absorption spectra

ε^{max} = 11634 $M^{-1}cm^{-1}$; extinction coefficient in the absorption maximum

φ_{chem} = 1.8 %; quantum yield for the disappearance of the caged compound

l_{f}^{max} = 500 nm; maximum in the fluorescence spectra

φ_{f} = 6.2 %; fluorescence quantum yield

τ_{f} = 1.21 ns; fluorescence life time.

All optical measurements were performed in HEPES (4-(2-hydroxyethyl)-1-piperazineethanesulfonic acid) buffer 0.01mM, pH=7.3 (pH adjusted with NaOH). The 10 μM solutions of the compound were placed in quartz cuvettes with path length of 1 cm. Fluorescence measurements were carried on Edinburgh FLS920 spectrophotometer, using 450W Xenon lamp, with excitation and emission slit widths at 1 nm. Emission spectra were obtained by exciting at the longest wavelength excitation maximum. Fluorescence quantum yield was measured using an integrating sphere accessory, and lifetime using colloidal silica as a blank solution.

UV-Vis measurements were carried out on a Agilent 8453 UV-Vis spectrophotometer. Six independent solutions of varying concentration were analyzed and the extinction coefficient was calculated by linear least-squares fitting of plots of A vs. concentration.

Determination of the quantum yield of uncaging of **9**: Solutions of *trans*-azobenzene in methanol and of **9** in HEPES buffer (pH=7.3) were diluted to identical optical densities at 355 nm of 0.19. 120 μL of either solution was irradiated for certain irradiation intervals with an Edinburgh Instruments LP920 laser flash photolysis setup with a Continuum Surelite Nd:YAG laser, frequency-tripled to generate monochromatic (*i.e.* bandwidth of less than 1 nm) light with a wavelength of 355 nm.

The laser was slightly misaligned in order to reach a suitable irradiation power. The progress of the photoisomerization of azobenzene was monitored by UV/Vis absorbance spectroscopy, determining the absorbance at 355 nm. The progress of the uncaging reaction of caged IP7 was monitored by analytical Varian Pro Star HPLC and a C-18 reverse-phase column (XBridge C18 3.5 μm, 150 x 4.6 mm) using a 0 – 100 % gradient of CH_3CN in 0.1 M TEAA buffer (pH = 7) over 30 minutes run a flow rate of 0.5 ml min^{-1} (for one example see supplementary fig. 6). Three independent experiments were analyzed for each number of laser shots. In both cases, the percentage of remaining starting material was plotted against the irradiation dose (supplementary fig. **9**). The slopes of both curves was determined and the quantum yield of caged IP7 was determined using the formula shown in the supplementary equation 1:

$$\phi_{\boldsymbol{caged\ IP7}} = \phi_{reference} \times \frac{n_{sample,0} \times slope_{sample}}{n_{referenc,0} \times slope_{reference}} \qquad (1)$$

$$= 15\% \times \frac{3.37 \times 10^{-9} \mathrm{mol} \times (-0.02934)}{8.29 \times 10^{-9} \mathrm{mol} \times (-0.25034)} = 0.71\%$$

The detailed procedure has been described by G. Gasser et al.[2]

<u>Extraction and MS Analyses of metabolites from cells:</u>

Cellular Uptake & Stability Studies

This procedure describes, how Figure 3 in the main manuscript was obtained. It shows that the transporter **11** facilitates uptake of **9** (which is not taken up in the absence of **11**) and that **9** is stable in living cells. Moreover, photouncaging of **9** after transport by **11** in living cells is shown.

For *in cellulo* experiments, mammalian cells (HeLa) were grown up to 80–90% confluence (60mm dishes ≈1 X 10^6 cells) in DMEM media with 10% FCS, 200mM L-glycine, high glucose (4.5g/l) and 5% CO_2.

The media were removed from cells and cells washed with ~2mL of 1X PBS (2 times).Then, 1mL media (consisting of Compound **9** + DMEM, compound **9** and **11** or **11** only) were added to cell plate (containing 5mL DMEM media) and distributed equally (Final concentration of compound **9/11** should be 5 μM for each cell plate). Cells were incubated at 37^0C, 5% CO_2 from 5h to 16h.

For the different experiments, cells were either irradiated for 10 min with a UV lamp at 366 nm or directly extracted as described below.

After appropriate incubation time, excess media was removed and the cells washed with ~2 mL of 1X PBS (3times).Then, 1.5mL trypsin were added and incubated at 37^0C for 5 to 10 min. Cells were transferred into a 15mL falcon tube.

Cells were then centrifuged at 2200 rpm for 3–5 min and the supernatant was discarded and the washing step repeated twice. Inositolphosphate content was enriched on TiO_2 beads following the procedure by Saiaradi.[3]

Band Extraction from PAGE:

All analyses have been conducted with exclusion of ambient light.

The same procedure as described for PAGE analysis of InsP7 was applied as described.[1] A small portion of the gel from just above the Orange G dye band was cut to the bottom containing the $InsP_6$ standard and one sample as described previosly.[4] Briefly, the cut portion of the gel was stained with toluidine Blue (0.1% (w/v) toluidine blue, 20% (w/v) methanol, 2% (w/v) glycerol) for a few minutes (4-5min) or until the inositol pyrophosphate band appears. The glass plate previously removed was put back on top of the gel to prevent the unstained gel from drying.The stained portion of the gel was transferred in a de-staining solution (20% (w/v) methanol) for a few minutes to wash away any excess of toluidine blue and the gel was put next to the unstained gel. The 5-PP-$InsP_5$ and DEACM-$InsP_7$ **9** bands were cut from the unstained portion of the gel using as reference the $InsP_7$ migrating position determined with the stained gel. Each band that was cut from the gel was put in a 15mL tube separately and 10mL of deionized ddH_2O were added. The tubes were rotated for 10 minutes at room temperature. The liquid was discarded to remove excess of TBE and microscopic acrylamide particles. Subsequently, two dehydration-hydration cycles were performed: 5 mL of 50% (w/v) methanol were added to the tube with the gel containing 5-PP-$InsP_5$ and DEACM-5-$InsP_7$ **9** bands. The tubes were rotated at room temperature for 2 hours. The gel slices were transferred into a new 15 mL tube containing 5 mL of ddH_2O. The tubes were rotated at room temperature for 2 hours.

To concentrate the eluted compounds, the combined 10 mL pool of 5 mL ddH2O and 5 mL 50% (w/v) methanol were concentrated using a SpeedVac heated at 60°C for 5-8hrs. Once the samples were nearly dry, the remaining liquid (300-600 µl) was

transferred to a 1.5 mL centrifuge tube and spun for 2 minutes at 5000 rpm. The supernatant was collected and transferred into a fresh 1.5 mL centrifuge tube; 20-30 µL were left in the old tube and discarded,. If necessary, the drying process was continued using the SpeedVac without heating. The recovery of 5-PP-InsP$_5$ and DEACM 5-InsP$_7$ **9** was dramatically reduced if the samples dried completely. Termination of the drying process at a volume of 100-300 µL was necessary.

Mass spectrometric analysis

MALDI-MS analyses were performed in the negative ionization mode on a time-of-flight mass spectrometer operating in the linear mode (Autoflex equipped with a 337 nm N_2 laser, Bruker Daltonics, Bremen, Germany). The sample solutions described above (1 µL) were pipetted on a 9-aminoacridine solid layer (Fluka, ≥ 99.5%, Buchs, Switzerland) prepared on a stainless steel plate (0.5 µL of a 7 mg/mL acetonitrile/H_2O 1:1 solution).

Cytotoxicity Assay

In order to assess, whether cells survive UV irradiation for follow-up studies, HeLa cells were additionally irradiated for 5 minutes after uptake of **9**+**11** in a Rayonet reactor (6*14W bulbs; 2.58J/cm^2). Afterwards, cell viability was assessed using the Alamar Blue assay. No spontaneous cell death was observed after irradiation of cells that have taken up **9**+**11**.

HELA cells were seeded (4x10^3/well) into 96 well plate and incubated overnight in DMEM media with 10% FCS, 200mM L-glycine, high glucose (4.5g/l) and 5% CO_2.

DEACM-InsP$_7$: Co-oligomer (D:G 7:7) complexes were prepared in an Eppendorf cap (0.5µL 1mM DEACM-InsP$_7$ stock and 0.5µL 1 mM co-oligomer stock in PBS (1X) (total 10µL)) and incubate at room temperature for 30 min. Afterwards 90µL DMEM media were added.

Cells were then incubated with the DEACM-InsP$_7$:Co-oligomer (D:G7:7) complexes. at 37^{0}C, 5% CO_2 for 4 h (each condition in triplicate). After appropriate incubation time (4 h) excess media (DEACM-InsP$_7$:Co-oligomer (D:G7:7) complexes) was removed and the cells washed with 100µL of 1X PBS (2times). 100µL cell medium were added to each well. Cells were irradiated at 350 nm for 5 min in a Rayonet Chamber Reactor RPR200, (2.58J/cm^2; 6 light bulbs, 14 W each) and then the cells were incubated at 37^{0}C, 5% CO_2 for 15min. After this period, the medium was removed and 100µL of cell medium containing resazurin (final Conc. 0.2mg/ml) were

added and the cells were incubated at 37^{0}C, 5% CO_2 for 4 h. Fluorescence was quantified at 540nm excitation, 590nm emission in a SpectraMax M2 microplate reader. All experiments where run in triplicate (results indicated as average with standard deviation) and normalized to non-irradiated cells (100% cell viability).

General Remarks

Reagents were purchased from commercial suppliers (Acros, Aldrich, Fluka, TCI, ChemGenes Corp.) and used without further purification, unless noted otherwise.

Solvents were obtained in analytical grade and used as received for coupling reactions, extractions, chromatography and precipitation.

Dry solvents for reactions were purified by filtration and dried by passage over activated anhydrous neutral A-2 alumina (MBraun solvent purification system) under an atmosphere of dry N_2.

Deuterated solvents for NMR and reactions were obtained from Armar Chemicals, Switzerland, in the indicated purity grade and used as received for coupling reactions and NMR spectroscopy.

Phosphoramidites were synthesized using oven-dried glassware under an atmosphere of dry N_2 unless noted otherwise. Air- and moisture-sensitive liquids and solutions were transferred via syringe.

Thin Layer Chromatography was carried out using Merck silica gel 60 F254 plates, visualized with UV light or developed either with aqueous cerium ammonium molybdate stain or with *p*-Anisaldehyde stain followed by heating.

Flash Chromatography was performed using Fluka silica gel 60 (230-400 Mesh).

Lyophilizations were done with a Christ Freeze Dryer Alpha 1-2 LD+.

^{1}H-NMR spectra were recorded on Bruker 400 MHz spectrometers or Bruker 500 MHz spectrometers (equipped with a cryo platform) at 298K in the indicated deuterated solvent. Data are reported as follow: chemical shift (δ, ppm), multiplicity (s, singulet; d, doublet; t, triplet; q, quartet; m, multiplet or not resolved signal; br, broad signal), coupling constant(s) (*J*, Hz), integration. All signals were referenced to the internal solvent signal as standard ($CDCl_3$, δ 7.26; D_2O, δ 4.79; CD_3OD, δ 3.31; DMSO-d_6, δ 2.50).

^{31}P[^{1}H]-NMR spectra and ^{31}P-NMR spectra were recorded with ^{1}H-decoupling or ^{1}H couling on Bruker 162 MHz or Bruker 202 MHz spectrometers (equipped with a cryo platform) at 298K in the indicated deuterated solvent. All signals were referenced to an internal standard (PPP).

^{13}C[^{1}H]-NMR spectra were recorded with ^{1}H-decoupling on Bruker 101 MHz or Bruker 125 MHz spectrometers (equipped with a cryo platform) at 298K in the indicated deuterated solvent. All signals were referenced to the internal solvent signal as standard ($CDCl_3$, δ77.0; CD_3OD, δ 49.0; DMSO-d_6, δ 39.5).

UV-Vis measurements were carried out on a Agilent 8453 UV-Vis spectrophotometer.

Mass spectra were recorded by the Mass spectroscopy Service of UZH on Finnigan MAT95 MS, Bruker EsquireLC MS , Bruker maXis QTof HR MS and Finnigan TSQ700 MS machines.

HPLC (reverse-phase) was performed with a Varian Pro Star Solvent Delivery Module (model 210) equipped with a Waters XBridge BEH C_{18} Column (130Å, 3.5 µm, 4.6 x 150 mm) and coupled to a Varian Pro Star UV/Vis detector. The mobile phase consisted of 0.1 M aqueous triethylammonium acetate (TEAA) buffer at pH 7 and increasing amounts of MeCN. The products were detected at λ = 260 nm.

Biological Materials Dulbecco's Modified Eagle's Medium (DMEM) was purchased from Invitrogen, and supplemented with 10% FBS and 1% penicillin/streptomycin. Phosphate-buffered saline pH 7.4. HeLa and HCT cells were maintained in DMEM at 37 °C in a 5% CO_2 atmosphere.

Flow Cytometry Analysis (FACS) was performed on a BD LSRII FACS Analyzer using a 450 nm Violet laser (LSRII.UV, obtained using funds from NIH Shared Instrumentation Grant S10RR027431-01, Stanford University Shared FACS Facility).

Flash Photolysis was carried out with an Edinburgh Instruments LP920 laser flash photolysis setup equipped with a Continuum Surelite Nd:YAG laser, frequency-tripled to generate monochromatic (*i.e.* bandwidth of less than 1 nm) light with a wavelength of 355 nm.

Polyacrylamide Gel Electrophoresis (PAGE) was carried out by employing 24cm long; 18cm wide glass plates, 1.0mm wide spacers and 15-lane comb were used for loading the samples.

Confocal Microscopy was conducted on a CLSM SP5 Mid UV-VIS Leica inverted confocal laser-scanning microscope.

Chemical Synthesis

Synthesis of **2**

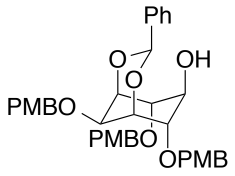

The compound was synthesized as described before in three steps starting from *myo*-inositol. Analytical data were identical with the values reported in the literature. [5]

Synthesis of **3b**

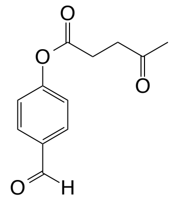

3.76 g (31.6 mmol, 1.00 eq.) of Levulinic acid and 3.90 g (31.9 mmol, 1.00 eq.) 4-Hydroxy benzaldehyde **3a** were dissolved in CH_2Cl_2 (30 ml) and DMF (4 ml) and cooled to 0ºC. 7.21g (34.9 mmol, 1.10 eq.) DCC and 0.190 g (2.00 µmol, 0.050 eq) DMAP were added to the solution. The mixture was stirred for 30 min at 0° and 2 h at r.t. until TLC showed total conversion. Hexane and Et_2O (1:1, 30 ml) were added to the crude, and a white precipitate was filtrated. Extraction with Et_2O (80 ml), washing of the org. layer with a ac. soln. of $NaHCO_2$ (sat., 2 X 80 ml) and H_2O (2 X 80 ml), drying of the combined phases with Mg_2SO_4. After evaporation a crude product was obtained (5.79 g), which was chromatographed (SiO_2, 5 g, EtOAc:Pentane, 4:1) to afford **3b** (5.46 g, 24.8 mmol, 77 %).

TLC (EtOAc): $\mathbf{R_f}$ = 0.70; ^{1}**H-NMR** (300 MHz, $CDCl_3$): δ 9.98 (s, 1H), 7.91 (d, *J* = 8.7 Hz, 1H), 7.28 (d, *J* = 8.5 Hz, 2H), 3.10 - 2.58 (m, 4H), 2.23 (s, 3H); ^{13}**C-NMR** (126 MHz, $CDCl_3$): δ 206.31, 190.97, 170.84, 155.37, 133.97, 131.18, 122.34, 37.84, 29.79, 28.16; **IR** (neat, cm^{-1}) 3403.7, 2926.5, 2360.4, 1753.9, 1714.4, 1603.5, 1507.1, 1360.5, 1197.6, 1135.9, 1014.4, 843.7; **HRMS** (ESI) $[M+Na]^+$ calcd for $C_{12}H_{12}NaO_4$, 243.0633; found, 243.0627

Synthesis of **3c**

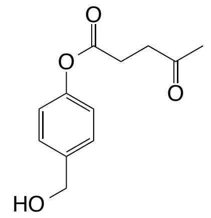

5.22 g (23.7 mmol, 1.00 eq) of **3b** was dissolved in 17.1 g (0.284 mol, 12.0 eq.) of *i*prOH. 4.84 g (23.7 mmol, 1.00 eq.) Al(O*i*Pr)$_3$ was added to the solution. The mixture was stirred at r.t. for 22 h until TLC showed no remaining **3b**. The mixture was extracted with Et_2O (80 ml), the org. layer washed with H_2O (3 x 80 ml), dried with Mg_2SO_4. Evaporation of the solvent delivered a crude product (3.78 g), which was chromatographed (SiO_2, EtOAc:Pentane, 4:1) to afford **3c** (5.05 g, 19.2 mmol, 81 %) as a yellowish oil.

TLC (EtOAc:Hexane, 1:1 v/v): $\mathbf{R_f}$ = 0.43; ^{1}**H-NMR** (300 MHz, $CDCl_3$): δ 7.36 (d, *J* = 8.5 Hz, 1H), 7.07 (d, *J* = 8.6 Hz, 1H), 4.65 (s, 1H), 2.91 - 2.78 (m, 4H), 2.23 (s, 2H); ^{13}**C-NMR** (126 MHz, $CDCl_3$): δ 206.68, 171.59 , 149.95 , 138.66 , 128.00 , 121.54 , 64.54 , 37.92 , 29.85 , 28.14; **IR** (neat, cm^{-1}): 2929.3, 2360.4, 1753.0, 1714.4, 1698.0, 1601.6, 1413.6, 1356.7, 1293.0, 1205.3, 1124.3, 733.8; **HRMS** (ESI) $[M+Na]^+$ calcd for $C_{12}H_{14}NaO_4$, 245.0790; found, 245.0785

Synthesis of **3**

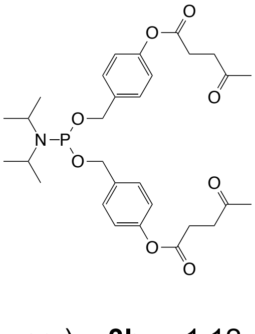

2.59 g (11.6 mmol, 2.10 eq.) **3b**, 1.12 g (5.52 mmol, 1.00 eq.) (dichlorophosphanyl)bis(propan-2-yl)amine and 1.23 g (12.2 mmol, 2.20 eq.) triethylamine were stirred overnight at room temperature under an inert atmosphere in dry THF (32 mL). The precipitate was filtered off, rinsed with Et_2O and the solution concentrated in vacuo to give a brownish oil 3.02 g **3** (5.26 mmol, 95%) that did not require further purification.

TLC (EtOAc:Hexane, 1:4 v/v): $\mathbf{R_f}$ = 0.45; **^{1}H-NMR** (500 MHz, $CDCl_3$): δ 7.35 (d, *J* = 8.5 Hz, 4H), 7.06 (d, *J* = 8.5 Hz, 4H), 4.80- 4.63 (m, 4H), 2.91- 2.79 (m, 8H), 2.23 (s, 6H), 1.27 (m, 2H), 1.30- 1.23 (d, *J* = 6.8 Hz, 12H); **^{13}C-NMR** (126 MHz, $CDCl_3$) δ 206.37, 171.44, 149.79, 137.08 (d, *J* = 7.5 Hz), 129.23, 128.73, 127.97, 121.27, 65.21- 64.34 (m), 43.10 (d, *J* = 12.4 Hz), 37.91 (d, *J* = 2.4 Hz), 29.86, 28.16, 24.63 (d, *J* = 7.2 Hz); **^{31}P{1H}-NMR** (162 MHz, $CDCl_3$): δ 149.16; **^{31}P-NMR** (162 MHz, $CDCl_3$): δ 149.16 (p, *J* = 9.1 Hz); **IR** (neat, cm^{-1}): 2967.0, 1455.0,1180.2, 1027.9, 978.7, 760.8, 700.0, 423.3; **HRMS** (ESI) $[M+Na]^+$ calcd for $C_{30}H_{40}NNaO_8P$, 596.2376; found, 596.2389.

Synthesis of **4**

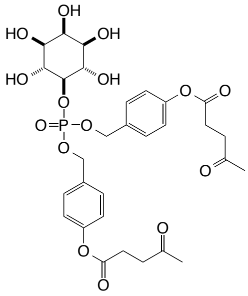

A) 1.00 g (1.59 mmol, 1.00 eq.) **2** and 1.26 g (2.08 mmol, 1.30 eq) levulinylbenzyl phosphoramidite (LevB-PA) **3** were dissolved in dry MeCN (5 mL). 290 mg (2.22 mmol, 1.40 eq) of 4,5-dicyanoimidazole was added and mixture was stirred for 10 minutes at room temperature. Progress of the reaction was monitored by ^{31}P-NMR. Oxidation was achieved by addition of 1.64 ml (7.95 mmol, 5.00 eq) *t*-BuOOH. The solvent was evaporated and the obtained crude oil was directly used in the next step.

B) The intermediate was dissolved in DCM (50 mL) and 5% of TFA (2.5 ml) and stirred for 45 min at room temperature. The reaction was monitored by TLC and ^{31}P-NMR. After completion of the reaction, the solvent was removed *in vacuo* and the residue was crystalized from Et_2O and purified by column chromatography (gradient EtOAc to EtOAc: MeOH, 1:1) yielding the product as a brownish sticky solid **4** (580 mg, 0.868 mmol, 55 % yield).

TLC (CH_2Cl_2:MeOH, 4:1 v/v): $\mathbf{R_f}$ = 0.23; **^{1}H-NMR** (400 MHz, DMSO-d_6): δ 7.44 (d, *J* = 8.6 Hz, 4H), 7.09 (d, *J* = 8.5 Hz, 4H), 5.11 (d, *J* = 6.8 Hz, 4H), 5.03 (d, *J* = 5.5 Hz, 2H), 4.70 (d, *J* = 3.6 Hz, 1H), 4.66 (d, *J* = 5.9 Hz, 2H), 4.03 (dd, *J* = 18.1, 9.0 Hz, 1H), 3.76 (q, *J* = 2.9 Hz, 1H), 3.71- 3.60 (m, 2H), 3.31- 3.21 (m, 3H), 2.93 - 2.81 (m, 4H), 2.81 - 2.70 (m, 4H), 2.16 (s, 5H), 1.28 - 1.15 (m, 3H); **^{13}C-NMR** (101 MHz, DMSO-

d_6): δ 207.27, 171.75 , 150.67 , 134.69 (d, J = 8.1 Hz), 129.51 , 122.05 , 83.85 (d, J = 7.1 Hz), 72.90 , 72.00 , 71.81 (d, J = 3.0 Hz), 71.79 , 68.02 (d), 40.52 (d, J = 21.1 Hz), 37.98 , 29.99 , 28.29 , 27.63; ^{31}**P{1 H}-NMR** (162 MHz, DMSO-d_6): δ -0.20 (s); ^{31}**P-NMR** (162 MHz, 300 K, DMSO-d_6): δ 0.02 to -0.45 (m); **IR** (neat, cm-1) 3340.1, 2924.5, 1753.9, 1715.4, 1676.8, 1509.0, 1361.5, 1199.5, 1133.9, 1009.6, 718.4; **HRMS** (ESI) $[M+Na]^+$ calcd for $C_{30}H_{37}NaO_{15}P$, 691.1758; found, 691.1762

Synthesis of **6**

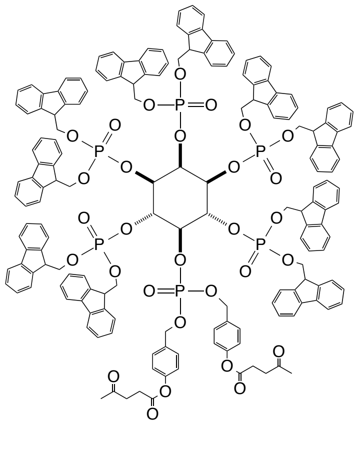

100 mg (0.150 mmol, 1.00 eq.) of inositol phosphate triester **4** and 1.15 g (2.25 mmol, 15.0 eq.) of 9-fluorenylmethyl phosphoramidite (Fm-PA) **5** were coevaporated with dry MeCN (2 mL). The residue was dissolved in dry MeCN (4 mL). To this solution 353 mg (2.99 mmol, 20.0 eq.) of DCI was added. Progress of the reaction was monitored by ^{31}P-NMR. After completion of the reaction (30-45 min), oxidation was achieved by slow (!) addition of 553 mg (2.25 mmol, 15.0 eq.) mCPBA (70% moistened with water) at 0ºC. The mixture was concentrated *in vacuo* and the product was crystalized from MeOH (2 x 5 ml) yielding 315 mg of **6** as a white sticky solid (0.110 mmol, 74%).

TLC (EtOAc:Hexane, 3:2 v/v): $\mathbf{R_f}$ = 0.33; ^{1}**H-NMR** (400 MHz, $CDCl_3$): δ 7.56 (s, 1H), 7.54- 7.47 (m, 12H), 7.47- 7.42 (m, 7H), 7.39 (d, J = 7.6 Hz, 3H), 7.32 (dt, J = 12.9, 7.8 Hz, 8H), 7.24 (d, J = 8.4 Hz, 5H), 7.21 (d, J = 3.4 Hz, 2H), 7.19 (s, 1H), 7.19- 7.12 (m, 10H), 7.12- 7.06 (m, 9H), 6.98 (dq, J = 31.2, 7.6, 7.0 Hz, 25H), 6.76 (d, J = 8.2 Hz, 3H), 4.94 (q, J = 9.8 Hz, 2H), 4.87 (dd, J = 11.8, 7.7 Hz, 1H), 4.66 (t, J = 10.8 Hz, 1H), 4.47 (dd, J = 10.2, 5.6 Hz, 2H), 4.37 (q, J = 7.7 Hz, 2H), 4.27 (qd, J = 13.7, 9.0, 7.5 Hz, 5H), 4.24- 4.17 (m, 4H), 4.17 - 4.11 (m, 2H), 4.04 (h, J = 6.9 Hz, 4H), 3.97 (dt, J = 13.9, 7.1 Hz, 4H), 3.88 (dq, J = 12.1, 6.7, 5.8 Hz, 6H), 3.74 (q, J = 7.1 Hz, 3H), 2.74 (d, J = 5.5 Hz, 2H), 2.71 (d, J = 5.4 Hz, 3H), 2.12 (s, 4H), 1.16 (d, J = 6.5 Hz, 2H), 0.94 (s, 1H); ^{13}**C-NMR** (126 MHz, $CDCl_3$): δ 206.83 , 171.64 , 151.25 ,

144.15 - 143.30 (m), 142.22 - 141.61 (m), 133.89 (d, *J* = 6.4 Hz), 128.24 (q, *J* = 6.8, 5.7 Hz), 127.59 (d, *J* = 22.1), 126.06 (d, *J* = 31.0), 122.11 , 120.90 - 120.06 (m), 76.53 , 75.33 , 74.79 , 73.90 , 70.93 - 69.89 (m), 69.70 (d, *J* = 5.6 Hz), 48.91 - 47.87 (m), 38.49 , 30.44 , 28.75 (d, *J* = 3.2 Hz), 27.95; **^{31}P{1 H}-NMR** (162 MHz, $CDCl_3$) δ 0.76 (s), 0.36 (s), -0.38 (s), -1.75 (s); **^{31}P-NMR** (162 MHz, $CDCl_3$): δ 0.94- 0.59 (m), 0.57- 0.17 (m), -0.38 (q, *J* = 7.0 Hz), -1.56- -1.92 (m); **IR** (neat, cm-1) 2923.6, 2853.2, 2362.4, 2341.2, 1709.6, 1449.2, 1283.4, 1020.2, 983.5; **HRMS** (ESI) $[M+2Na]^{2+}$ calcd for $C_{170}H_{142}Na_2O_{30}P_6$, 1447.3898; found, 1447.3914.

Synthesis of **7**

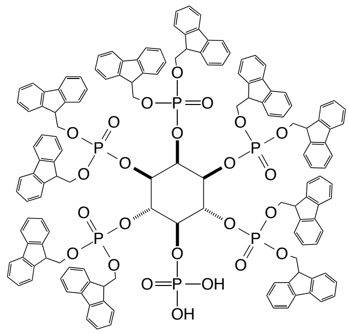

150 mg (52.6 μmol, 1.00 eq.) of hexaphosphate **6** was dissolved in CH_2Cl_2 (2.60 mL). TFA (42.2 μL) and mixture of hydrazine acetate 9.75 mg (0.30 mmol, 6.00 eq) in MeOH (0.260 mL) were added. Reaction mixture was stirred overnight at room temperature. It was tracked by ^{31}P-NMR. After completion it was concentrated in vacuo and precipitated from MeOH, crude material **7** was obtained (115 mg, 89%) and it was used directly in the next step.

TLC (EtOAc): **R_f** = 0.43; **^{31}P{1 H}-NMR** (162 MHz, $CDCl_3$): δ 2.48 , -0.20 , -0.66; **^{31}P-NMR** (162 MHz, $CDCl_3$): δ 2.48 (d, *J* = 13.9 Hz), 0.03- -0.40 (m), -0.51- -0.80 (m); **HRMS** (ESI) $[M]^-$ calcd for $C_{146}H_{117}O_{24}P_6$, 2439.6364; found, 2439.6366

Synthesis of **7a**

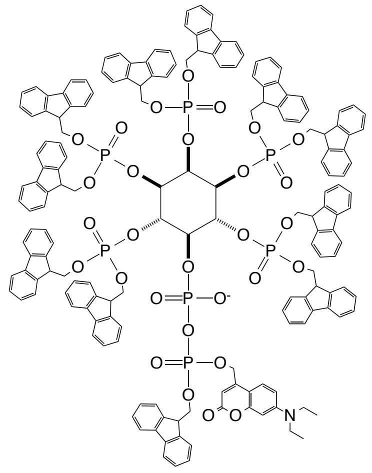

70.0 mg (28.6 µmol, 1.00 eq.) of crude hexaphosphate **7** were dissolved in dry CH_2Cl_2 (2 mL). 33 .0 mg (57.2 µmol, 2.00 eq.) coumarine-9-florenylmethyl phosphoramidite (Coum-Fm-PA) **8** was added and the mixture was coevaporated. The dry mixture was dissolved again in dry CH_2Cl_2 (3 mL) and 10.3 mg (85.8 µmol, 2.00 eq.) 4,5-dicyanoimidazole was added. The reaction was stirred for 10 minutes. Progress of the reaction was monitored by ^{31}P-NMR. After completion of the reaction, oxidation was achieved by slow (!) addition of 14.1 mg (57.2 µmol, 2.00 eq) *m*CPBA (70% moistened with water).

The reaction mixture was concentrated and MeOH (5 ml) was added. A colorless solid precipitated and it was isolated by centrifugation for 5 min. The supernatant was removed and the precipitate purified by column chromatography (EtOAc:hexane 1:1 over EtOAc to EtOAc:MeOH=20:1). Yield: 44 mg **7a** as a colorless syrup (15 µmol, 52%, mixture of diastereomers).

TLC (EtOAc): **R_f** = 0.66; **^{1}H-NMR** (400 MHz, $CDCl_3$): δ 8.40 (s, 1H), 8.31 (d, *J* = 8.0 Hz, 2H), 8.03-7.89 (m, 4H), 7.81- 7.64 (m, 5H), 7.62- 7.47 (m, 12H), 7.51- 7.41 (m, 12H), 7.37 (m, 7H), 7.28- 7.16 (m, 6H), 7.19-7.05 (m, 14H), 7.08- 6.94 (m, 12H), 6.96-6.78 (m, 11H), 6.81-6.70 (m, 4H), 6.70-6.61 (m, 3H), 6.63-6.54 (m, 2H), 6.26 (s, 1H), 6.15-6-00 (m, 2H), 5.78 (dd, *J* = 32.3, 8.7 Hz, 2H), 5.58 (s, 2H), 5.14 (s, 4H), 4.40 (s, 4H), 4.26 (s, 6H), 4.06 (d, *J* = 23.5 Hz, 7H), 4.00-3.59 (m, 14H), 3.57-3.31 (m, 6H), 3.06-2.93 (m, 4H), 1.08 (t, *J* = 7.2 Hz, 3H), 1.01-0.78 (m, 8H); **^{13}C-NMR** (126 MHz, $CDCl_3$): δ 155.7 (s), 150.1 (s), 143.4-142.4 (m), 141.1-140.4 (m), 127.8-126.9 (m), 125.7-124.8 (m) , 119.7-118.8 (m), 76.2 (s), 75.8 (s), 73.3 (s), 71.1 (s), 70.1-68.7 (m), 48.52-46.58 (m), 12.36 (s); **^{31}P{1 H}-NMR** (162 MHz, $CDCl_3$): δ 1.83, -0.62 , -0.96 , -1.71 , -2.25 , -7.38 (d, *J* = 14.9 Hz), -8.90 (d, *J* = 15.1 Hz); **^{31}P-NMR** (162

MHz, $CDCl_3$): δ 2.03-1.63 (m), -0.62 (ddt, *J* = 15.6, 10.3, 5.2 Hz), -0.78 to -1.13 (m), -1.49 to -1.90 (m), -2.03 to -2.49 (m), -7.19 to -7.58 (m), -8.72 to -9.08 (m); **IR** (neat, cm-1) 3040.2, 2954.4, 2924.5, 2337.3, 1717.3, 1606.4, 1449.2, 1280.5, 1024.0, 986.4; **HRMS** (ESI) $[M]^+$ calcd for $C_{174}H_{143}NO_{29}P_7$, 2926.7915; found, 2926.7882.

Synthesis of **7b**

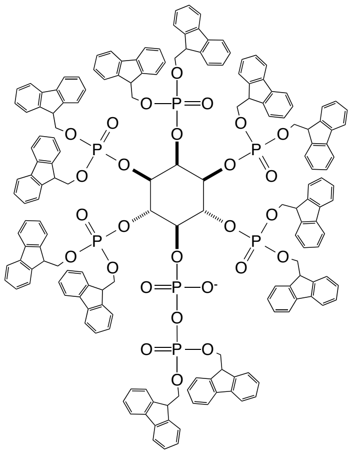

50.0 mg (20.4 µmol, 1.00 eq.) of crude hexaphosphate **7** were dissolved in dry CH_2Cl_2 (2 mL). 9-Fluorenylmethyl phosphoramidite (Fm-PA) **5** 21.0 mg (40.9 µmol, 2.00 eq) was added and the mixture was coevaporated. The dried mixture was dissolved again in CH_2Cl_2 (2 mL) and DCI 4.83 mg (40.9 µmol, 2.00 eq.) was added. The reaction was stirred for 10 minutes. Progress of the reaction was monitored by ^{31}P-NMR. After completion of the reaction, oxidation was achieved by slow (!) addition of 7.1 mg (41 µmol, 2.0 eq) *m*CPBA (70% moistened with water).

The reaction mixture was concentrated and MeOH (5 ml) was added. A white solid precipitated and it was isolated by centrifugation for 5 min. The solvent was removed and the precipitate purified by column chromatography (EtOAc:hexane 1:1 over EtOAc to EtOAc:MeOH=20:1). Yield: 20 mg **7b** as a colorless syrup (7.0 µmol, 34%).

TLC (EtOAc): $\mathbf{R_f}$ = 0.60; **^{1}H-NMR** (500 MHz, $CDCl_3$): δ 8.03- 6.38 (m, 96H), 5.63-5.23 (m, 5H), 5.16- 3.34 (m, 25H), 2.32- 2.09 (m, 12H); **^{13}C-NMR** (126 MHz, $CDCl_3$): δ 144.31- 140.10 (m), 128.47- 123.93 (m), 120.63- 118.69 (m), 75.55 , 73.19 , 72.24-67.67 (m), 53.36 , 49.36- 45.73 (m), 30.82 (s), 29.67; **^{31}P{1 H}-NMR** (203 MHz, $CDCl_3$): δ -0.33 , -2.01 , -2.15 , -2.74 , -3.81 , -7.47 , -9.42; **^{31}P-NMR** (203 MHz, $CDCl_3$): δ -0.32 , -2.00 , -2.74 , -3.80 , -7.47 , -9.45 .; **IR** (neat, cm-1) 3040.2, 2954.4, 2924.5, 2337.3, 1717.3, 1606.4, 1449.2, 1280.5, 1024.0, 986.4; **HRMS** (ESI) $[M]^+$ calcd for $C_{174}H_{138}O_{27}P_7$, 2875.7594; found, 2875.7594.

Synthesis of **8**

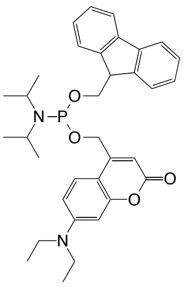

The compound was synthesized as described before in three steps starting from *myo*-inositol. Analytical data were identical with the values reported in the literature.[6]

Synthesis of **9**

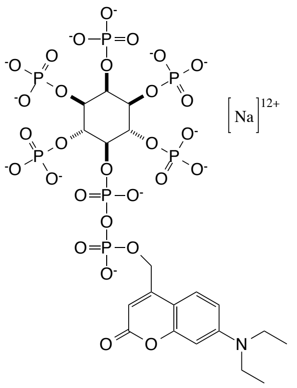

29.0 mg (9.91 µmol, 1.00 eq.) of **7a** were dissolved in DMF (3 mL) and piperidine (0.5 ml) was added. The solution was stirred 40 minutes at room temperature. After completion of the deprotection, the solution was concentrated and the product precipitated with Et_2O (10 mL). The precipitate was isolated by centrifugation and the supernatant discarded. The precipitate was redissolved in MeOH and crystallized by addition of Et_2O. The centrifugation process was repeated and yellowish crystals were obtained that were dried in vacuo. Piperidinium counter ions were exchanged to sodium by addition of excess NaI to a MeOH solution of **9**. After 30 minutes of stirring the sodium salt of **9** precipitated: Yield: 17.1 mg of **9** (8.59 µmol, 87%). Data as piperidinium salt, ^{1}H-NMR spectra in both piperidine and sodium form.

^{1}H-NMR (400 MHz, MeOD): δ 7.65 (d, *J* = 9.0 Hz, 1H), 6.80 (dd, *J* = 9.1, 2.6 Hz, 1H), 6.54 (s, 1H), 6.31 (s, 1H), 5.32 (d, *J* = 7.0 Hz, 2H), 5.07- 4.98 (m, 1H), 4.68- 4.53 (m, 2H), 4.44 (q, *J* = 9.4 Hz, 1H), 4.34- 4.18 (m, 2H), 3.50 (q, *J* = 7.0 Hz, 4H), 3.18 (piperidine), 1.82 (piperidine), 1.67 (piperidine), 1.23 (t, *J* = 7.0 Hz, 6H); **^{13}C-NMR**

(126 MHz, MeOD): δ 163.83, 156.56, 151.52, 125.88, 109.75, 106.48, 104.92, 97.26, 78.87, 76.68, 75.64, 74.31, 48.69, 48.55, 48.52, 48.46, 48.39, 48.38, 48.35, 48.25, 48.21, 48.18, 48.04, 48.01, 47.87, 47.84, 47.67, 44.67, 22.97, 22.59, 11.96; **^{31}P{1 H}-NMR** (162 MHz, MeOD): δ 2.93, 2,56, 2,12, -9.08 (d, J=9.1), -10.16 (d, J=9.1); **^{31}P-NMR** (162 MHz, MeOD): δ 2.93 (d, J=9.1), 2.58, 2.12 (d, J=10.0), -9.10 (d, J=9.1), -10.10 (d, J=9.1); **IR** (neat, cm^{-1}) 3370.0, 2961.2, 2863.8, 2363.3, 1701.9, 1603.5, 1461,8, 1030.8, 933.4, 514.9; **HRMS** (ESI) $[M+Na]^+$ calcd for $C_{20}H_{32}NNa_3O_{29}P_7$, 1035.8911; found, 1035.8904; $[M]^{3-}$ calcd for $C_{20}H_{31}NO_{29}P_7^{3-}$, 321.9721; found, 321.9725.

Synthesis of **10**

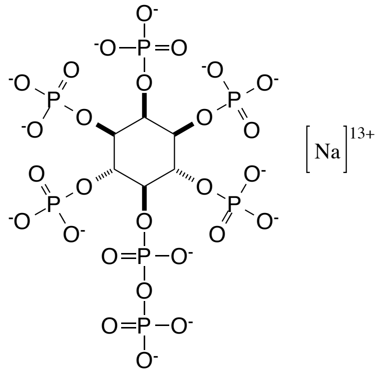

25.0 mg (8.69 µmol, 1.00 eq.) of **7b** were dissolved in DMF (3 mL) and piperidine (0.5 ml) was added. The solution was stirred 40 minutes at room temperature. After completion of the deprotection, the solution was concentrated *in vacuo* and the product precipitated by addition of Et_2O (10 mL). The precipitate was isolated by centrifugation and the supernatant discarded. The precipitate was once more dissolved in MeOH and crystallized by addition of Et_2O. The centrifugation process was repeated and yellowish crystals were obtained that were dried in vacuo. Piperidinium counter ions were exchanged to sodium by addition of excess NaI to a MeOH solution of **10**. After 30 minutes of stirring, the sodium salt of **9** precipitated as colorless crystals: Yield: 7 mg of **10** (7 µmol, 80%).

^{1}H-NMR (400 MHz, D_2O): δ 4.74 (s, 3H), 4.39 (q, *J* = 12.0, 9.2 Hz, 2H), 4.17 (q, *J* = 9.7 Hz, 1H), 4.03 (s, 3H); **^{13}C-NMR** (126 MHz, D_2O): δ 78.06, 75.92, 75.44, 74.00; **^{31}P{1 H}-NMR** (162 MHz, D_2O): δ 2.96 , 2.81 , 2.08 , -7.86 (d, *J* = 20.0 Hz), -8.94 (d, *J* = 20.6 Hz); **^{31}P-NMR** (162 MHz, D_2O): δ 2.96 (d, *J* = 8.7 Hz), 2.93- 2.69 (m), 2.08 (d, *J* = 10.3 Hz), -7.85 (d, *J* = 18.8 Hz), -8.94 (dd, *J* = 20.7, 8.7 Hz); **IR** (neat, cm-1): 3487.7, 3426.9, 2976.6, 2951.5, 1621.8, 1605.5, 1101.2, 977.7; **HRMS** (ESI) $[M]^{2-}$ calcd for $C_6H_{17}O_{27}P_7$, 368.9066; found, 368.9067

Supplementary References

1. Pisani F., Livermore T., Rose G., Chubb J.R., Gaspari M., et al. *PLoS ONE*, **9**(1): e85533. (2014).

2. Anstaett P., Leonidova A., Gasser G.. *Chem.Phys.Chem.* **16**, 1857-1860 (2015).

3. Wilson M. S. C., Bulley S. J., Pisani F., Irvine R. F., Saiardi A. *Open Biol.* DOI: 10.1098/rsob.150014. (2015).

4. Loss O., Azevedo C., Szijgyarto Z., Bosch D., Saiardi A. *J. Vis. Exp.* **55**, e3027, (2011).

5. Capolicchio S., Thakor D. T., Linden A., Jessen, H. J. *Angew. Chem. Int. Ed.* **52**, 6912-6916, (2013).

6. Subramanian D., Laketa V., Muller R., Ticher C., Zarbakhsh S., Pepperkok R., Schultz C. *Nat. Chem. Biol.* **6**, 324-326, (2010).
